# In vitro and clinical data analysis of Osteopontin as a prognostic indicator in colorectal cancer

**DOI:** 10.1111/jcmm.13686

**Published:** 2018-05-30

**Authors:** Ran Wei, Janet Pik Ching Wong, Peng Lyu, Xinping Xi, Olivia Tong, Shu‐Dong Zhang, Hiu Fung Yuen, Senji Shirasawa, Hang Fai Kwok

**Affiliations:** ^1^ Faculty of Health Sciences University of Macau Taipa Macau; ^2^ Northern Ireland Centre for Stratified Medicine Biomedical Sciences Research Institute University of Ulster Londonderry UK; ^3^ Institute of Molecular and Cell Biology A*STAR Singapore City Singapore; ^4^ Department of Cell Biology Faculty of Medicine Fukuoka University Fukuoka Japan

**Keywords:** colorectal cancer, connectivity mapping, Osteopontin, survival

## Abstract

Osteopontin (OPN) has been shown to promote colorectal cancer (CRC) progression; however, the mechanism of OPN‐induced CRC progression is largely unknown. In this study, we found that OPN overexpression led to enhanced anchorage‐independent growth, cell migration and invasion in *KRAS* gene mutant cells but to a lesser extent in *KRAS* wild‐type cells. OPN overexpression also induced PI3K signalling, expression of Snail and Matrix metallopeptidase 9 (MMP9), and suppressed the expression of E‐cadherin in *KRAS* mutant cells. In human CRC specimens, a high‐level expression of OPN significantly predicted poorer survival in CRC patients and OPN expression was positively correlated with MMP9 expression, and negatively correlated with E‐cadherin expression. Furthermore, we have found that 15 genes were co‐upregulated in OPN highly expression CRC and a list of candidate drugs that may have potential to reverse the secreted phosphoprotein 1 (*SPP1*) gene signature by connectivity mapping. In summary, OPN is a potential prognostic indicator and therapeutic target for colon cancer.

## 1 INTRODUCTION

Colorectal cancer (CRC) is the third most common malignancy worldwide, it has been reported that about 1.3 million cases of CRC are diagnosed every year.^1^ The tumour development involves multi‐steps process over years and mostly occurs in the ageing population.^2^ Despite traditional surgery, chemotherapy and radiotherapy, the prognosis of CRC remains poor. Some specific gene mutation leads to tumour aggression in CRC. *KRAS* gene mutation is commonly observed in the early stages of the adenoma‐adenocarcinoma sequence in human CRC development.^3^, ^4^ *KRAS* mutations occur in 30%‐40% of all cases of CRC. However, to date, specific therapeutic agents against *KRAS*‐mutated CRC have not been developed.^4^ The potential correlation between *KRAS* and other oncogene remains to be further investigated. Osteopontin (OPN) is a 34 kD integrin‐binding glycophosphoprotein, which plays an important role in cancer progression and is considered as a potential biomarker for cancer prognosis.^5^, ^6^ It is encoded by *SPP1* gene that has five isoform variants, which include OPN‐a, OPN‐b, OPN‐c, isoform 4 and isoform 5.^5^ OPN has been previously found to be highly expressed in colon cancer cells or tissues than that in normal intestinal epithelial cell line or corresponding normal colon tissues.^7^ Up‐regulation of OPN increased cell motility in vitro, tumorigenesis and angiogenesis in vivo,^8^ and predicted patient survival in colon cancer.^9^ Therefore, OPN has been recognized as a potential target for human colon cancer.^10^ However, the upstream and downstream effectors in OPN overexpression (OPN OE) of colon cancer are still largely unknown. In this study, we investigated the role of OPN overexpression related to phenotypic changes using *KRAS* mutant and *KRAS* wild‐type colon cancer cells. We further identified potential downstream targets involved in OPN overexpression‐mediated colon cancer progression. In addition, we also carried out the connectivity mapping analysis to identify potential therapeutic drug candidates for OPN‐overexpressing colon cancer.

## 2 MATERIALS AND METHODS

### 2.1 Cell culture

Isogenic pair of Duke Stage C colorectal cancer cells DLD1 with DKS8 were selected. DLD1 expresses heterozygous *Kras* G13D mutation and the isogenic cell, DKS8 had the *Kras* G13D mutation knocked out at its endogenous locus.^11^ The parental (DLD1), normal epithelial (FHC) and Phoenix‐AMPHO cell lines purchased from ATCC, while the isogenic cell lines were gifts from Prof. Senji Shirasawa's Laboratory. These cell lines were grown in cell culture media DMEM/F‐12 (Gibco: Cat. no. 1320033) with 10% foetal bovine serum and 1% penicillin/streptomycin. FHC medium prepared with additional 10 mmol/L HEPES, 10 ng/mL cholera toxin, 5 ng/mL transferrin, 5 ng/mL insulin and 100 ng/mL hydrocortisone.

### 2.2 Retroviral Infection and stable cell line transfection

The p3XFLAG‐CMV‐7.1 (Sigma, E7533) was ligated to the N‐terminal of OPN‐c gene of pcDNA3‐OPN‐V5, which was a gift from Steven Johnson (Addgene plasmid # 11617).^12^ Then it was inserted into pBABE‐puro which was a gift from Hartmut Land, Jay Morgenstern and Bob Weinberg (Addgene plasmid # 1764).^13^ Phoenix‐AMPHO cells were used to produce the retrovirus to transduce the DLD1 and DKS8. The expression level of OPN in these stable cell lines was determined with Western blotting.

### 2.3 Total protein quantification and Western blotting

The cell line samples were homogenized with ice‐cold RIPA lysis buffer that was added with protease inhibitor (Complete EDTA‐free, #10634200, Roche) and phosphatase inhibitor (PhosSTOP, #04906837001, Roche) and centrifuged at 20 000× g for 30 minutes at 4°C. Supernatants were collected and kept in −80°C. BCA method was used to quantify the protein concentration required for Western blot sample loading (Pierce BCA Protein Assay Kit, #23225). All samples were dissolved in LDS sample buffer and reducing agent (Life Technologies) and heated for 5 minutes at 95°C. An equal concentration of proteins was electrophorized and separated with TGX Stain‐Free™ FastCast™ Acrylamide Kit, 12% and transferred to a 0.2 μm nitrocellulose membrane (#IB401002) membranes. Blocking was carried out with 5% non‐fat milk, 0.1% Tween 20 in PBS for non‐specific binding for an hour at room temperature. The membranes were incubated overnight at 4°C with respective antibodies: anti‐FLAG (1:2000, F1804, Sigma), anti‐MMP9, 2c3, (1:500, sc‐21733, Santa Cruz Biotechnology), anti‐β‐catenin (1:500; Abcam, Cambridge, MA), anti‐Akt antibody (Cat. 9272, Cell Signalling), anti‐p‐Akt antibody (1:1000, Cat. 9271, Cell Signalling), anti‐Phospho‐GSK‐3β (Ser9) (1:1000, Cat.5558, Cell Signalling), anti‐GSK‐3β (1:1000, Cat.9315, Cell Signalling), anti‐Snail antibody (1:1000, ab180714),anti‐E‐Cadherin (24E10) (1:1000, Cat. 3195, Cell Signalling), anti‐N‐Cadherin (D4R1H) (1:1000, Cat. 13116, Cell Signalling) and normalized with anti‐β‐actin, C4 (1:5000, sc‐47778, Santa Cruz Biotechnology). The membranes were washed, incubated for an hour at room temperature with respective secondary antibodies and detected with chemiluminescent HRP substrate reagent (1:1) (Immobilon Western, WBKLS0500, Millipore) using ChemiDoc MP Imaging System (Bio‐Rad).

### 2.4 Soft agar colony formation assay

Soft agar assays were carried out in a 6‐well plate, each sample in triplicate and colonies were counted on day 15 after plating. The base layer consisted of 2 mL with a final concentration of culture medium and 0.6% low melting temperature agarose (Lonza). Next, the respective cells were seeded with culture medium containing 0.6% agar to result in a final concentration of was 0.3% agarose with 5 x 10^4^ cells. A further 500 μL culture medium only was added above the congealed middle agar layer on the next day. The colonies were captured at five random fields at 40× magnification with inverted phase microscope (Leica), measured using the Leica software. The scale was set at ImageJ to select colony size more than 0.45 mm.

### 2.5 Proliferation assay

Cell proliferation was measured using a bright field image label‐free high‐content time‐lapse assay system (IncuCyte Zoom system; Essen BioScience) according to the manufacturer's instructions. In brief, for the proliferation assay, equal numbers of cells (1 × 10^3^ cells/well) were seeded on to 96‐well plate in the 200 μL culture medium with supplements or agents, and per cent cell confluence was then continuously measured using the IncuCyte system over a 5‐day period.

### 2.6 Wound healing assay

Each cell line group consists of OPN OE and control (pBABE puro). The initial seeding density was 2 × 10^4^ and 4 × 10^4^ cells/100 μL growth medium in a 96‐well plate (Image Lock plates, Essen Bioscience), respectively, for DLD1 and DKS8 cells, and left to reach fully confluence before wounding with a wound maker (Essen Bioscience). The plates were replaced with the new medium (200 μL) before further incubated and visualized for 60 hours at 37°C in the IncuCyte. Wound width was measured every two hours and images were captured at set time 400× magnification. This experiment was repeated three times.

### 2.7 Transwell migration/invasion assay

Migration and invasion transwell assays were performed using 24‐well transwell inserts (Cell Biolabs, Inc.). The cells were starved for 16‐18 hours prior seeding. The migration plates contained inserts with polycarbonate membrane with 8 mm size pores, while the invasion plated contained ECM‐coated cell culture inserts. Cells (1 × 10^5^) suspended in serum‐free media were added into each insert and 500 μL media consist of 30% FBS was added to the bottom chamber of each well. Cells were left for 48 hours and non‐migratory/non‐invasive cells were removed as much with cotton swabs. The inserts were stained with 400 μL of Cell Stain Solution and incubated at room temperature for 10 minutes. The migratory/invasive cells were photographed with Carl Zeiss Axio Observer with five random fields (10 objectives) per insert. After that, transfer each insert to an empty well, adding 200 μL of Extraction Solution per well, then incubating 10 minutes on an orbital shaker. Transfer 100 μL from each sample to a 96‐well microtitre plate and measure the OD 560 nm in a plate reader. This experiment was repeated three times.

### 2.8 Extraction of clinical and microarray gene expression data from colon cancer patient datasets

Two colon cancer patient datasets available in the Gene Expression Omnibus database were included in this study as previously described.^14^ Microarray gene expression data were retrieved from the data matrices and R scripting was used to extract the expression values of genes of interest and the clinical data from the data matrices. The median expression value was used as a cut‐off point for high and low levels expression for survival analysis. The details of the data extraction method and R script referred to the previous publication.^15^

### 2.9 Connectivity mapping

**Figure 1:**
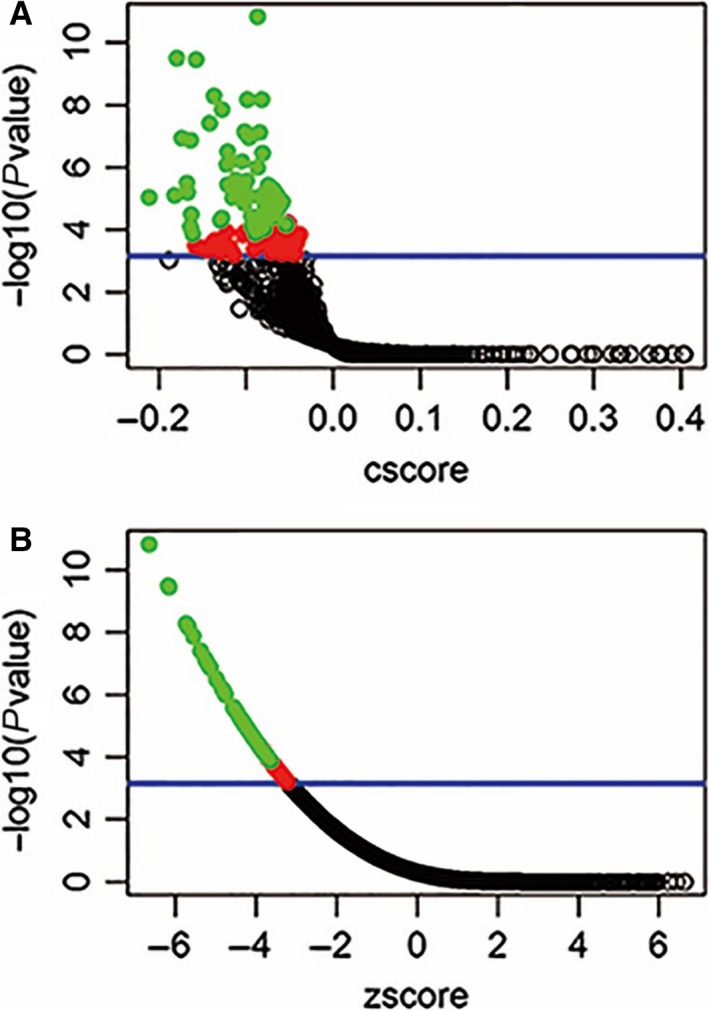
Connectivity mapping of OPN gene signatures. Connectivity mapping shows the results of gene expression using the OPN gene signature as query to sscMap and the QUADrATiC collection of reference profiles for FDA approved drugs. The blue line in the figure represents the *P*‐value threshold (.0007) as described above, where it intersects the vertical axis at −log10(0.0007) = 3.16. The number of significant drugs (above the blue line, red or green spots) is 95, and the number of green spots (drugs with perturbation stability = 1) is 53, which all are listed in Table 1. The full results of connectivity mapping to all 1432 drugs can be found in Table S1

Gene expression connectivity mapping was performed using the algorithms in the framework of Statistically Significant Connection's Map (sscMap)^16^, ^17^ to identify candidate small molecule compounds that may inhibit the expression of genes that are co‐regulated in OPN‐overexpressing colon cancer. OPN and 15 co‐regulated genes (see Section ACKNOWLEDGEMENT below) formed a gene signature for connectivity mapping. The compiled gene signature was then fed to the Java application sscMap as a query signature, and its association with a large collection of reference gene expression profiles were compared. In this study, we used the reference gene expression profiles recently released with the QUADrATiC system,^18^ covering data for over 1000 FDA approved drugs from the LINCS database (https://clue.io/). The gene signature perturbation procedure,^19^ which increases the specificity of the output results, was applied. All the small molecular compounds, that were negatively associated with the OPN Gene Expression Signature, were sorted and ranked by their *P*‐value, perturbation stability and standardized connection score. As we are only interested in inhibitory compounds, one‐tailed *P*‐values were used. To effectively control false positive discoveries, a stringent *P*‐value threshold was set as 1/N = 1/1432 = .0007, where N = 1432 is the number of FDA drugs screened here. Any drugs with a *P*‐value smaller than the set threshold are considered statistically significant (those above the blue line in Figure 1). These criteria control the expected number of falsely significant drugs at 1, as previously described and justified for multiple testing issues.^20^, ^21^ Given the number of significant drugs as 95 in this study, the overall false discovery rate of our results is approximately 1% (1/95). The top 20 small molecules were searched together with colon cancer using the PubMed search engine to identify research articles that have described their effects on the treatment of colon cancer.

## 3 RESULTS

### 3.1 Overexpression of OPN in colon cancer cells with mutant KRAS

**Figure 2:**
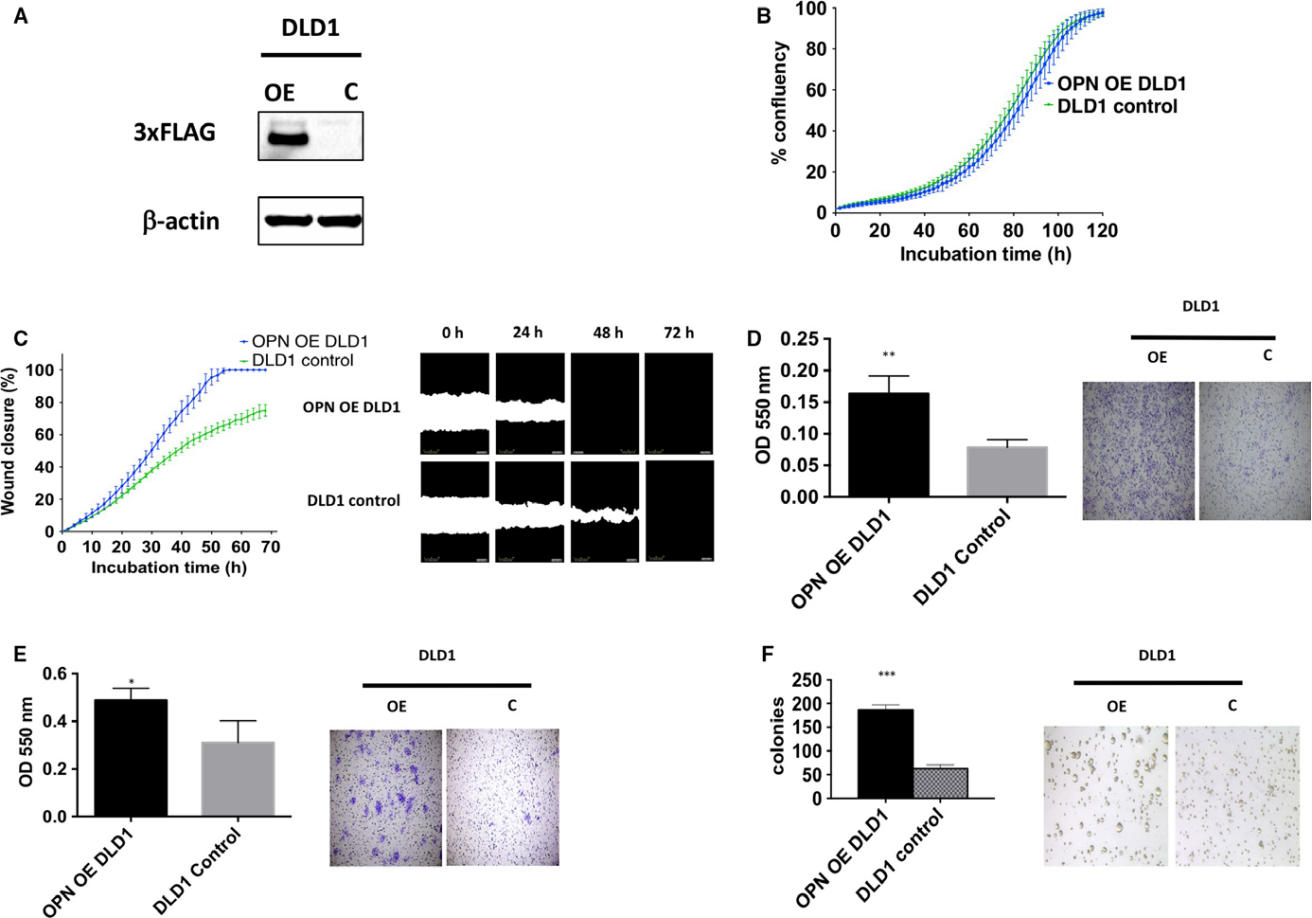
Phenotype changes of OPN overexpression in DLD1. A, Western blot analysis of FLAG‐tag OPN level in OPN OE and control group, β‐actin was used as a control. B, Cell proliferation was recorded for 7 d using the IncuCyte instrument, mean ± SD (error bars, n = 9). C, Cell migration was measured by wound closure over 72 h using the IncuCyte, mean ± SD (error bars, n = 9). D, The migration rates determined and analysed after 48 h with Boyden Chamber assay. Images are representative sections of one well at 20x. E, The invasion rates determined and analysed after 48 h with Boyden Chamber assay. Images are representative sections of one well at 20x. F, Cells were seeded in a 0.6% agar/growth medium layer and its colony formation was measured after 15 d of cultivation. The graphs indicate the mean ± SD, of triplicate wells counted using ImageJ software and the images are representative sections of one well at 40x. “*,” “**” and “***” indicate *P* < .05, *P* < .01 and *P* < .001, respectively, and statistically analysed with T‐test

To evaluate the role of OPN overexpression in colon cancer, FLAG‐tag OPN was overexpressed in DLD1 colon cancer cell lines (Figure 2A). As shown in Figure 2, overexpression of OPN did not significantly increase cell proliferation (Figure 2B). OPN overexpression significantly increased cell migration as demonstrated by wound closure assay (Figure 2C) and transwell migration assay (Figure 2D), and cell invasion as demonstrated by transwell invasion assay (Figure 2E) in DLD1 colon cancer cells. Overexpression of OPN in DLD1 cells also significantly increased its ability to form colony in soft agar assay (Figure 2F).

### 3.2 Overexpression of OPN in colon cancer cells with wild‐type KRAS

**Figure 3:**
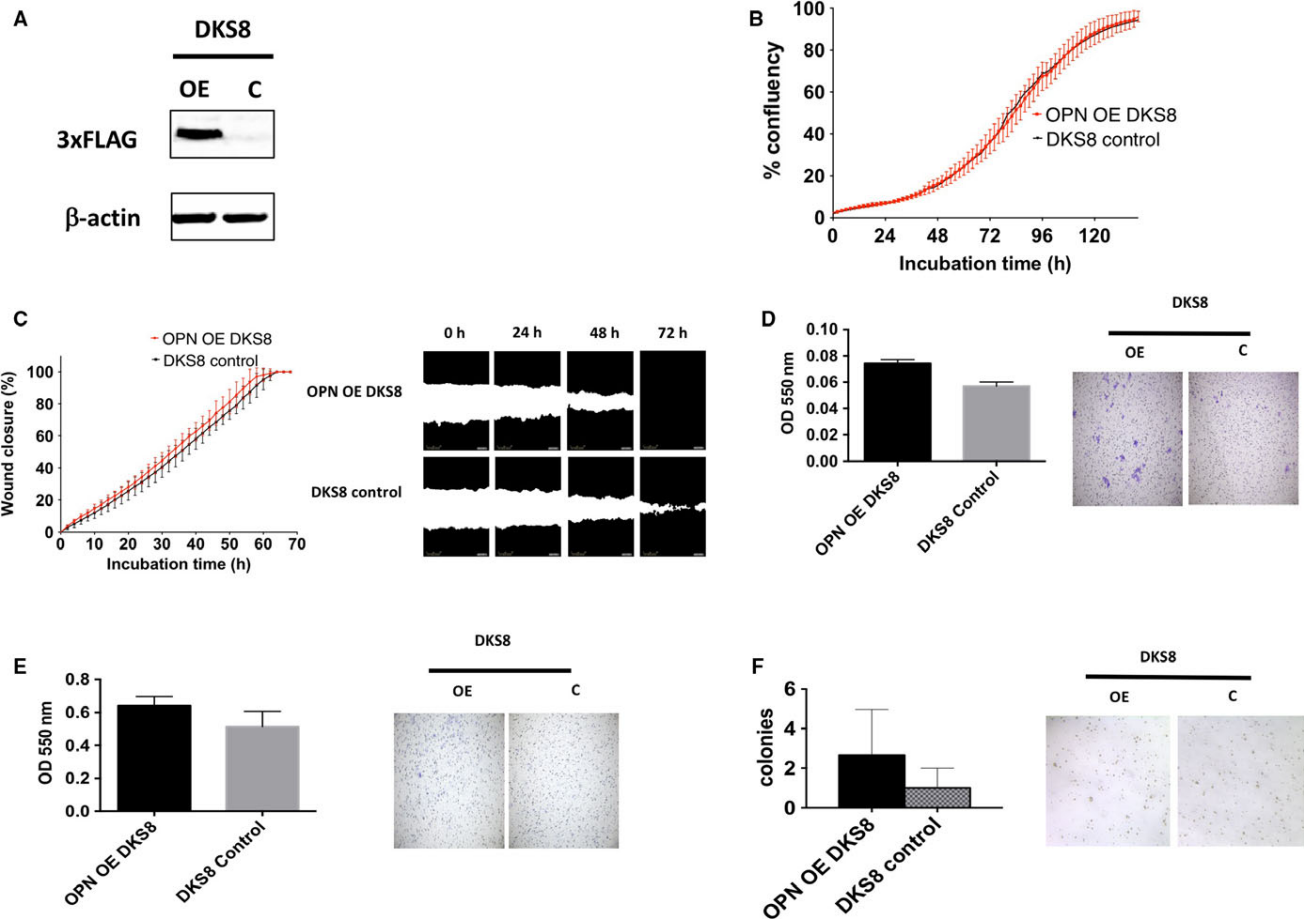
Phenotype changes of OPN overexpression in DKS8. A, Western blot analysis of FLAG‐tag OPN level in OPN OE and control group, β‐actin was used as a control. B, Cell proliferation was recorded for 7 d using the IncuCyte instrument, mean ± SD (error bars, n = 9). C, Cell migration was measured by wound closure over 72 h using the IncuCyte, mean ± SD (error bars, n = 9). D, The migration rates determined and analysed after 48 h with Boyden Chamber assay. Images are representative sections of one well at 20x. E, The invasion rates determined and analysed after 48 h with Boyden Chamber assay. Images are representative sections of one well at 20x. F, Cells were seeded in a 0.6% agar/growth medium layer and its colony formation was measured after 15 d of cultivation. The graphs indicate the mean ± SD, of triplicate wells counted using ImageJ software and the images are representative sections of one well at 40x. “*,” “**” and “***” indicate *P* < .05, *P* < .01 and *P* < .001, respectively, and statistically analysed with *T*‐test

As DLD1 contains mutant *KRAS* while it has an isogenic *KRAS* wild‐type DKS8 generated previously,^22^ FLAG‐tag OPN was overexpressed in DKS8 cells (Figure 3A) and its phenotype was investigated. As shown in Figure 3, overexpression of OPN again did not significantly increase cell proliferation in DKS8 cells (Figure 3B). OPN overexpression did not significantly increase cell migration in wound closure assay (Figure 3C), but slightly and significantly increased cell migration in transwell migration assay (Figure 3D) and cell invasion in transwell invasion assay (Figure 3E). Overexpression of OPN did not significantly increase anchorage‐independent growth of DKS8 cells (Figure 3F).

### 3.3 OPN overexpression enhanced oncogenic signalling dependent on KRAS mutation status

**Figure 4:**
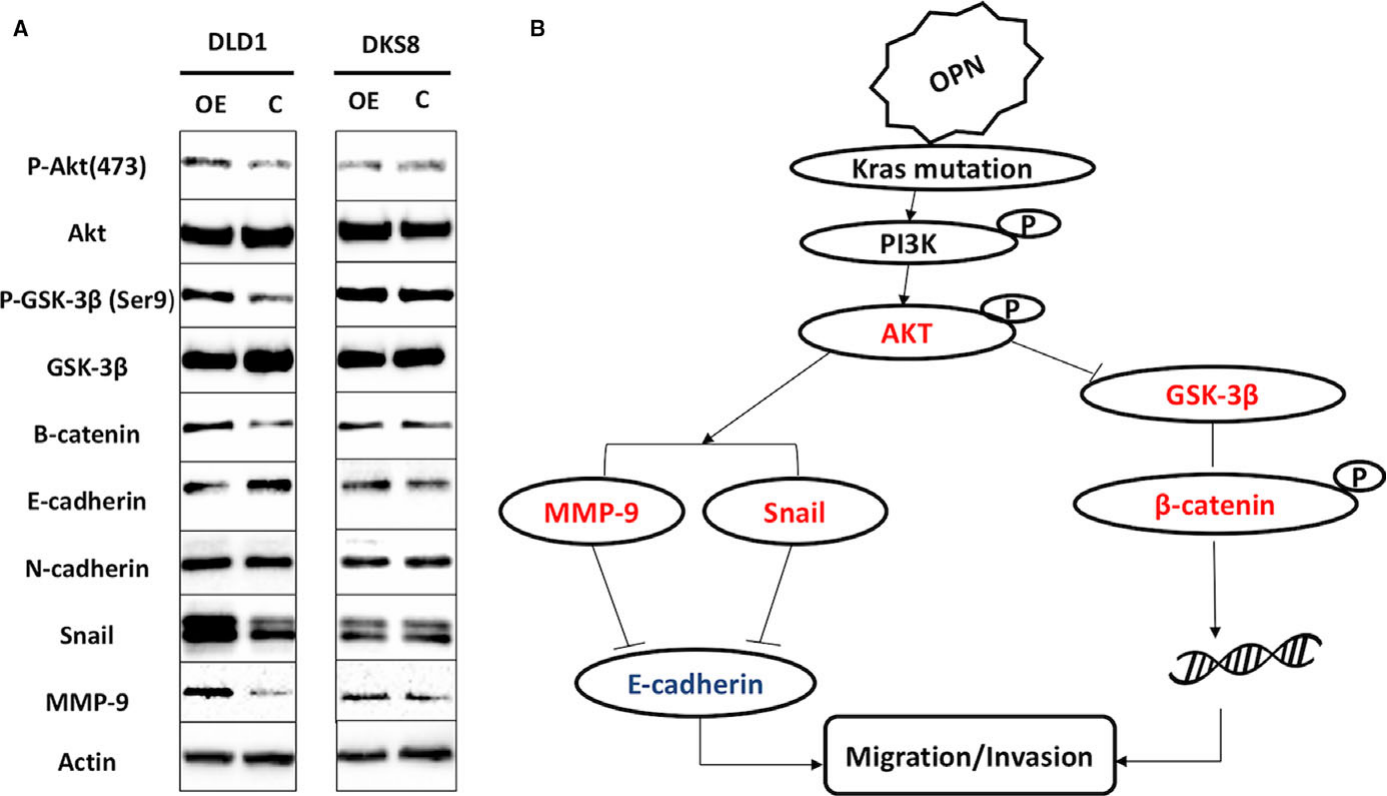
Overexpression of OPN effect on *KRAS* signalling pathway. A, Western blot analysis of OPN OE. B, Predicted signalling pathways involved in OPN overexpression in *KRAS* mutant CRC

As shown in Figure 4, OPN overexpression in DLD1 cells resulted in increased Akt phosphorylation in an activation site and GSK‐3β phosphorylation in an inhibitory site, suggesting OPN may activate PI3K signalling pathway. Besides, we observed an increased in β‐catenin, MMP9 and Snail, and decreased in E‐cadherin, suggesting potential epithelial‐mesenchymal transition by OPN overexpression in DLD1 cells. These changes induced by OPN overexpression were not observed in DKS8 cells, suggesting that OPN may induce the oncogenic signal in *KRAS* mutant dependent manner.

### 3.4 The prognostic significance of OPN overexpression in colon cancer

**Figure 5:**
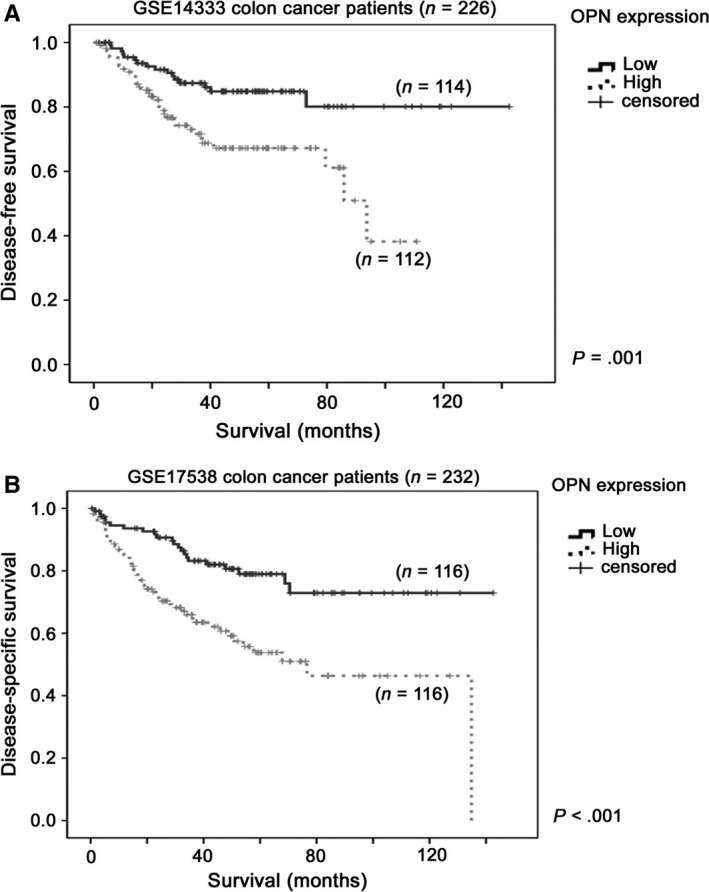
Kaplan‐Meier survival analysis for OPN expression in colon cancer patient cohorts. Kaplan‐Meier survival analysis for OPN expression in A, GSE14333 and B, GSE17538 colon cancer datasets

To investigate whether OPN expression is a prognostic marker in colon cancer, two colon cancer datasets in GEO database previously described^13^, ^14^ with available data on OPN expression, disease‐specific survival were included in this study, namely GSE14333^23^ and GSE17538.^24^ In GSE14333 colon cancer patient cohort with available clinical information (n = 226), we found that patients whose tumour expressed a high‐level expression of OPN had a mean survival time of 74.4 months (95% CI = 64.6‐84.2 months), which was significantly shorter compared to those patients whose tumour expressed a low level of OPN, who had a mean survival time of 120.8 months (95% CI = 110.3‐131.3 months, *P* = .004; Figure 5A). In GSE17538 colon cancer patient cohort with available clinical information (n = 232), we found that patients whose tumour expressed a high‐level expression of OPN had a mean survival time of 78.6 months (95% CI = 66.2‐91.0 months), which was significantly shorter compared to those patients whose tumour expressed a low level of OPN, who had a mean survival time of 113.5 months (95% CI = 102.6‐124.5 months, *P* < .001; Figure 5B). Our results confirm that OPN is a potential prognostic marker in colon cancer patients.

### 3.5 Identification of genes that were associated with OPN overexpression

From these two colon cancer patient datasets, we have divided the patients into two groups in each dataset based on the expression level of OPN in their tumours. We have identified the top 15 genes that were differentially expressed in these two groups consistently in the two datasets. These 15 genes were *CLEC5A, TREM2, OLR1, C5AR1, GPNMB, POSTN, CTSL1, TYROBP, CD163, PRRX1, FCER1G, BCAT1, TREM1, FCGR2C* and *NCF2*. Indeed, from these two datasets, we also found that OPN expression was negative correlated with E‐cadherin expression (GSE14333: *r* = −.355, *P* < .001 and GSE17538: *r* = −.165, *P* = .012) and was positive correlated with MMP9 expression (GSE14333: *r* = .442, *P* < .001 and GSE17538: *r* = .484, *P* < .001), consistent with our findings in vitro in DLD1 cell line.

### 3.6 Identification of small molecules that can reverse OPN gene signature

Using connectivity mapping, OPN and the top 15 OPN co‐regulated genes, we have identified 53 small molecules that could potentially reverse colon cancer OPN gene (*SPP1*) signature passing the perturbation stability test (Table 1) among 1432 candidate drugs. The full results of all 1432 drugs are attached in “Table S1.” The top five small molecules are bendroflumethiazide, calcitriol, benzoic acid, memantine and sibutramine.

**Table 1:** Candidate drugs that may have the potential to reverse the *SPP1* gene signature

Compound	Replicate	*P* value	zscore	Significance_mark	Perturb_stability
BRD‐A80017228__bendroflumethiazide	140	1.51E‐11	−6.645712778	1	1
BRD‐A66229260__calcitriol	27	3.15E‐10	−6.182628333	1	1
BRD‐K53397409__benzoic‐acid	27	3.54E‐10	−6.16412438	1	1
BRD‐A79803969__memantine	53	5.18E‐09	−5.72465667	1	1
BRD‐A23359898__sibutramine	77	6.60E‐09	−5.683522877	1	1
BRD‐K02992638__lamivudine	95	6.74E‐09	−5.679906593	1	1
BRD‐A62434282__goserelin	48	1.37E‐08	−5.556859227	1	1
BRD‐A35258977__mannitol	25	3.85E‐08	−5.374021465	1	1
BRD‐A65013509__oxybutynin	45	7.32E‐08	−5.257119145	1	1
BRD‐K91315211__betahistine	75	7.71E‐08	−5.247492361	1	1
BRD‐A29426959__carbinoxamine	126	1.12E‐07	−5.178125706	1	1
BRD‐A49172652__lansoprazole	45	1.15E‐07	−5.173084601	1	1
BRD‐K94436377__diosmin	12	1.38E‐07	−5.139469425	1	1
BRD‐A88774919__doxycycline	45	3.06E‐07	−4.987137365	1	1
BRD‐K34157611__cimetidine	90	3.40E‐07	−4.967242256	1	1
BRD‐A67097164__ifosfamide	38	6.44E‐07	−4.841514805	1	1
BRD‐A10386413__ascorbic‐acid	26	8.01E‐07	−4.798100724	1	1
BRD‐K44497846__enalapril	45	1.00E‐06	−4.753272058	1	1
BRD‐K45330754__diethylstilbestrol	542	2.49E‐06	−4.56598884	1	1
BRD‐K55930204__phenytoin	45	2.60E‐06	−4.556400029	1	1
BRD‐K71696703__triflusal	12	3.23E‐06	−4.510431872	1	1
BRD‐A88208128__tetracycline	27	3.50E‐06	−4.493420086	1	1
BRD‐K70976396__cefoxitin	56	3.73E‐06	−4.480358699	1	1
BRD‐A16754160__ampicillin	70	5.08E‐06	−4.413784472	1	1
BRD‐A07106394__tryptophan	27	5.72E‐06	−4.387838983	1	1
BRD‐K15502390__nevirapine	81	6.34E‐06	−4.365424658	1	1
BRD‐A34006693__suprofen	11	6.35E‐06	−4.365105537	1	1
BRD‐A55815733__phylloquinone	45	7.02E‐06	−4.3432216	1	1
BRD‐K21520694__sulfacetamide	99	7.25E‐06	−4.336298442	1	1
BRD‐K41524689__paracetamol	11	7.71E‐06	−4.322594942	1	1
BRD‐A24514565__warfarin	108	8.15E‐06	−4.310251978	1	1
BRD‐A90515964__guaifenesin	65	8.47E‐06	−4.301828669	1	1
BRD‐K89348303__ramipril	82	8.75E‐06	−4.294711525	1	1
BRD‐K84091759__candesartan	11	9.11E‐06	−4.285769298	1	1
BRD‐A43286952__ethinyl‐estradiol	27	9.22E‐06	−4.283002437	1	1
BRD‐A02759312__betaxolol	110	1.25E‐05	−4.214835372	1	1
BRD‐K94353609__fluocinolone	88	1.35E‐05	−4.197053722	1	1
BRD‐K65146499__nabumetone	64	1.41E‐05	−4.188246176	1	1
BRD‐K34441861__moexipril	71	1.88E‐05	−4.121806342	1	1
BRD‐K81839095__estrone	372	2.34E‐05	−4.070928448	1	1
BRD‐K44876623__zolpidem	81	2.38E‐05	−4.067188474	1	1
BRD‐M58473998__alimemazine	11	3.17E‐05	−3.999544267	1	1
BRD‐K24844714__fluorouracil	142	3.62E‐05	−3.968351449	1	1
BRD‐K54416256__methimazole	56	4.12E‐05	−3.937566409	1	1
BRD‐K57930253__nitrazepam	37	4.22E‐05	−3.931721025	1	1
BRD‐A74500471__ethambutol	46	4.75E‐05	−3.90293934	1	1
BRD‐K15262564__mupirocin	45	4.90E‐05	−3.895436606	1	1
BRD‐A15131297__benazepril	81	6.84E‐05	−3.81377276	1	1
BRD‐M07438658__lapatinib	67	7.27E‐05	−3.798806938	1	1
BRD‐K82225283__isosorbide	11	7.75E‐05	−3.782771087	1	1
BRD‐A72441487__stiripentol	49	9.22E‐05	−3.739479613	1	1
BRD‐K18895904__olanzapine	48	1.31E‐04	−3.650144317	1	1
BRD‐A20348246__chlormezanone	10	1.33E‐04	−3.646768303	1	1

## 4 DISCUSSION

In this study, we established OPN stable overexpressing cells using *KRAS* mutant/wild‐type isogenic pair of colon cancer cell line to investigate the potential role and mechanism of OPN overexpression in colon cancer progression. Our results suggest that overexpression of OPN promote colon cancer cell migration and invasion, and anchorage‐independent growth significantly in the presence of mutant *KRAS* and to a lesser extent in the wild‐type *KRAS* counterpart. Indeed, OPN overexpression only activates oncogenic signals, including Akt signalling, Snail, β‐catenin and MMP9 in *KRAS* mutant DLD1 cells, but not in *KRAS* wild‐type DKS8 cells, suggesting OPN‐induced oncogenic signalling is dependent on activation of *KRAS*.

We have identified the top 15 genes that were co‐upregulated with OPN in human colon cancer specimens. Based on online literature search, we found that *OLR1, GPNMB, PRRX1 and BCAT1* promote cancer migration and invasion in various types of cancer. Specifically, *OLR1* has been recognized as a cell migration stimulator in breast cancer cells, and potentially up‐regulated by oncogene *TBC1D3* through activation of TNFa/NF‐_K_B pathway.^25^ Non‐metastatic glycoprotein melanoma protein B (GPNMB), also known as osteoactivin (OA) is expressed in verified tumours and represents an emerging target for drug development. For example, GPNMB/OA increases the invasiveness of human metastatic prostate cancer cell lines DU145 and PC3 through MMP2 and MMP9 activities, and also enhances tumorigenesis of human lung cancer and breast cancer^26^, ^27^, ^28^ *PRRX1* promotes epithelial‐mesenchymal transition (EMT) through the Wnt/b‐catenin pathway in gastric cancer and also has been identified as a new EMT inducer in breast and colorectal cancer.^29^, ^30^ Similarly, overexpression of *BCAT1*, a c‐Myc target gene might associate with ovarian cancer progression and induce cell proliferation, migration and invasion in nasopharyngeal carcinoma.^31^, ^32^ Some in vitro trials indicated that suppression of *BCAT1* could significantly decrease tumour proliferation in glioma cell lines.^33^ It has been well known that OPN overexpression has enhanced tumour behaviour, but the potential co‐regulation genes involved in this complex process have not been well identified. In our previous work, we have shown that the expression of OPN and MMP9 were positively correlated in human bladder cancer specimens and bladder cancer cell lines.^12^ In the present study, we found that overexpression of OPN up‐regulated MMP9 while OPN and MMP9 expression levels were positively correlated in two independent colon cancer datasets. Our results demonstrated that MMP9 may be a universal downstream target of OPN in both colon and bladder cancer progression.

In the present study, we found a list of candidate drugs that may have potential to reverse the OPN gene (*SPP1*) signature. On the top of the list, calcitriol is one of the significant candidates that might affect the OPN gene signature. Calcitriol (1,25‐Dihydroxyvitamin D) the most active metabolite of vitamin D, has significant anti‐neoplastic activity in preclinical models. There are some well‐studied mechanisms such as growth inhibition and accumulation in G0‐G1, associated with transcriptional activation of CDK inhibitors p27Kip1 and/or p21Waf1 as well as some other mitogenic signals, induction of apoptosis, and inhibition of invasiveness and angiogenesis have also been reported.^34^ Some reports indicated that calcitriol would enhance radiation sensitivity in colorectal cancer regulated by the epithelial‐mesenchymal transition, for example, in vitro experiment trial of DLD1 and HCT116, 24 hours calcitriol pre‐treatment enhanced the radiation sensitivity by 2.3‐ and 2.6‐fold.^35^ Pre‐clinical and epidemiological studies have promoted the anti‐tumour effect of calcitriol, particularly against colorectal cancer which involved in anti‐proliferation, pro‐differentiation, pro‐apoptosis, anti‐angiogenesis, immune modulation and miR regulation.^36^ A number of genes are recognized to contain functional vitamin D response elements, which also include OPN.^34^, ^37^ Survival from colorectal cancer has been reported to be positively associated with vitamin D status and OPN expression level, respectively.^5^, ^38^ The potential effect of calcitriol on OPN gene signature in colorectal cancer development warrant further investigation.

## CONFLICT OF INTEREST STATEMENT

The authors declare no competing financial interests.

## AUTHORS’ CONTRIBUTIONS

RW, JPCW, PL and XX carried out the experiments, while PL and OT performed the statistical analysis. SDZ, HFY and HFK analysed and interpreted the GEO Dataset. RW and JPCW drafted the manuscript. HFK participated in the design of the study. SS and HFK revised the manuscript. All authors read and approved the final manuscript.
