# Supplementary material for: In vitro and clinical data analysis of Osteopontin as a prognostic indicator in colorectal cancer
Source: J Cell Mol Med. 2018 May 30;22(9):4097–105. doi: 10.1111/jcmm.13686 (PMC6111822; doi:10.1111/jcmm.13686)
Supplement: Supplementary file 1 [file JCMM-22-4097-s001.docx]

**Supplementary Table 1**

| unique ID | set size | SPP1-cogenes-n22.sig.sscmap.tab_  setcscore | SPP1-cogenes-n22.sig.sscmap.tab_  set p value | SPP1-cogenes-n22.sig.sscmap.tab_setzscore=  setcscore/stdev | SPP1-cogenes-n22.sig.sscmap.tab_  significance mark | Perturb_  stability |
| --- | --- | --- | --- | --- | --- | --- |
| BRD-A80017228__bendroflumethiazide__trt_cp | 140 | -0.086714445 | 1.51E-11 | -6.645712778 | 1 | 1 |
| BRD-A66229260__calcitriol__trt_cp | 27 | -0.179819234 | 3.15E-10 | -6.182628333 | 1 | 1 |
| BRD-K53397409__benzoic-acid__trt_cp | 27 | -0.157408334 | 3.54E-10 | -6.16412438 | 1 | 1 |
| BRD-A79803969__memantine__trt_cp | 53 | -0.13649979 | 5.18E-09 | -5.72465667 | 1 | 1 |
| BRD-A23359898__sibutramine__trt_cp | 77 | -0.097872837 | 6.60E-09 | -5.683522877 | 1 | 1 |
| BRD-K02992638__lamivudine__trt_cp | 95 | -0.081422799 | 6.74E-09 | -5.679906593 | 1 | 1 |
| BRD-A62434282__goserelin__trt_cp | 48 | -0.128118905 | 1.37E-08 | -5.556859227 | 1 | 1 |
| BRD-A35258977__mannitol__trt_cp | 25 | -0.14189501 | 3.85E-08 | -5.374021465 | 1 | 1 |
| BRD-A65013509__oxybutynin__trt_cp | 45 | -0.101470643 | 7.32E-08 | -5.257119145 | 1 | 1 |
| BRD-K91315211__betahistine__trt_cp | 75 | -0.084118489 | 7.71E-08 | -5.247492361 | 1 | 1 |
| BRD-A29426959__carbinoxamine__trt_cp | 126 | -0.096478893 | 1.12E-07 | -5.178125706 | 1 | 1 |
| BRD-A49172652__lansoprazole__trt_cp | 45 | -0.173673089 | 1.15E-07 | -5.173084601 | 1 | 1 |
| BRD-K94436377__diosmin__trt_cp | 12 | -0.163951205 | 1.38E-07 | -5.139469425 | 1 | 1 |
| BRD-A88774919__doxycycline__trt_cp | 45 | -0.12144246 | 3.06E-07 | -4.987137365 | 1 | 1 |
| BRD-K34157611__cimetidine__trt_cp | 90 | -0.081113584 | 3.40E-07 | -4.967242256 | 1 | 1 |
| BRD-A67097164__ifosfamide__trt_cp | 38 | -0.104909855 | 6.44E-07 | -4.841514805 | 1 | 1 |
| BRD-A10386413__ascorbic-acid__trt_cp | 26 | -0.121986264 | 8.01E-07 | -4.798100724 | 1 | 1 |
| BRD-K44497846__enalapril__trt_cp | 45 | -0.086459178 | 1.00E-06 | -4.753272058 | 1 | 1 |
| BRD-K45330754__diethylstilbestrol__trt_cp | 542 | -0.11152296 | 2.49E-06 | -4.56598884 | 1 | 1 |
| BRD-K55930204__phenytoin__trt_cp | 45 | -0.099138549 | 2.60E-06 | -4.556400029 | 1 | 1 |
| BRD-K71696703__triflusal__trt_cp | 12 | -0.167753719 | 3.23E-06 | -4.510431872 | 1 | 1 |
| BRD-A88208128__tetracycline__trt_cp | 27 | -0.121133496 | 3.50E-06 | -4.493420086 | 1 | 1 |
| BRD-K70976396__cefoxitin__trt_cp | 56 | -0.074138496 | 3.73E-06 | -4.480358699 | 1 | 1 |
| BRD-A16754160__ampicillin__trt_cp | 70 | -0.068451259 | 5.08E-06 | -4.413784472 | 1 | 1 |
| BRD-A07106394__tryptophan__trt_cp | 27 | -0.108861387 | 5.72E-06 | -4.387838983 | 1 | 1 |
| BRD-K15502390__nevirapine__trt_cp | 81 | -0.076755688 | 6.34E-06 | -4.365424658 | 1 | 1 |
| BRD-A34006693__suprofen__trt_cp | 11 | -0.16678527 | 6.35E-06 | -4.365105537 | 1 | 1 |
| BRD-A55815733__phylloquinone__trt_cp | 45 | -0.098751119 | 7.02E-06 | -4.3432216 | 1 | 1 |
| BRD-K21520694__sulfacetamide__trt_cp | 99 | -0.06492468 | 7.25E-06 | -4.336298442 | 1 | 1 |
| BRD-K41524689__paracetamol__trt_cp | 11 | -0.181673185 | 7.71E-06 | -4.322594942 | 1 | 1 |
| BRD-A24514565__warfarin__trt_cp | 108 | -0.064328683 | 8.15E-06 | -4.310251978 | 1 | 1 |
| BRD-A90515964__guaifenesin__trt_cp | 65 | -0.085102845 | 8.47E-06 | -4.301828669 | 1 | 1 |
| BRD-K89348303__ramipril__trt_cp | 82 | -0.077746433 | 8.75E-06 | -4.294711525 | 1 | 1 |
| BRD-K84091759__candesartan__trt_cp | 11 | -0.211062201 | 9.11E-06 | -4.285769298 | 1 | 1 |
| BRD-A43286952__ethinyl-estradiol__trt_cp | 27 | -0.115347681 | 9.22E-06 | -4.283002437 | 1 | 1 |
| BRD-A02759312__betaxolol__trt_cp | 110 | -0.058274061 | 1.25E-05 | -4.214835372 | 1 | 1 |
| BRD-K94353609__fluocinolone__trt_cp | 88 | -0.065871937 | 1.35E-05 | -4.197053722 | 1 | 1 |
| BRD-K65146499__nabumetone__trt_cp | 64 | -0.101509434 | 1.41E-05 | -4.188246176 | 1 | 1 |
| BRD-K34441861__moexipril__trt_cp | 71 | -0.062173061 | 1.88E-05 | -4.121806342 | 1 | 1 |
| BRD-K81839095__estrone__trt_cp | 372 | -0.079157368 | 2.34E-05 | -4.070928448 | 1 | 1 |
| BRD-K44876623__zolpidem__trt_cp | 81 | -0.064538164 | 2.38E-05 | -4.067188474 | 1 | 1 |
| BRD-M58473998__alimemazine__trt_cp | 11 | -0.162906273 | 3.17E-05 | -3.999544267 | 1 | 1 |
| BRD-K24844714__fluorouracil__trt_cp | 142 | -0.094079108 | 3.62E-05 | -3.968351449 | 1 | 1 |
| BRD-K54416256__methimazole__trt_cp | 56 | -0.071506558 | 4.12E-05 | -3.937566409 | 1 | 1 |
| BRD-K57930253__nitrazepam__trt_cp | 37 | -0.127688869 | 4.22E-05 | -3.931721025 | 1 | 1 |
| BRD-A74500471__ethambutol__trt_cp | 46 | -0.083531376 | 4.75E-05 | -3.90293934 | 1 | 1 |
| BRD-K15262564__mupirocin__trt_cp | 45 | -0.129245595 | 4.90E-05 | -3.895436606 | 1 | 1 |
| BRD-A15131297__benazepril__trt_cp | 81 | -0.0541519 | 6.84E-05 | -3.81377276 | 1 | 1 |
| BRD-M07438658__lapatinib__trt_cp | 67 | -0.093612996 | 7.27E-05 | -3.798806938 | 1 | 1 |
| BRD-K82225283__isosorbide__trt_cp | 11 | -0.164251627 | 7.75E-05 | -3.782771087 | 1 | 1 |
| BRD-A72441487__stiripentol__trt_cp | 49 | -0.079672337 | 9.22E-05 | -3.739479613 | 1 | 1 |
| BRD-K18895904__olanzapine__trt_cp | 48 | -0.088809484 | 1.31E-04 | -3.650144317 | 1 | 1 |
| BRD-A20348246__chlormezanone__trt_cp | 10 | -0.161482238 | 1.33E-04 | -3.646768303 | 1 | 1 |
| BRD-K60511616__pravastatin__trt_cp | 150 | -0.051178112 | 6.05E-05 | -3.844024167 | 1 | 0.956521739 |
| BRD-K57391913__iloprost__trt_cp | 100 | -0.098404756 | 1.11E-04 | -3.692972695 | 1 | 0.956521739 |
| BRD-A47564106__tetrabenazine__trt_cp | 24 | -0.102901473 | 1.34E-04 | -3.644175254 | 1 | 0.956521739 |
| BRD-A24228527__ofloxacin__trt_cp | 54 | -0.102805244 | 1.49E-04 | -3.616387075 | 1 | 0.956521739 |
| BRD-A90490067__fulvestrant__trt_cp | 220 | -0.057244197 | 2.21E-04 | -3.513767328 | 1 | 0.956521739 |
| BRD-K04046242__equilin__trt_cp | 351 | -0.084185865 | 2.86E-04 | -3.44424101 | 1 | 0.956521739 |
| BRD-K72166146__dihydroergotamine__trt_cp | 85 | -0.051703283 | 2.93E-04 | -3.438055013 | 1 | 0.956521739 |
| BRD-K91938660__memantine__trt_cp | 36 | -0.090534231 | 6.53E-05 | -3.825137946 | 1 | 0.913043478 |
| BRD-K88429204__pyrimethamine__trt_cp | 163 | -0.066445258 | 8.07E-05 | -3.772859605 | 1 | 0.913043478 |
| BRD-K71350836__butalbital__trt_cp | 12 | -0.122296536 | 1.23E-04 | -3.66649015 | 1 | 0.913043478 |
| BRD-K45252063__clofibrate__trt_cp | 93 | -0.056388936 | 1.75E-04 | -3.575131172 | 1 | 0.913043478 |
| BRD-K21317336__phentolamine__trt_cp | 45 | -0.072118648 | 2.35E-04 | -3.49738055 | 1 | 0.913043478 |
| BRD-K97530723__melatonin__trt_cp | 120 | -0.08904267 | 1.23E-04 | -3.666593507 | 1 | 0.869565217 |
| BRD-K56614220__clofazimine__trt_cp | 44 | -0.076918496 | 2.48E-04 | -3.482851374 | 1 | 0.869565217 |
| BRD-K20141153__atomoxetine__trt_cp | 101 | -0.041926502 | 2.69E-04 | -3.461561021 | 1 | 0.869565217 |
| BRD-K03012696__cefdinir__trt_cp | 11 | -0.158151166 | 3.35E-04 | -3.401479117 | 1 | 0.869565217 |
| BRD-K08273968__griseofulvin__trt_cp | 54 | -0.090649673 | 4.37E-04 | -3.328120454 | 1 | 0.869565217 |
| BRD-K50938287__sumatriptan__trt_cp | 49 | -0.083701227 | 2.08E-04 | -3.529293187 | 1 | 0.826086957 |
| BRD-K12437696__aztreonam__trt_cp | 10 | -0.150151226 | 3.79E-04 | -3.36735582 | 1 | 0.826086957 |
| BRD-K67043667__altretamine__trt_cp | 139 | -0.038083356 | 1.45E-04 | -3.623857539 | 1 | 0.782608696 |
| BRD-K57545991__enalapril__trt_cp | 181 | -0.064734205 | 4.47E-04 | -3.321968388 | 1 | 0.739130435 |
| BRD-K74763371__bosentan__trt_cp | 55 | -0.06571231 | 2.22E-04 | -3.512448473 | 1 | 0.695652174 |
| BRD-A41304429__practolol__trt_cp | 91 | -0.051676236 | 8.00E-04 | -3.155781314 | 0 | 0.695652174 |
| BRD-K96253961__metaraminol__trt_cp | 10 | -0.13531884 | 2.17E-04 | -3.518947146 | 1 | 0.652173913 |
| BRD-K96319534__phentermine__trt_cp | 83 | -0.048852221 | 2.24E-04 | -3.509601233 | 1 | 0.652173913 |
| BRD-K32830106__guanfacine__trt_cp | 83 | -0.052713873 | 3.49E-04 | -3.390274252 | 1 | 0.652173913 |
| BRD-K37949327__etidronic-acid__trt_cp | 11 | -0.12862263 | 5.31E-04 | -3.273320874 | 1 | 0.652173913 |
| BRD-A92670106__tocainide__trt_cp | 100 | -0.043178101 | 6.03E-04 | -3.23726688 | 1 | 0.652173913 |
| BRD-K54462405__doxepin__trt_cp | 110 | -0.057598024 | 3.89E-04 | -3.36043245 | 1 | 0.608695652 |
| BRD-K28667793__pyrazinamide__trt_cp | 97 | -0.057475987 | 5.02E-04 | -3.289375831 | 1 | 0.608695652 |
| BRD-K48367671__febuxostat__trt_cp | 65 | -0.058249195 | 5.15E-04 | -3.282037617 | 1 | 0.608695652 |
| BRD-K89708791__rifaximin__trt_cp | 37 | -0.118244023 | 3.61E-04 | -3.380705767 | 1 | 0.565217391 |
| BRD-K89046952__ciclacillin__trt_cp | 67 | -0.054972551 | 5.12E-04 | -3.283796263 | 1 | 0.565217391 |
| BRD-K45542189__diethylcarbamazine__trt_cp | 96 | -0.044801059 | 5.27E-04 | -3.275866285 | 1 | 0.565217391 |
| BRD-K10852020__tolcapone__trt_cp | 69 | -0.070501749 | 5.31E-04 | -3.273574608 | 1 | 0.565217391 |
| BRD-K35941380__methysergide__trt_cp | 82 | -0.04647991 | 6.01E-04 | -3.238567479 | 1 | 0.565217391 |
| BRD-A03061970__alizapride__trt_cp | 12 | -0.113436652 | 6.46E-04 | -3.217867392 | 1 | 0.565217391 |
| BRD-M56587789__ibutilide__trt_cp | 12 | -0.137655931 | 2.50E-04 | -3.481025057 | 1 | 0.52173913 |
| BRD-K99912150__rifampicin__trt_cp | 12 | -0.131468027 | 2.77E-04 | -3.452888385 | 1 | 0.52173913 |
| BRD-A20239487__atenolol__trt_cp | 45 | -0.053366894 | 0.00117006 | -3.043283277 | 0 | 0.52173913 |
| BRD-K00835182__dexamethasone__trt_cp | 10 | -0.138014153 | 3.11E-04 | -3.421977066 | 1 | 0.434782609 |
| BRD-K60369935__ribavirin__trt_cp | 11 | -0.143432848 | 4.59E-04 | -3.314468893 | 1 | 0.434782609 |
| BRD-A43974575__tranylcypromine__trt_cp | 52 | -0.061202969 | 4.61E-04 | -3.313010548 | 1 | 0.434782609 |
| BRD-A87606379__nadolol__trt_cp | 138 | -0.041143805 | 8.04E-04 | -3.154539868 | 0 | 0.434782609 |
| BRD-A37959677__estrone__trt_cp | 53 | -0.071487842 | 8.35E-04 | -3.143268148 | 0 | 0.391304348 |
| BRD-A25576662__streptozotocin__trt_cp | 44 | -0.068714255 | 8.70E-04 | -3.13131898 | 0 | 0.391304348 |
| BRD-K09859624__methantheline__trt_cp | 74 | -0.057697637 | 5.82E-04 | -3.247631984 | 1 | 0.347826087 |
| BRD-A33168282__sotalol__trt_cp | 101 | -0.047652441 | 7.79E-04 | -3.163557359 | 0 | 0.260869565 |
| BRD-A24643465__homoharringtonine__trt_cp | 39 | -0.18857458 | 9.71E-04 | -3.098941364 | 0 | 0.260869565 |
| BRD-A52282606__lacidipine__trt_cp | 36 | -0.088284791 | 0.001555177 | -2.956615869 | 0 | 0.260869565 |
| BRD-K47278471__diphenhydramine__trt_cp | 47 | -0.06176485 | 0.001628158 | -2.942445083 | 0 | 0.217391304 |
| BRD-K79759585__sildenafil__trt_cp | 126 | -0.072523343 | 7.65E-04 | -3.169016065 | 0 | 0.173913043 |
| BRD-A83237092__fulvestrant__trt_cp | 431 | -0.067440736 | 8.94E-04 | -3.123493151 | 0 | 0.173913043 |
| BRD-K11905747__spectinomycin__trt_cp | 56 | -0.057115871 | 0.001040729 | -3.078356386 | 0 | 0.173913043 |
| BRD-A82307304__atorvastatin__trt_cp | 45 | -0.078167818 | 0.001352461 | -2.999422169 | 0 | 0.173913043 |
| BRD-K90333595__phentolamine__trt_cp | 73 | -0.047238628 | 0.001367206 | -2.996117423 | 0 | 0.173913043 |
| BRD-A76229785__lymecycline__trt_cp | 11 | -0.12148092 | 0.001421512 | -2.984219969 | 0 | 0.173913043 |
| BRD-K19416115__sitagliptin__trt_cp | 63 | -0.080998055 | 8.07E-04 | -3.153306731 | 0 | 0.130434783 |
| BRD-A34751532__homosalate__trt_cp | 74 | -0.055384859 | 9.29E-04 | -3.112139484 | 0 | 0.130434783 |
| BRD-K89595132__chlorphenamine__trt_cp | 85 | -0.040703819 | 0.001044384 | -3.077311569 | 0 | 0.130434783 |
| BRD-A30815329__felodipine__trt_cp | 75 | -0.049460427 | 0.001229649 | -3.028304966 | 0 | 0.130434783 |
| BRD-K81128206__edrophonium__trt_cp | 55 | -0.047890849 | 0.001251263 | -3.023035812 | 0 | 0.130434783 |
| BRD-K55889443__rifapentine__trt_cp | 37 | -0.08792172 | 0.001440598 | -2.980136792 | 0 | 0.130434783 |
| BRD-A02180903__betamethasone__trt_cp | 82 | -0.065701078 | 0.001802772 | -2.910756966 | 0 | 0.130434783 |
| BRD-K17041687__diflorasone__trt_cp | 12 | -0.100001276 | 0.001831797 | -2.905762516 | 0 | 0.130434783 |
| BRD-K85307935__novobiocin__trt_cp | 11 | -0.122364919 | 0.002008451 | -2.876831361 | 0 | 0.130434783 |
| BRD-K57569181__pentoxifylline__trt_cp | 218 | -0.0532668 | 8.53E-04 | -3.137255092 | 0 | 0.086956522 |
| BRD-K68432770__ampicillin__trt_cp | 10 | -0.126558157 | 9.39E-04 | -3.109005157 | 0 | 0.086956522 |
| BRD-K73437736__azlocillin__trt_cp | 11 | -0.133000948 | 0.001276357 | -3.017021959 | 0 | 0.086956522 |
| BRD-K85925969__zalcitabine__trt_cp | 48 | -0.052410619 | 0.003251798 | -2.721247972 | 0 | 0.086956522 |
| BRD-K44094599__tacrolimus__trt_cp | 37 | -0.09089336 | 0.006239931 | -2.498277046 | 0 | 0.086956522 |
| BRD-K40831222__latamoxef__trt_cp | 11 | -0.129872093 | 0.001665206 | -2.935471557 | 0 | 0.043478261 |
| BRD-K63242551__cefadroxil__trt_cp | 12 | -0.101103135 | 0.00174662 | -2.920630374 | 0 | 0.043478261 |
| BRD-A67438293__treprostinil__trt_cp | 66 | -0.055757755 | 0.001863852 | -2.900329587 | 0 | 0.043478261 |
| BRD-K54316499__tolterodine__trt_cp | 35 | -0.078692196 | 0.002171263 | -2.852145265 | 0 | 0.043478261 |
| BRD-K93645900__tadalafil__trt_cp | 114 | -0.039756629 | 0.002251077 | -2.840651061 | 0 | 0.043478261 |
| BRD-A93269281__clopidogrel__trt_cp | 24 | -0.079704747 | 0.002279998 | -2.836577149 | 0 | 0.043478261 |
| BRD-A18043272__phensuximide__trt_cp | 63 | -0.068460621 | 0.002422147 | -2.817209374 | 0 | 0.043478261 |
| BRD-A09472452__flecainide__trt_cp | 139 | -0.041017148 | 0.002617183 | -2.792245303 | 0 | 0.043478261 |
| BRD-K95237249__probenecid__trt_cp | 163 | -0.041572989 | 0.0028062 | -2.769606848 | 0 | 0.043478261 |
| BRD-K76810206__nicergoline__trt_cp | 83 | -0.050689191 | 0.002868464 | -2.76245035 | 0 | 0.043478261 |
| BRD-K74501079__azithromycin__trt_cp | 57 | -0.058846981 | 0.003557267 | -2.691435137 | 0 | 0.043478261 |
| BRD-A90490067__fulvestrant__trt_poscon | 533 | -0.042345781 | 0.004052553 | -2.647659698 | 0 | 0.043478261 |
| BRD-K38310698__troleandomycin__trt_cp | 10 | -0.121668998 | 0.005412637 | -2.54828902 | 0 | 0.043478261 |
| BRD-A79672927__tropicamide__trt_cp | 152 | -0.030869729 | 9.57E-04 | -3.103288978 | 0 | 0 |
| BRD-A46335897__promethazine__trt_cp | 174 | -0.032192359 | 0.00149125 | -2.969535911 | 0 | 0 |
| BRD-A08200164__melphalan__trt_cp | 27 | -0.090882618 | 0.0016133 | -2.945282325 | 0 | 0 |
| BRD-K38003476__clocortolone__trt_cp | 65 | -0.051909571 | 0.001808829 | -2.909708702 | 0 | 0 |
| BRD-M30251550__penbutolol__trt_cp | 11 | -0.117317777 | 0.001979874 | -2.881350629 | 0 | 0 |
| BRD-A15493168__tetracycline__trt_cp | 90 | -0.045113689 | 0.002269637 | -2.838031189 | 0 | 0 |
| BRD-K68264559__brimonidine__trt_cp | 110 | -0.030977737 | 0.002322614 | -2.830658583 | 0 | 0 |
| BRD-K18673820__folic-acid__trt_cp | 12 | -0.106837838 | 0.002425919 | -2.816709611 | 0 | 0 |
| BRD-K82255054__propofol__trt_cp | 73 | -0.038616057 | 0.002500789 | -2.806932061 | 0 | 0 |
| BRD-K79501723__cyclizine__trt_cp | 12 | -0.10969634 | 0.002897047 | -2.759212008 | 0 | 0 |
| BRD-K63460351__toremifene__trt_cp | 11 | -0.127014852 | 0.003318871 | -2.714492394 | 0 | 0 |
| BRD-K97045029__pranlukast__trt_cp | 12 | -0.107404382 | 0.003520507 | -2.694898206 | 0 | 0 |
| BRD-K05977355__fluconazole__trt_cp | 107 | -0.03245864 | 0.003565123 | -2.690699133 | 0 | 0 |
| BRD-K39339537__epirizole__trt_cp | 264 | -0.060023048 | 0.003637045 | -2.684028508 | 0 | 0 |
| BRD-A29260609__acebutolol__trt_cp | 53 | -0.047611565 | 0.003651594 | -2.682693525 | 0 | 0 |
| BRD-A71816415__pravastatin__trt_cp | 62 | -0.044821723 | 0.003747227 | -2.674035387 | 0 | 0 |
| BRD-K99712306__yohimbine__trt_cp | 12 | -0.096436679 | 0.003792846 | -2.669974932 | 0 | 0 |
| BRD-K71451869__halcinonide__trt_cp | 12 | -0.10925301 | 0.003810634 | -2.668403474 | 0 | 0 |
| BRD-A80908310__cloperastine__trt_cp | 12 | -0.095862052 | 0.003840156 | -2.665809841 | 0 | 0 |
| BRD-K91699951__benzonatate__trt_cp | 234 | -0.053420579 | 0.003861015 | -2.663988101 | 0 | 0 |
| BRD-K54978501__pivmecillinam__trt_cp | 11 | -0.119517889 | 0.003897753 | -2.660800802 | 0 | 0 |
| BRD-K67919934__spironolactone__trt_cp | 10 | -0.109213442 | 0.004230841 | -2.633070322 | 0 | 0 |
| BRD-A16665823__butoconazole__trt_cp | 63 | -0.050464368 | 0.004404129 | -2.619407775 | 0 | 0 |
| BRD-A45543382__metrizamide__trt_cp | 65 | -0.044567911 | 0.004658959 | -2.600164108 | 0 | 0 |
| BRD-K19533706__tranilast__trt_cp | 76 | -0.043397366 | 0.004795107 | -2.590264759 | 0 | 0 |
| BRD-A98725278__methysergide__trt_cp | 71 | -0.040110881 | 0.004815026 | -2.58883753 | 0 | 0 |
| BRD-A01826957__xanthinol__trt_cp | 49 | -0.053894531 | 0.004844485 | -2.586736332 | 0 | 0 |
| BRD-A80869209__ticarcillin__trt_cp | 11 | -0.120255963 | 0.005138183 | -2.566388484 | 0 | 0 |
| BRD-K13240564__crotamiton__trt_cp | 11 | -0.10363421 | 0.005188046 | -2.563037266 | 0 | 0 |
| BRD-K52930707__rescinnamine__trt_cp | 68 | -0.043187419 | 0.005314765 | -2.554647765 | 0 | 0 |
| BRD-K20920669__cromoglicic-acid__trt_cp | 55 | -0.052647905 | 0.005490703 | -2.543289931 | 0 | 0 |
| BRD-K28346421__rifapentine__trt_cp | 46 | -0.05507532 | 0.005498417 | -2.542799434 | 0 | 0 |
| BRD-A91699651__chloroquine__trt_cp | 401 | -0.022866631 | 0.005593487 | -2.536803483 | 0 | 0 |
| BRD-A70514680__articaine__trt_cp | 63 | -0.043597153 | 0.005598584 | -2.536484584 | 0 | 0 |
| BRD-A54845972__dihydroergotamine__trt_cp | 53 | -0.050895115 | 0.00563677 | -2.534103589 | 0 | 0 |
| BRD-K82846253__repaglinide__trt_cp | 98 | -0.051944843 | 0.005735551 | -2.52801019 | 0 | 0 |
| BRD-A84282119__rimantadine__trt_cp | 12 | -0.101369304 | 0.006047953 | -2.50933399 | 0 | 0 |
| BRD-K04804440__ciprofloxacin__trt_cp | 10 | -0.103939836 | 0.00606023 | -2.508617643 | 0 | 0 |
| BRD-K14965640__ibuprofen__trt_cp | 73 | -0.036224836 | 0.00635245 | -2.491935826 | 0 | 0 |
| BRD-K13356952__methazolamide__trt_cp | 55 | -0.055863461 | 0.006539729 | -2.481598455 | 0 | 0 |
| BRD-K68507560__dicycloverine__trt_cp | 95 | -0.03659374 | 0.006571528 | -2.479869288 | 0 | 0 |
| BRD-A31800922__procyclidine__trt_cp | 56 | -0.052035685 | 0.006677579 | -2.474155425 | 0 | 0 |
| BRD-K81144366__moxisylyte__trt_cp | 152 | -0.089143162 | 0.006767113 | -2.4693936 | 0 | 0 |
| BRD-K90885812__propantheline__trt_cp | 56 | -0.061798044 | 0.006960776 | -2.459281223 | 0 | 0 |
| BRD-A42571354__cetirizine__trt_cp | 121 | -0.03061669 | 0.007283773 | -2.442955195 | 0 | 0 |
| BRD-K05395900__nicotine__trt_cp | 97 | -0.035135803 | 0.007445332 | -2.43502737 | 0 | 0 |
| BRD-K95739795__tetrabenazine__trt_cp | 77 | -0.048367735 | 0.007585796 | -2.428256907 | 0 | 0 |
| BRD-K09631521__thiotepa__trt_cp | 83 | -0.058560949 | 0.007725816 | -2.421616869 | 0 | 0 |
| BRD-K51095933__estropipate__trt_cp | 89 | -0.040512081 | 0.007739627 | -2.42096767 | 0 | 0 |
| BRD-K88366685__trimetazidine__trt_cp | 12 | -0.092480573 | 0.008035416 | -2.407302972 | 0 | 0 |
| BRD-A16444946__acarbose__trt_cp | 73 | -0.034937151 | 0.008060623 | -2.406158999 | 0 | 0 |
| BRD-A82892199__alpha-tocopherol__trt_cp | 107 | -0.067288078 | 0.008127626 | -2.403133514 | 0 | 0 |
| BRD-K69470111__montelukast__trt_cp | 37 | -0.064608208 | 0.008172279 | -2.401129329 | 0 | 0 |
| BRD-A95602221__celiprolol__trt_cp | 12 | -0.094789039 | 0.008438433 | -2.389379331 | 0 | 0 |
| BRD-A84493640__atovaquone__trt_cp | 53 | -0.051529916 | 0.008640724 | -2.380664413 | 0 | 0 |
| BRD-K75089421__procainamide__trt_cp | 118 | -0.03117678 | 0.008970912 | -2.366816528 | 0 | 0 |
| BRD-K08924299__palonosetron__trt_cp | 49 | -0.057801471 | 0.008997851 | -2.365706565 | 0 | 0 |
| BRD-A17448384__beclometasone__trt_cp | 49 | -0.062663291 | 0.009090495 | -2.361911315 | 0 | 0 |
| BRD-A69636825__diltiazem__trt_cp | 83 | -0.044011808 | 0.009316863 | -2.352778583 | 0 | 0 |
| BRD-K16606819__trimethadione__trt_cp | 12 | -0.096944935 | 0.009561607 | -2.34312034 | 0 | 0 |
| BRD-K92657060__zaleplon__trt_cp | 12 | -0.09089403 | 0.009645153 | -2.339872866 | 0 | 0 |
| BRD-A67605442__tetrahydrobiopterin__trt_cp | 45 | -0.052492191 | 0.009649568 | -2.339701916 | 0 | 0 |
| BRD-K93034159__cladribine__trt_cp | 105 | -0.066495549 | 0.00976912 | -2.335099171 | 0 | 0 |
| BRD-K18910433__estradiol__trt_cp | 992 | -0.02702448 | 0.010194521 | -2.31911056 | 0 | 0 |
| BRD-K66404838__nalbuphine__trt_cp | 63 | -0.041872067 | 0.0108589 | -2.295267485 | 0 | 0 |
| BRD-K62363391__dapsone__trt_cp | 53 | -0.054063781 | 0.010951819 | -2.292034767 | 0 | 0 |
| BRD-A93255169__thalidomide__trt_cp | 330 | -0.023116438 | 0.01134222 | -2.278707827 | 0 | 0 |
| BRD-K93524252__sulfamerazine__trt_cp | 12 | -0.082014404 | 0.011546585 | -2.271889744 | 0 | 0 |
| BRD-K46862739__metyrapone__trt_cp | 63 | -0.046423835 | 0.011775124 | -2.264388222 | 0 | 0 |
| BRD-A16700644__isoxsuprine__trt_cp | 50 | -0.046685121 | 0.012051923 | -2.25546995 | 0 | 0 |
| BRD-A30977374__nifedipine__trt_cp | 37 | -0.063726359 | 0.012302367 | -2.247552597 | 0 | 0 |
| BRD-K46435977__valaciclovir__trt_cp | 49 | -0.053920746 | 0.012474051 | -2.242205385 | 0 | 0 |
| BRD-K52662033__lidocaine__trt_cp | 110 | -0.036248836 | 0.012588331 | -2.238681262 | 0 | 0 |
| BRD-K24616672__procaine__trt_cp | 154 | -0.073519532 | 0.012672801 | -2.236094195 | 0 | 0 |
| BRD-A17883755__lenalidomide__trt_cp | 171 | -0.028960192 | 0.012681516 | -2.235828128 | 0 | 0 |
| BRD-K07208025__trimethoprim__trt_cp | 10 | -0.087322864 | 0.012955315 | -2.227548624 | 0 | 0 |
| BRD-K31920458__mestranol__trt_cp | 101 | -0.033074137 | 0.013485687 | -2.211931857 | 0 | 0 |
| BRD-A60197193__amisulpride__trt_cp | 102 | -0.035116755 | 0.014181807 | -2.19222015 | 0 | 0 |
| BRD-K61496577__flumetasone__trt_cp | 64 | -0.036628789 | 0.014333402 | -2.188038408 | 0 | 0 |
| BRD-A41555725__chlortetracycline__trt_cp | 53 | -0.042042567 | 0.014365368 | -2.187161513 | 0 | 0 |
| BRD-K92778217__mefenamic-acid__trt_cp | 198 | -0.053235097 | 0.014381334 | -2.186724155 | 0 | 0 |
| BRD-K72816382__cinalukast__trt_cp | 64 | -0.044977737 | 0.014387247 | -2.18656228 | 0 | 0 |
| BRD-K59037100__oxybenzone__trt_cp | 77 | -0.033819252 | 0.015061051 | -2.168481101 | 0 | 0 |
| BRD-A22380646__pantoprazole__trt_cp | 99 | -0.033425202 | 0.015265235 | -2.163139264 | 0 | 0 |
| BRD-A25234499__aminoglutethimide__trt_cp | 139 | -0.024125803 | 0.015266587 | -2.163104103 | 0 | 0 |
| BRD-K67080878__milrinone__trt_cp | 118 | -0.030766425 | 0.01533608 | -2.161300158 | 0 | 0 |
| BRD-K92049597__triamterene__trt_cp | 307 | -0.046248544 | 0.015526086 | -2.156403496 | 0 | 0 |
| BRD-A35519318__benidipine__trt_cp | 36 | -0.058205497 | 0.015678674 | -2.152508221 | 0 | 0 |
| BRD-A13982089__ephedrine__trt_cp | 24 | -0.064205205 | 0.017035497 | -2.119230469 | 0 | 0 |
| BRD-K25650355__physostigmine__trt_cp | 83 | -0.035529159 | 0.017455299 | -2.109393883 | 0 | 0 |
| BRD-A97701745__pindolol__trt_cp | 90 | -0.038613542 | 0.017618781 | -2.105617843 | 0 | 0 |
| BRD-A23226398__estradiol__trt_cp | 62 | -0.036471067 | 0.017747613 | -2.102663142 | 0 | 0 |
| BRD-K54790157__trioxsalen__trt_cp | 74 | -0.033969763 | 0.017820025 | -2.101010436 | 0 | 0 |
| BRD-K13800121__parecoxib__trt_cp | 37 | -0.052074709 | 0.017962766 | -2.097769265 | 0 | 0 |
| BRD-K24559712__norethindrone__trt_cp | 10 | -0.09043346 | 0.01812696 | -2.094068046 | 0 | 0 |
| BRD-K82357231__desloratadine__trt_cp | 48 | -0.053747737 | 0.018938058 | -2.076192873 | 0 | 0 |
| BRD-A65739223__ramipril__trt_cp | 52 | -0.036324638 | 0.019016871 | -2.074490902 | 0 | 0 |
| BRD-K73323637__levobunolol__trt_cp | 11 | -0.079503334 | 0.019085579 | -2.073012056 | 0 | 0 |
| BRD-A14792000__valsartan__trt_cp | 45 | -0.04046154 | 0.01959364 | -2.062214903 | 0 | 0 |
| BRD-A07395371__esmolol__trt_cp | 47 | -0.046374258 | 0.019624204 | -2.061572996 | 0 | 0 |
| BRD-K11433652__aspirin__trt_cp | 44 | -0.041216212 | 0.020106108 | -2.051562325 | 0 | 0 |
| BRD-K87202646__isoniazid__trt_cp | 27 | -0.050872119 | 0.020736601 | -2.038767857 | 0 | 0 |
| BRD-A56892734__esomeprazole__trt_cp | 36 | -0.051751906 | 0.020895515 | -2.035595102 | 0 | 0 |
| BRD-K02265150__amoxapine__trt_cp | 173 | -0.030850099 | 0.021145041 | -2.030654229 | 0 | 0 |
| BRD-K73109821__diazoxide__trt_cp | 111 | -0.025920111 | 0.021293637 | -2.027735275 | 0 | 0 |
| BRD-K35520305__dacarbazine__trt_cp | 12 | -0.08393902 | 0.021707133 | -2.019702408 | 0 | 0 |
| BRD-A74667430__etodolac__trt_cp | 94 | -0.027662062 | 0.021973408 | -2.014597733 | 0 | 0 |
| BRD-K79062806__nicergoline__trt_cp | 10 | -0.074182758 | 0.022380722 | -2.006889364 | 0 | 0 |
| BRD-K59058747__acetylcysteine__trt_cp | 118 | -0.045257022 | 0.022505994 | -2.004542425 | 0 | 0 |
| BRD-A31312900__montelukast__trt_cp | 42 | -0.047532035 | 0.022506489 | -2.004533166 | 0 | 0 |
| BRD-A48180038__trihexyphenidyl__trt_cp | 12 | -0.074140909 | 0.022608018 | -2.002639136 | 0 | 0 |
| BRD-K70301876__escitalopram__trt_cp | 132 | -0.024849719 | 0.023014312 | -1.995130732 | 0 | 0 |
| BRD-A54880345__etomidate__trt_cp | 126 | -0.024673809 | 0.023232341 | -1.991147422 | 0 | 0 |
| BRD-A05186015__bupropion__trt_cp | 166 | -0.025287531 | 0.023243214 | -1.99094961 | 0 | 0 |
| BRD-K36862742__hydroflumethiazide__trt_cp | 195 | -0.046430362 | 0.024042019 | -1.976624969 | 0 | 0 |
| BRD-A75368507__demeclocycline__trt_cp | 44 | -0.04208262 | 0.024666508 | -1.965702246 | 0 | 0 |
| BRD-K69688083__mestinon__trt_cp | 101 | -0.032788561 | 0.025202315 | -1.956514035 | 0 | 0 |
| BRD-K75868704__iopamidol__trt_cp | 11 | -0.08560463 | 0.025614572 | -1.949555306 | 0 | 0 |
| BRD-K46211610__tolazoline__trt_cp | 108 | -0.024877874 | 0.025933617 | -1.944234053 | 0 | 0 |
| BRD-K10671814__sulfaphenazole__trt_cp | 154 | -0.060554532 | 0.02657478 | -1.933703772 | 0 | 0 |
| BRD-A50310035__misoprostol__trt_cp | 11 | -0.084791922 | 0.026762959 | -1.930653496 | 0 | 0 |
| BRD-A67981824__cefotaxime__trt_cp | 45 | -0.054609217 | 0.02705285 | -1.925989373 | 0 | 0 |
| BRD-K03243820__trifluridine__trt_cp | 35 | -0.051569542 | 0.027056329 | -1.925933654 | 0 | 0 |
| BRD-K95309561__dienestrol__trt_cp | 373 | -0.029393822 | 0.027088277 | -1.925422256 | 0 | 0 |
| BRD-K67511046__naloxone__trt_cp | 108 | -0.023969269 | 0.027291683 | -1.922177979 | 0 | 0 |
| BRD-A07780951__orciprenaline__trt_cp | 89 | -0.032590018 | 0.027888188 | -1.912778801 | 0 | 0 |
| BRD-U73308409__vemurafenib__trt_cp | 144 | -0.084508946 | 0.028129493 | -1.909024076 | 0 | 0 |
| BRD-K98769987__flumazenil__trt_cp | 104 | -0.033524853 | 0.028428366 | -1.904410587 | 0 | 0 |
| BRD-A66559694__naltrexone__trt_cp | 123 | -0.034229823 | 0.02855589 | -1.902454358 | 0 | 0 |
| BRD-A84465106__nisoldipine__trt_cp | 12 | -0.08678212 | 0.028656022 | -1.900923421 | 0 | 0 |
| BRD-K53342282__cortisol__trt_cp | 11 | -0.079146782 | 0.029424371 | -1.889321657 | 0 | 0 |
| BRD-K97799481__theophylline__trt_cp | 100 | -0.028636149 | 0.029614917 | -1.886483472 | 0 | 0 |
| BRD-A62035778__scopolamine__trt_cp | 63 | -0.034820129 | 0.030072427 | -1.879730222 | 0 | 0 |
| BRD-A11990600__lorazepam__trt_cp | 37 | -0.04578417 | 0.030089688 | -1.87947711 | 0 | 0 |
| BRD-K96354014__nifedipine__trt_cp | 244 | -0.030300165 | 0.030464947 | -1.874003853 | 0 | 0 |
| BRD-K31534764__fluvoxamine__trt_cp | 37 | -0.052176985 | 0.030566171 | -1.872537046 | 0 | 0 |
| BRD-K42348709__cyclobenzaprine__trt_cp | 12 | -0.068810309 | 0.030624184 | -1.871698202 | 0 | 0 |
| BRD-K29905972__axitinib__trt_cp | 62 | -0.038590817 | 0.030907141 | -1.867625558 | 0 | 0 |
| BRD-K54770957__etoricoxib__trt_cp | 12 | -0.077818083 | 0.031811566 | -1.854811624 | 0 | 0 |
| BRD-K23566484__nilutamide__trt_cp | 72 | -0.030535502 | 0.031826802 | -1.854598346 | 0 | 0 |
| BRD-K83636919__entacapone__trt_cp | 45 | -0.041413824 | 0.031887838 | -1.853744823 | 0 | 0 |
| BRD-K55034111__pefloxacin__trt_cp | 48 | -0.041167473 | 0.032177498 | -1.849712511 | 0 | 0 |
| BRD-A72075775__iopromide__trt_cp | 11 | -0.0734996 | 0.032276699 | -1.848338447 | 0 | 0 |
| BRD-K36927236__glibenclamide__trt_cp | 332 | -0.017966242 | 0.03266528 | -1.842989392 | 0 | 0 |
| BRD-K04993501__cefixime__trt_cp | 47 | -0.03213945 | 0.033273542 | -1.834720702 | 0 | 0 |
| BRD-A19661776__mianserin__trt_cp | 73 | -0.027494643 | 0.033426955 | -1.832654894 | 0 | 0 |
| BRD-A37780065__triamcinolone__trt_cp | 51 | -0.050446814 | 0.033511304 | -1.831522394 | 0 | 0 |
| BRD-K44353683__nateglinide__trt_cp | 36 | -0.044703522 | 0.033699086 | -1.82900959 | 0 | 0 |
| BRD-K76674262__homoharringtonine__trt_cp | 182 | -0.107477331 | 0.034364652 | -1.820195066 | 0 | 0 |
| BRD-A80151636__bromocriptine__trt_cp | 83 | -0.033564169 | 0.034424738 | -1.819406233 | 0 | 0 |
| BRD-A84134924__pancuronium__trt_cp | 37 | -0.038849202 | 0.03465368 | -1.816410925 | 0 | 0 |
| BRD-K30682080__nafcilline__trt_cp | 11 | -0.078974122 | 0.036384502 | -1.794276771 | 0 | 0 |
| BRD-K66876909__linezolid__trt_cp | 111 | -0.029418844 | 0.0366544 | -1.790903385 | 0 | 0 |
| BRD-K24125544__tetrabenazine__trt_cp | 24 | -0.052688445 | 0.036865474 | -1.788279355 | 0 | 0 |
| BRD-K14036801__roxithromycin__trt_cp | 11 | -0.069322904 | 0.03781594 | -1.776613337 | 0 | 0 |
| BRD-K91601245__mercaptopurine__trt_cp | 70 | -0.036253853 | 0.038510668 | -1.768236729 | 0 | 0 |
| BRD-K76634210__idoxuridine__trt_cp | 10 | -0.079411741 | 0.038614966 | -1.766989831 | 0 | 0 |
| BRD-A70182876__azapropazone__trt_cp | 11 | -0.085740901 | 0.039424893 | -1.757399217 | 0 | 0 |
| BRD-K49669424__bretylium__trt_cp | 11 | -0.081062099 | 0.041003721 | -1.739155345 | 0 | 0 |
| BRD-K62310379__fluticasone__trt_cp | 109 | -0.030125455 | 0.041185876 | -1.737087358 | 0 | 0 |
| BRD-K20075662__betazole__trt_cp | 153 | -0.059159527 | 0.04131063 | -1.735675318 | 0 | 0 |
| BRD-A42153648__formestane__trt_cp | 11 | -0.071433531 | 0.041849405 | -1.729616537 | 0 | 0 |
| BRD-A00827783__dyphylline__trt_cp | 154 | -0.063060175 | 0.042538609 | -1.721957494 | 0 | 0 |
| BRD-K88809146__tranylcypromine__trt_cp | 33 | -0.037673468 | 0.042987143 | -1.717026737 | 0 | 0 |
| BRD-A79981887__midodrine__trt_cp | 182 | -0.016602038 | 0.043321469 | -1.713378453 | 0 | 0 |
| BRD-A95939040__sertaconazole__trt_cp | 81 | -0.030650509 | 0.043509331 | -1.711338392 | 0 | 0 |
| BRD-A84174393__meloxicam__trt_cp | 128 | -0.028781436 | 0.045497688 | -1.690170313 | 0 | 0 |
| BRD-A65280694__molindone__trt_cp | 109 | -0.022178288 | 0.04565926 | -1.688483158 | 0 | 0 |
| BRD-K59197931__naproxen__trt_cp | 48 | -0.03624555 | 0.045977802 | -1.685170896 | 0 | 0 |
| BRD-A48261811__argatroban__trt_cp | 49 | -0.031719062 | 0.046548121 | -1.679286386 | 0 | 0 |
| BRD-K23933163__cloxacillin__trt_cp | 10 | -0.069422156 | 0.046816741 | -1.676534821 | 0 | 0 |
| BRD-K89125793__tinidazole__trt_cp | 61 | -0.031465992 | 0.046902674 | -1.675657246 | 0 | 0 |
| BRD-K13926615__vardenafil__trt_cp | 111 | -0.021383198 | 0.047050812 | -1.674147447 | 0 | 0 |
| BRD-K80267133__aminosalicylic-acid__trt_cp | 11 | -0.072544495 | 0.047213802 | -1.672490677 | 0 | 0 |
| BRD-K60770992__pergolide__trt_cp | 130 | -0.025596699 | 0.047231292 | -1.672313168 | 0 | 0 |
| BRD-K66788707__fludarabine__trt_cp | 99 | -0.03402292 | 0.048597484 | -1.658607208 | 0 | 0 |
| BRD-K07612980__sparfloxacin__trt_cp | 11 | -0.070874615 | 0.048680226 | -1.657787066 | 0 | 0 |
| BRD-K72093121__vidarabine__trt_cp | 57 | -0.028945031 | 0.048725585 | -1.657337941 | 0 | 0 |
| BRD-A46179541__doxapram__trt_cp | 49 | -0.036634021 | 0.048726801 | -1.657325908 | 0 | 0 |
| BRD-K01095011__finasteride__trt_cp | 98 | -0.019953167 | 0.049141379 | -1.653236414 | 0 | 0 |
| BRD-K69743918__estrone__trt_cp | 11 | -0.067137644 | 0.049235266 | -1.652314121 | 0 | 0 |
| BRD-A50684349__fenoldopam__trt_cp | 103 | -0.02307719 | 0.049936584 | -1.645468818 | 0 | 0 |
| BRD-A36217750__sulfinpyrazone__trt_cp | 106 | -0.021057687 | 0.050194373 | -1.642971911 | 0 | 0 |
| BRD-K92731339__perindopril__trt_cp | 83 | -0.02799775 | 0.050264693 | -1.64229257 | 0 | 0 |
| BRD-K21450440__benzthiazide__trt_cp | 92 | -0.025911637 | 0.050374049 | -1.641237623 | 0 | 0 |
| BRD-K95921201__reserpine__trt_cp | 61 | -0.046621837 | 0.050858174 | -1.636589127 | 0 | 0 |
| BRD-K75641298__metoclopramide__trt_cp | 76 | -0.024170271 | 0.05087705 | -1.636408603 | 0 | 0 |
| BRD-K56558538__ambroxol__trt_cp | 90 | -0.027430511 | 0.051289066 | -1.632481317 | 0 | 0 |
| BRD-A18605525__hexetidine__trt_cp | 12 | -0.067525502 | 0.051339257 | -1.632004611 | 0 | 0 |
| BRD-K70131229__glucosamine__trt_cp | 23 | -0.039189037 | 0.052062369 | -1.625177524 | 0 | 0 |
| BRD-K96390176__calcipotriol__trt_cp | 12 | -0.070949697 | 0.052811984 | -1.61817927 | 0 | 0 |
| BRD-K50859149__sulfafurazole__trt_cp | 159 | -0.019217528 | 0.054407922 | -1.603538373 | 0 | 0 |
| BRD-K57222227__indometacin__trt_cp | 98 | -0.024538913 | 0.055038398 | -1.597848063 | 0 | 0 |
| BRD-A32161980__carbetocin__trt_cp | 63 | -0.038760367 | 0.055101883 | -1.597277939 | 0 | 0 |
| BRD-K26619122__thiocolchicoside__trt_cp | 12 | -0.060689281 | 0.055570244 | -1.593087832 | 0 | 0 |
| BRD-A50157456__terbutaline__trt_cp | 45 | -0.042226352 | 0.056336166 | -1.586295329 | 0 | 0 |
| BRD-A20119038__mecamylamine__trt_cp | 11 | -0.066641015 | 0.05645288 | -1.585266666 | 0 | 0 |
| BRD-A55272860__sulpiride__trt_cp | 10 | -0.05661105 | 0.059204254 | -1.561488612 | 0 | 0 |
| BRD-K48195008__ethinylestradiol__trt_cp | 37 | -0.038030965 | 0.059211814 | -1.561424485 | 0 | 0 |
| BRD-A09056319__alfuzosin__trt_cp | 127 | -0.020109393 | 0.059473426 | -1.55920929 | 0 | 0 |
| BRD-K83896451__glutamine__trt_cp | 106 | -0.045632382 | 0.060279078 | -1.552435074 | 0 | 0 |
| BRD-A17411484__carprofen__trt_cp | 74 | -0.023035164 | 0.062600188 | -1.533306411 | 0 | 0 |
| BRD-K99447003__enalaprilat__trt_cp | 37 | -0.042170888 | 0.062889419 | -1.530961785 | 0 | 0 |
| BRD-A28940325__trichlormethiazide__trt_cp | 11 | -0.061761056 | 0.063299688 | -1.527650341 | 0 | 0 |
| BRD-A37288617__amfepramone__trt_cp | 10 | -0.069182094 | 0.064102291 | -1.521220223 | 0 | 0 |
| BRD-A02990301__lofexidine__trt_cp | 60 | -0.023692644 | 0.065337083 | -1.511448716 | 0 | 0 |
| BRD-K96786677__oxacillin__trt_cp | 12 | -0.056891106 | 0.066846771 | -1.499694557 | 0 | 0 |
| BRD-K55301415__abiraterone__trt_cp | 45 | -0.02760259 | 0.068343043 | -1.488245792 | 0 | 0 |
| BRD-A54596827__solifenacin__trt_cp | 53 | -0.028964797 | 0.068744395 | -1.485207789 | 0 | 0 |
| BRD-K43164539__cholic-acid__trt_cp | 90 | -0.025690761 | 0.069423497 | -1.480098377 | 0 | 0 |
| BRD-K08806317__timolol__trt_cp | 119 | -0.018484907 | 0.069565512 | -1.479034766 | 0 | 0 |
| BRD-K92984783__melperone__trt_cp | 107 | -0.022925525 | 0.07024304 | -1.473983332 | 0 | 0 |
| BRD-K55395145__pemetrexed__trt_cp | 165 | -0.019453213 | 0.071497852 | -1.464725939 | 0 | 0 |
| BRD-A95869247__indapamide__trt_cp | 151 | -0.016124106 | 0.072080178 | -1.460472153 | 0 | 0 |
| BRD-K17016787__estriol__trt_cp | 355 | -0.020791127 | 0.073170642 | -1.452576823 | 0 | 0 |
| BRD-A94543220__bifonazole__trt_cp | 37 | -0.036330299 | 0.074057139 | -1.446224377 | 0 | 0 |
| BRD-K43887077__dopamine__trt_cp | 92 | -0.021760729 | 0.074543421 | -1.442764428 | 0 | 0 |
| BRD-K68132782__terbinafine__trt_cp | 82 | -0.025566334 | 0.075456094 | -1.436316895 | 0 | 0 |
| BRD-A87387433__cefpodoxime__trt_cp | 36 | -0.035333454 | 0.075599668 | -1.435308049 | 0 | 0 |
| BRD-K57875380__asenapine__trt_cp | 10 | -0.060927369 | 0.075936465 | -1.432947193 | 0 | 0 |
| BRD-K77987382__mebendazole__trt_poscon | 167 | -0.050983989 | 0.077293711 | -1.423513254 | 0 | 0 |
| BRD-K37130656__rivaroxaban__trt_cp | 64 | -0.027275886 | 0.07737201 | -1.422972869 | 0 | 0 |
| BRD-K67261995__adipiodone__trt_cp | 82 | -0.019803646 | 0.077809268 | -1.419962714 | 0 | 0 |
| BRD-K72853990__bromocriptine__trt_cp | 10 | -0.060630942 | 0.078020628 | -1.418512281 | 0 | 0 |
| BRD-A64977602__mirtazapine__trt_cp | 130 | -0.017321984 | 0.078277542 | -1.416753245 | 0 | 0 |
| BRD-K70358946__aripiprazole__trt_cp | 47 | -0.034242581 | 0.078597443 | -1.414569059 | 0 | 0 |
| BRD-K92193792__nizatidine__trt_cp | 11 | -0.073364443 | 0.07896417 | -1.412073435 | 0 | 0 |
| BRD-K42260897__ezetimibe__trt_cp | 37 | -0.033238223 | 0.07957506 | -1.407935668 | 0 | 0 |
| BRD-K76747609__beclometasone__trt_cp | 11 | -0.051507849 | 0.07984758 | -1.40609755 | 0 | 0 |
| BRD-K47635719__dexamethasone__trt_cp | 78 | -0.024057984 | 0.081013405 | -1.398287301 | 0 | 0 |
| BRD-M40783228__mesna__trt_cp | 55 | -0.025625479 | 0.082541242 | -1.388179199 | 0 | 0 |
| BRD-K61192372__capecitabine__trt_cp | 63 | -0.026313195 | 0.082745869 | -1.386836124 | 0 | 0 |
| BRD-K50691590__bortezomib__trt_cp | 108 | -0.056342422 | 0.085245778 | -1.370626054 | 0 | 0 |
| BRD-K96659869__spaglumic-acid__trt_cp | 11 | -0.055853213 | 0.086447632 | -1.362959535 | 0 | 0 |
| BRD-K33226500__indinavir__trt_cp | 36 | -0.030653213 | 0.08706582 | -1.359047178 | 0 | 0 |
| BRD-K50388907__fenofibrate__trt_cp | 75 | -0.024022165 | 0.088736577 | -1.348576123 | 0 | 0 |
| BRD-K23984367__sorafenib__trt_cp | 231 | -0.036307582 | 0.089759045 | -1.342240314 | 0 | 0 |
| BRD-A91555231__norepinephrine__trt_cp | 45 | -0.037766296 | 0.091331262 | -1.33260177 | 0 | 0 |
| BRD-A76019558__ketoconazole__trt_cp | 44 | -0.034377794 | 0.094402771 | -1.314120841 | 0 | 0 |
| BRD-K89839824__raltitrexed__trt_cp | 112 | -0.025977263 | 0.09493105 | -1.310987165 | 0 | 0 |
| BRD-A77291778__cyclopentolate__trt_cp | 94 | -0.017279551 | 0.096954419 | -1.299102258 | 0 | 0 |
| BRD-K54262262__monobenzone__trt_cp | 11 | -0.048788643 | 0.097445713 | -1.296244028 | 0 | 0 |
| BRD-K20168442__vecuronium__trt_cp | 49 | -0.025339627 | 0.097737145 | -1.294553534 | 0 | 0 |
| BRD-A34578416__ambrisentan__trt_cp | 11 | -0.051080841 | 0.099932374 | -1.281936995 | 0 | 0 |
| BRD-A62428732__hydroxyzine__trt_cp | 10 | -0.047071671 | 0.100172549 | -1.28056899 | 0 | 0 |
| BRD-K29653726__topiramate__trt_cp | 48 | -0.031778401 | 0.100174004 | -1.280560711 | 0 | 0 |
| BRD-K93568044__hydrocortisone__trt_cp | 45 | -0.032450281 | 0.10089333 | -1.276477827 | 0 | 0 |
| BRD-K57033106__tripelennamine__trt_cp | 99 | -0.018789521 | 0.101004244 | -1.275850174 | 0 | 0 |
| BRD-K92830582__propranolol__trt_cp | 11 | -0.050046275 | 0.101489033 | -1.273112664 | 0 | 0 |
| BRD-A91008255__bepridil__trt_cp | 94 | -0.022355514 | 0.101665799 | -1.272116873 | 0 | 0 |
| BRD-K20338176__cefaclor__trt_cp | 58 | -0.021979981 | 0.102840044 | -1.265533664 | 0 | 0 |
| BRD-A18620900__estriol__trt_cp | 53 | -0.026719634 | 0.105709129 | -1.249675097 | 0 | 0 |
| BRD-A30717181__triamcinolone__trt_cp | 52 | -0.027461494 | 0.106030923 | -1.247915929 | 0 | 0 |
| BRD-K81418486__vorinostat__trt_cp | 1632 | -0.04104112 | 0.106254027 | -1.24669854 | 0 | 0 |
| BRD-K02404261__caffeine__trt_cp | 116 | -0.017815021 | 0.110010659 | -1.226471437 | 0 | 0 |
| BRD-K54314721__zolmitriptan__trt_cp | 112 | -0.015123532 | 0.110813312 | -1.222214228 | 0 | 0 |
| BRD-K49404994__levetiracetam__trt_cp | 49 | -0.02494671 | 0.112646596 | -1.212572802 | 0 | 0 |
| BRD-K54264597__canrenoic-acid__trt_cp | 10 | -0.044876191 | 0.113054552 | -1.210442597 | 0 | 0 |
| BRD-K90699611__acitretin__trt_cp | 12 | -0.048997869 | 0.114183775 | -1.204574644 | 0 | 0 |
| BRD-K82109576__vincristine__trt_cp | 36 | -0.042626762 | 0.114878018 | -1.200987539 | 0 | 0 |
| BRD-A17655518__ibuprofen__trt_cp | 94 | -0.016145352 | 0.115428965 | -1.19815179 | 0 | 0 |
| BRD-K14349461__pyridoxine__trt_cp | 12 | -0.04717528 | 0.115546415 | -1.197548514 | 0 | 0 |
| BRD-A24191444__ifenprodil__trt_cp | 47 | -0.021877962 | 0.116534397 | -1.192490956 | 0 | 0 |
| BRD-K12513978__fenbufen__trt_cp | 93 | -0.014905604 | 0.11731101 | -1.188536727 | 0 | 0 |
| BRD-K67174588__toremifene__trt_cp | 288 | -0.026303013 | 0.118092248 | -1.184577606 | 0 | 0 |
| BRD-K48722833__iloperidone__trt_cp | 64 | -0.023376983 | 0.118208959 | -1.183987733 | 0 | 0 |
| BRD-K00532621__midazolam__trt_cp | 37 | -0.031309216 | 0.119179855 | -1.179096605 | 0 | 0 |
| BRD-A10188456__dexamethasone__trt_cp | 85 | -0.021111216 | 0.119407412 | -1.177954306 | 0 | 0 |
| BRD-K04218075__clomifene__trt_cp | 206 | -0.022095253 | 0.119999391 | -1.174989837 | 0 | 0 |
| BRD-K18910433__estradiol__trt_poscon | 8 | -0.061462301 | 0.121766354 | -1.166202189 | 0 | 0 |
| BRD-K29530284__amlexanox__trt_cp | 37 | -0.032539787 | 0.124818277 | -1.151232608 | 0 | 0 |
| BRD-A10070317__propranolol__trt_cp | 44 | -0.030722628 | 0.125234997 | -1.149208557 | 0 | 0 |
| BRD-A31158127__calcipotriol__trt_cp | 27 | -0.040873371 | 0.125463964 | -1.14809844 | 0 | 0 |
| BRD-A83081521__finasteride__trt_cp | 63 | -0.019778909 | 0.125836755 | -1.146294036 | 0 | 0 |
| BRD-K40367460__chlorpromazine__trt_cp | 24 | -0.031105152 | 0.126716089 | -1.142052547 | 0 | 0 |
| BRD-A22256192__terazosin__trt_cp | 95 | -0.017347693 | 0.127648296 | -1.13757835 | 0 | 0 |
| BRD-M00539986__formoterol__trt_cp | 75 | -0.020672106 | 0.128679381 | -1.132655953 | 0 | 0 |
| BRD-M02190957__orciprenaline__trt_cp | 12 | -0.044078349 | 0.128813153 | -1.132019334 | 0 | 0 |
| BRD-M35773784__atropine__trt_cp | 12 | -0.043131444 | 0.130927938 | -1.122015439 | 0 | 0 |
| BRD-K33811357__dopamine__trt_cp | 24 | -0.031217814 | 0.132676767 | -1.113826715 | 0 | 0 |
| BRD-K28307902__flutamide__trt_cp | 369 | -0.016269029 | 0.133459314 | -1.110186597 | 0 | 0 |
| BRD-K26521938__dinoprostone__trt_cp | 95 | -0.021505627 | 0.13448143 | -1.105454128 | 0 | 0 |
| BRD-K38436528__imipramine__trt_cp | 288 | -0.016613878 | 0.13769567 | -1.090730833 | 0 | 0 |
| BRD-K06878038__deferiprone__trt_cp | 36 | -0.029085304 | 0.13883111 | -1.085585949 | 0 | 0 |
| BRD-A20958854__dopamine__trt_cp | 24 | -0.03036753 | 0.139196443 | -1.083936653 | 0 | 0 |
| BRD-K75699339__rizatriptan__trt_cp | 119 | -0.013774509 | 0.14193062 | -1.07168567 | 0 | 0 |
| BRD-K62736196__guanabenz__trt_cp | 83 | -0.017590696 | 0.143310029 | -1.065565596 | 0 | 0 |
| BRD-K48168960__propylthiouracil__trt_cp | 100 | -0.016461581 | 0.146035052 | -1.053591236 | 0 | 0 |
| BRD-K13044802__ciclopirox__trt_cp | 12 | -0.054032689 | 0.150029206 | -1.036308137 | 0 | 0 |
| BRD-K51471001__demecarium__trt_cp | 12 | -0.038432653 | 0.150618407 | -1.033784724 | 0 | 0 |
| BRD-K46523383__pramocaine__trt_cp | 12 | -0.04480376 | 0.150829297 | -1.032883132 | 0 | 0 |
| BRD-K95773607__darifenacin__trt_cp | 11 | -0.040047416 | 0.151908071 | -1.028284241 | 0 | 0 |
| BRD-K31374463__vecuronium__trt_cp | 11 | -0.047606945 | 0.153160989 | -1.022970112 | 0 | 0 |
| BRD-K53226837__cycloserine__trt_cp | 10 | -0.041136695 | 0.156226469 | -1.010088401 | 0 | 0 |
| BRD-A93291428__oxymetholone__trt_cp | 27 | -0.028176633 | 0.159287353 | -0.997391108 | 0 | 0 |
| BRD-M53001068__aminophylline__trt_cp | 11 | -0.036865928 | 0.161828899 | -0.986969076 | 0 | 0 |
| BRD-K55250441__sulfadoxine__trt_cp | 11 | -0.040128177 | 0.161947456 | -0.986485531 | 0 | 0 |
| BRD-A68723818__brompheniramine__trt_cp | 119 | -0.01374016 | 0.163963416 | -0.978298255 | 0 | 0 |
| BRD-K45071273__tetracaine__trt_cp | 12 | -0.037799056 | 0.167427231 | -0.964381962 | 0 | 0 |
| BRD-K98174813__chlorzoxazone__trt_cp | 10 | -0.038909663 | 0.16885818 | -0.958687178 | 0 | 0 |
| BRD-K49807096__benazepril__trt_cp | 49 | -0.021072549 | 0.168890509 | -0.958558874 | 0 | 0 |
| BRD-K03319035__maprotiline__trt_cp | 63 | -0.022752582 | 0.169201204 | -0.95732665 | 0 | 0 |
| BRD-A02006392__nitrendipine__trt_cp | 288 | -0.009588861 | 0.169540755 | -0.955981639 | 0 | 0 |
| BRD-K39983086__loteprednol__trt_cp | 37 | -0.028673492 | 0.171752036 | -0.947264387 | 0 | 0 |
| BRD-K39981244__norepinephrine__trt_cp | 24 | -0.022022922 | 0.172996429 | -0.942390286 | 0 | 0 |
| BRD-K86301799__dipyridamole__trt_cp | 139 | -0.01207918 | 0.17309731 | -0.941996131 | 0 | 0 |
| BRD-K14550461__doxercalciferol__trt_cp | 61 | -0.020618379 | 0.176077602 | -0.930417031 | 0 | 0 |
| BRD-A19736161__ondansetron__trt_cp | 146 | -0.009829917 | 0.176952857 | -0.927040101 | 0 | 0 |
| BRD-K83144676__olmesartan__trt_cp | 37 | -0.023581042 | 0.177661151 | -0.924315066 | 0 | 0 |
| BRD-A90131694__alclometasone__trt_cp | 82 | -0.016638354 | 0.179365189 | -0.917787021 | 0 | 0 |
| BRD-K89923877__scopolamine__trt_cp | 82 | -0.013842988 | 0.179555235 | -0.917061395 | 0 | 0 |
| BRD-K84658672__nateglinide__trt_cp | 62 | -0.017783982 | 0.18036489 | -0.913975386 | 0 | 0 |
| BRD-K74514084__pazopanib__trt_cp | 201 | -0.017261939 | 0.180528765 | -0.913351833 | 0 | 0 |
| BRD-K09951645__dabrafenib__trt_cp | 143 | -0.043008003 | 0.184652694 | -0.89777524 | 0 | 0 |
| BRD-K60038276__irbesartan__trt_cp | 120 | -0.013328032 | 0.186090981 | -0.892393672 | 0 | 0 |
| BRD-K47886988__nalidixic-acid__trt_cp | 12 | -0.032622558 | 0.187829461 | -0.885923185 | 0 | 0 |
| BRD-A61154809__gliclazide__trt_cp | 11 | -0.039788241 | 0.187863259 | -0.885797758 | 0 | 0 |
| BRD-K87555556__amiodarone__trt_cp | 45 | -0.018635303 | 0.189464674 | -0.879870721 | 0 | 0 |
| BRD-K23369905__oxiconazole__trt_cp | 36 | -0.022721033 | 0.189497694 | -0.879748834 | 0 | 0 |
| BRD-A99633051__ethosuximide__trt_cp | 10 | -0.040248333 | 0.189779922 | -0.878707586 | 0 | 0 |
| BRD-K67977190__eprosartan__trt_cp | 45 | -0.015146659 | 0.190808585 | -0.87492049 | 0 | 0 |
| BRD-K73838513__cinacalcet__trt_cp | 65 | -0.016726428 | 0.192583073 | -0.868416901 | 0 | 0 |
| BRD-A39390670__rabeprazole__trt_cp | 46 | -0.017666248 | 0.193138228 | -0.866389762 | 0 | 0 |
| BRD-A11605036__thiocolchicoside__trt_cp | 54 | -0.018265227 | 0.19403011 | -0.863140506 | 0 | 0 |
| BRD-K06902185__minoxidil__trt_cp | 88 | -0.012049182 | 0.195280944 | -0.858598823 | 0 | 0 |
| BRD-A92630576__trimebutine__trt_cp | 37 | -0.020695202 | 0.195508303 | -0.857775202 | 0 | 0 |
| BRD-K89152108__liothyronine__trt_cp | 64 | -0.016492972 | 0.196003097 | -0.855984787 | 0 | 0 |
| BRD-K06895174__cisapride__trt_cp | 120 | -0.013125776 | 0.197576621 | -0.85030913 | 0 | 0 |
| BRD-A45664787__iloprost__trt_cp | 262 | -0.016141352 | 0.197638358 | -0.850087006 | 0 | 0 |
| BRD-A36010170__amcinonide__trt_cp | 83 | -0.016735846 | 0.200672021 | -0.839223253 | 0 | 0 |
| BRD-K58746048__flurandrenolide__trt_cp | 12 | -0.029902757 | 0.20522665 | -0.82309618 | 0 | 0 |
| BRD-K79602928__metformin__trt_cp | 222 | -0.013401215 | 0.20768946 | -0.814464466 | 0 | 0 |
| BRD-A51714012__venlafaxine__trt_cp | 132 | -0.011196724 | 0.209260293 | -0.808990543 | 0 | 0 |
| BRD-K43813806__naftifine__trt_cp | 11 | -0.033875572 | 0.209514006 | -0.808108696 | 0 | 0 |
| BRD-K18250272__propoxycaine__trt_cp | 75 | -0.012761841 | 0.212806764 | -0.796720236 | 0 | 0 |
| BRD-K46742498__alosetron__trt_cp | 36 | -0.018514727 | 0.21302706 | -0.795962004 | 0 | 0 |
| BRD-A33711280__metixene__trt_cp | 64 | -0.015757297 | 0.213301111 | -0.795019391 | 0 | 0 |
| BRD-A14798026__mestranol__trt_cp | 66 | -0.015032448 | 0.21601276 | -0.78573028 | 0 | 0 |
| BRD-A97454584__nadolol__trt_cp | 60 | -0.016093486 | 0.216205701 | -0.785071919 | 0 | 0 |
| BRD-K38305202__domperidone__trt_cp | 83 | -0.01367169 | 0.216578384 | -0.783801202 | 0 | 0 |
| BRD-K40530731__hyoscyamine__trt_cp | 65 | -0.013007747 | 0.216942321 | -0.782561526 | 0 | 0 |
| BRD-K63828191__raloxifene__trt_cp | 850 | -0.011622073 | 0.218687056 | -0.776635057 | 0 | 0 |
| BRD-A04553218__chlorphenamine__trt_cp | 128 | -0.009973927 | 0.218872679 | -0.776006143 | 0 | 0 |
| BRD-A23072235__pheniramine__trt_cp | 76 | -0.011841648 | 0.219308535 | -0.774530615 | 0 | 0 |
| BRD-K93461745__buspirone__trt_cp | 129 | -0.008958735 | 0.220123311 | -0.771776821 | 0 | 0 |
| BRD-A01636364__bupivacaine__trt_cp | 10 | -0.025492633 | 0.223035006 | -0.761983232 | 0 | 0 |
| BRD-K17849083__tranilast__trt_cp | 113 | -0.012196102 | 0.223495187 | -0.760442088 | 0 | 0 |
| BRD-K07220430__cinnarizine__trt_cp | 70 | -0.014315239 | 0.226096804 | -0.751762982 | 0 | 0 |
| BRD-K11469942__fluticasone__trt_cp | 11 | -0.028875493 | 0.226259051 | -0.751223597 | 0 | 0 |
| BRD-K90630139__butenafine__trt_cp | 12 | -0.027244902 | 0.226369312 | -0.75085716 | 0 | 0 |
| BRD-K83257731__chloropyramine__trt_cp | 11 | -0.026864656 | 0.226725641 | -0.74967365 | 0 | 0 |
| BRD-M19787235__dihydroergotamine__trt_cp | 8 | -0.033405619 | 0.227916217 | -0.745726853 | 0 | 0 |
| BRD-A57382968__piroxicam__trt_cp | 168 | -0.009263282 | 0.230342921 | -0.737717983 | 0 | 0 |
| BRD-A55393291__testosterone__trt_cp | 83 | -0.016455767 | 0.230559825 | -0.737004439 | 0 | 0 |
| BRD-A99833829__bethanechol__trt_cp | 56 | -0.012121826 | 0.233149947 | -0.728512543 | 0 | 0 |
| BRD-K39746403__erythromycin__trt_cp | 83 | -0.01419024 | 0.233367723 | -0.727800946 | 0 | 0 |
| BRD-K87210992__pancuronium__trt_cp | 11 | -0.030461828 | 0.237393256 | -0.714712823 | 0 | 0 |
| BRD-K06557128__voriconazole__trt_cp | 48 | -0.012913931 | 0.237491844 | -0.714393829 | 0 | 0 |
| BRD-K14177560__ethynodiol__trt_cp | 10 | -0.032310457 | 0.240267192 | -0.705443334 | 0 | 0 |
| BRD-K08111712__chloramphenicol__trt_cp | 10 | -0.025105736 | 0.244112647 | -0.693134268 | 0 | 0 |
| BRD-A56702968__nystatin__trt_cp | 12 | -0.028099483 | 0.247826336 | -0.681345859 | 0 | 0 |
| BRD-A88939772__estriol__trt_cp | 27 | -0.016771784 | 0.248605536 | -0.678884463 | 0 | 0 |
| BRD-A78971512__calcifediol__trt_cp | 27 | -0.017723455 | 0.249539288 | -0.675940257 | 0 | 0 |
| BRD-K40758068__efavirenz__trt_cp | 47 | -0.01457664 | 0.249754392 | -0.675262847 | 0 | 0 |
| BRD-A70461345__naloxone__trt_cp | 65 | -0.012456664 | 0.252828653 | -0.66561486 | 0 | 0 |
| BRD-K33299843__naproxen__trt_cp | 11 | -0.025971745 | 0.253093556 | -0.664786415 | 0 | 0 |
| BRD-K16195444__oxymetazoline__trt_cp | 64 | -0.0128048 | 0.253629545 | -0.663111588 | 0 | 0 |
| BRD-K99673372__montelukast__trt_cp | 10 | -0.025265641 | 0.257538872 | -0.650951581 | 0 | 0 |
| BRD-K47192521__icosapent__trt_cp | 76 | -0.010946998 | 0.260631718 | -0.641399071 | 0 | 0 |
| BRD-A44008656__doxylamine__trt_cp | 131 | -0.008163487 | 0.264830228 | -0.628524419 | 0 | 0 |
| BRD-K91289464__cefotetan__trt_cp | 12 | -0.025218772 | 0.266186544 | -0.624387568 | 0 | 0 |
| BRD-K67017579__cilostazol__trt_cp | 95 | -0.008631669 | 0.267104843 | -0.621592755 | 0 | 0 |
| BRD-A03427350__sulindac__trt_cp | 149 | -0.01685743 | 0.26770952 | -0.619755091 | 0 | 0 |
| BRD-K86161929__cortisone__trt_cp | 45 | -0.012137436 | 0.269185192 | -0.615279151 | 0 | 0 |
| BRD-A48430263__pioglitazone__trt_cp | 153 | -0.007903863 | 0.272601318 | -0.604964422 | 0 | 0 |
| BRD-M59543297__isometheptene__trt_cp | 11 | -0.025674974 | 0.277373244 | -0.590662636 | 0 | 0 |
| BRD-K38159897__chenodeoxycholic-acid__trt_cp | 10 | -0.023110902 | 0.281083474 | -0.579625862 | 0 | 0 |
| BRD-A54635441__levopropoxyphene__trt_cp | 11 | -0.022225492 | 0.284223333 | -0.570340664 | 0 | 0 |
| BRD-K30480208__torasemide__trt_cp | 99 | -0.00734358 | 0.28836031 | -0.558181255 | 0 | 0 |
| BRD-A53952395__prilocaine__trt_cp | 54 | -0.010103657 | 0.289443764 | -0.555010428 | 0 | 0 |
| BRD-A03623303__metoprolol__trt_cp | 65 | -0.010156504 | 0.29213313 | -0.547163718 | 0 | 0 |
| BRD-K84175871__pseudoephedrine__trt_cp | 71 | -0.008287586 | 0.297220942 | -0.532410256 | 0 | 0 |
| BRD-K70507123__doxofylline__trt_cp | 12 | -0.020425444 | 0.301111125 | -0.52120747 | 0 | 0 |
| BRD-K15108141__gemcitabine__trt_cp | 388 | -0.013816315 | 0.303181869 | -0.51527089 | 0 | 0 |
| BRD-K45149020__amcinonide__trt_cp | 11 | -0.020784711 | 0.307147913 | -0.503950977 | 0 | 0 |
| BRD-K28761384__zuclopenthixol__trt_cp | 72 | -0.009323083 | 0.308496045 | -0.500117861 | 0 | 0 |
| BRD-A23637604__oxymetholone__trt_cp | 37 | -0.015597757 | 0.312485611 | -0.488817056 | 0 | 0 |
| BRD-K19277754__paroxetine__trt_cp | 37 | -0.011708915 | 0.314240197 | -0.483866813 | 0 | 0 |
| BRD-K64931368__aminocaproic-acid__trt_cp | 11 | -0.019871006 | 0.314534649 | -0.483037235 | 0 | 0 |
| BRD-K97181089__amiloride__trt_cp | 169 | -0.005357038 | 0.31475823 | -0.482407548 | 0 | 0 |
| BRD-K81272440__dantrolene__trt_cp | 111 | -0.006517482 | 0.316251902 | -0.478205703 | 0 | 0 |
| BRD-K82795137__loratadine__trt_cp | 49 | -0.011247506 | 0.317435693 | -0.474881574 | 0 | 0 |
| BRD-K00259736__colchicine__trt_cp | 34 | -0.019414113 | 0.323148999 | -0.458911114 | 0 | 0 |
| BRD-A75935363__atracurium__trt_cp | 46 | -0.009292555 | 0.326871231 | -0.448569192 | 0 | 0 |
| BRD-K33106058__cytarabine__trt_cp | 48 | -0.013547974 | 0.330717739 | -0.437932138 | 0 | 0 |
| BRD-K50720187__flupirtine__trt_cp | 65 | -0.008836622 | 0.330759404 | -0.437817191 | 0 | 0 |
| BRD-A10977446__carvedilol__trt_cp | 153 | -0.006938123 | 0.331094292 | -0.436893504 | 0 | 0 |
| BRD-K53972329__ruxolitinib__trt_cp | 269 | -0.007125708 | 0.331721989 | -0.435163191 | 0 | 0 |
| BRD-K68804560__benzatropine__trt_cp | 11 | -0.016428486 | 0.332343194 | -0.433452059 | 0 | 0 |
| BRD-A60274948__bromocriptine__trt_cp | 53 | -0.009191844 | 0.336033651 | -0.423312464 | 0 | 0 |
| BRD-K57179821__crotamiton__trt_cp | 99 | -0.006711742 | 0.336527029 | -0.421960213 | 0 | 0 |
| BRD-A49906757__scopolamine__trt_cp | 72 | -0.006640382 | 0.33755295 | -0.419150837 | 0 | 0 |
| BRD-K31682896__sulfamethizole__trt_cp | 12 | -0.017785969 | 0.340612509 | -0.410792058 | 0 | 0 |
| BRD-A88080608__equilin__trt_cp | 70 | -0.006011894 | 0.342256789 | -0.406311715 | 0 | 0 |
| BRD-K74117820__nelfinavir__trt_cp | 36 | -0.012516692 | 0.345428698 | -0.397691781 | 0 | 0 |
| BRD-K81418486__vorinostat__trt_poscon | 2587 | -0.017674695 | 0.34582948 | -0.396604734 | 0 | 0 |
| BRD-A34817987__itraconazole__trt_cp | 223 | -0.005254558 | 0.346323689 | -0.395264931 | 0 | 0 |
| BRD-K88871508__lisuride__trt_cp | 65 | -0.006299063 | 0.346840326 | -0.393865081 | 0 | 0 |
| BRD-K82236179__dicoumarol__trt_cp | 12 | -0.016643011 | 0.347769608 | -0.391349093 | 0 | 0 |
| BRD-K97514127__vinorelbine__trt_cp | 37 | -0.01446306 | 0.347886841 | -0.391031866 | 0 | 0 |
| BRD-A83855350__naltrexone__trt_cp | 50 | -0.00994292 | 0.349666223 | -0.38622175 | 0 | 0 |
| BRD-K88789588__letrozole__trt_cp | 99 | -0.005418939 | 0.355253972 | -0.37117399 | 0 | 0 |
| BRD-K02227374__milnacipran__trt_cp | 37 | -0.008692678 | 0.355922407 | -0.369379582 | 0 | 0 |
| BRD-A43082555__loxoprofen__trt_cp | 37 | -0.009989427 | 0.357050901 | -0.366352845 | 0 | 0 |
| BRD-A23723433__paclitaxel__trt_cp | 229 | -0.007497448 | 0.35789099 | -0.364101817 | 0 | 0 |
| BRD-K33211335__dextromethorphan__trt_cp | 101 | -0.004710586 | 0.368558486 | -0.335673659 | 0 | 0 |
| BRD-K02275692__cefotiam__trt_cp | 70 | -0.005070026 | 0.370780821 | -0.329786033 | 0 | 0 |
| BRD-K43544024__oxandrolone__trt_cp | 10 | -0.014670949 | 0.371431336 | -0.328064794 | 0 | 0 |
| BRD-K78431006__crizotinib__trt_cp | 367 | -0.005395345 | 0.372492082 | -0.325260181 | 0 | 0 |
| BRD-K68620903__dydrogesterone__trt_cp | 72 | -0.00543732 | 0.373239589 | -0.32328531 | 0 | 0 |
| BRD-K77641333__naphazoline__trt_cp | 72 | -0.005818065 | 0.373398587 | -0.322865409 | 0 | 0 |
| BRD-K53815609__nitrofural__trt_cp | 12 | -0.012498681 | 0.373449739 | -0.322730332 | 0 | 0 |
| BRD-K28542495__benzydamine__trt_cp | 10 | -0.012287891 | 0.376159412 | -0.315583288 | 0 | 0 |
| BRD-K85090592__pilocarpine__trt_cp | 49 | -0.005611208 | 0.376319974 | -0.315160299 | 0 | 0 |
| BRD-K96037667__norethindrone__trt_cp | 44 | -0.006980955 | 0.378621466 | -0.309103323 | 0 | 0 |
| BRD-A87445400__yohimbine__trt_cp | 83 | -0.004053484 | 0.387356051 | -0.286216767 | 0 | 0 |
| BRD-K60160658__tiagabine__trt_cp | 37 | -0.006437275 | 0.393530224 | -0.270129734 | 0 | 0 |
| BRD-A33070992__bacampicilline__trt_cp | 12 | -0.010271904 | 0.393823937 | -0.269366226 | 0 | 0 |
| BRD-A23712469__bacitracin__trt_cp | 11 | -0.009746645 | 0.396050869 | -0.263582379 | 0 | 0 |
| BRD-K29458283__chlorambucil__trt_cp | 166 | -0.004268101 | 0.398645044 | -0.256855805 | 0 | 0 |
| BRD-K63430059__methoxsalen__trt_cp | 120 | -0.003484525 | 0.399963268 | -0.253442181 | 0 | 0 |
| BRD-K73947551__amifostine__trt_cp | 119 | -0.006175084 | 0.403502975 | -0.24429036 | 0 | 0 |
| BRD-A89175223__bisoprolol__trt_cp | 119 | -0.003124619 | 0.407835922 | -0.233115339 | 0 | 0 |
| BRD-K37848908__ceforanide__trt_cp | 64 | -0.004110112 | 0.408379108 | -0.231716498 | 0 | 0 |
| BRD-A13650332__flucloxacillin__trt_cp | 63 | -0.004507909 | 0.411296723 | -0.224210586 | 0 | 0 |
| BRD-K82481048__prednisolone__trt_cp | 9 | -0.008472686 | 0.412457478 | -0.221227938 | 0 | 0 |
| BRD-K62737565__gabapentin__trt_cp | 11 | -0.008445477 | 0.41621113 | -0.211595961 | 0 | 0 |
| BRD-K57773246__colistin__trt_cp | 12 | -0.00828432 | 0.417269738 | -0.208883128 | 0 | 0 |
| BRD-K92428153__mycophenolate-mofetil__trt_cp | 63 | -0.005851231 | 0.417811472 | -0.207495452 | 0 | 0 |
| BRD-K90711641__dopamine__trt_cp | 24 | -0.004965682 | 0.418250919 | -0.206370084 | 0 | 0 |
| BRD-A84205515__estriol__trt_cp | 66 | -0.003259387 | 0.422210249 | -0.19624233 | 0 | 0 |
| BRD-K37516142__idebenone__trt_cp | 49 | -0.004106702 | 0.424419813 | -0.190599188 | 0 | 0 |
| BRD-K13688115__medrysone__trt_cp | 12 | -0.007212839 | 0.425820304 | -0.18702554 | 0 | 0 |
| BRD-K85503079__perospirone__trt_cp | 35 | -0.00445109 | 0.426130273 | -0.186234913 | 0 | 0 |
| BRD-K11399644__phenformin__trt_cp | 99 | -0.002916135 | 0.430553782 | -0.174964454 | 0 | 0 |
| BRD-K57886322__fluocinonide__trt_cp | 44 | -0.003449042 | 0.434108772 | -0.165923004 | 0 | 0 |
| BRD-K19687926__lapatinib__trt_cp | 244 | -0.002721771 | 0.435428147 | -0.162570908 | 0 | 0 |
| BRD-A63934767__alclometasone__trt_cp | 12 | -0.005634379 | 0.435633388 | -0.162049625 | 0 | 0 |
| BRD-K73999723__telmisartan__trt_cp | 113 | -0.002254847 | 0.43656671 | -0.159679665 | 0 | 0 |
| BRD-K71499074__diclofenamide__trt_cp | 83 | -0.002205245 | 0.439759534 | -0.151578912 | 0 | 0 |
| BRD-K49111258__prazosin__trt_cp | 110 | -0.001970809 | 0.441127203 | -0.148111967 | 0 | 0 |
| BRD-K74141488__naftifine__trt_cp | 70 | -0.002351929 | 0.441287651 | -0.14770536 | 0 | 0 |
| BRD-K18922609__neostigmine__trt_cp | 12 | -0.005543501 | 0.442898505 | -0.143624479 | 0 | 0 |
| BRD-A54490543__pirlindole__trt_cp | 63 | -0.00270175 | 0.444253719 | -0.140193081 | 0 | 0 |
| BRD-K33425534__exemestane__trt_cp | 37 | -0.00422611 | 0.445543378 | -0.136929203 | 0 | 0 |
| BRD-K79131256__albendazole__trt_cp | 162 | -0.00290008 | 0.447439119 | -0.132134083 | 0 | 0 |
| BRD-K79759031__artesunate__trt_cp | 37 | -0.00381408 | 0.4475165 | -0.13193842 | 0 | 0 |
| BRD-K06335600__tizanidine__trt_cp | 55 | -0.002428952 | 0.452791341 | -0.118612096 | 0 | 0 |
| BRD-K30816563__ranitidine__trt_cp | 91 | -0.00175453 | 0.45730152 | -0.107234383 | 0 | 0 |
| BRD-K00198051__zuclopenthixol__trt_cp | 11 | -0.003685544 | 0.460619375 | -0.098873448 | 0 | 0 |
| BRD-K41903098__diphenoxylate__trt_cp | 37 | -0.002207245 | 0.467943057 | -0.08044151 | 0 | 0 |
| BRD-K86873305__piperacillin__trt_cp | 43 | -0.002152397 | 0.467979239 | -0.080350521 | 0 | 0 |
| BRD-A39969961__eplerenone__trt_cp | 62 | -0.001371494 | 0.469966786 | -0.075353354 | 0 | 0 |
| BRD-K39915878__loxapine__trt_cp | 129 | -0.001076278 | 0.473269059 | -0.06705475 | 0 | 0 |
| BRD-K59456551__methotrexate__trt_cp | 46 | -0.001945385 | 0.474901234 | -0.062954837 | 0 | 0 |
| BRD-K05851096__lacidipine__trt_cp | 12 | -0.002703974 | 0.475596187 | -0.061209489 | 0 | 0 |
| BRD-K29582677__flunarizine__trt_cp | 83 | -0.001024859 | 0.476101085 | -0.059941573 | 0 | 0 |
| BRD-K31333309__dantrolene__trt_cp | 10 | -0.002347217 | 0.476107415 | -0.059925676 | 0 | 0 |
| BRD-K01612348__meropenem__trt_cp | 81 | -9.43E-04 | 0.478756846 | -0.053273879 | 0 | 0 |
| BRD-M45964048__verteporfin__trt_cp | 85 | -8.51E-04 | 0.484889335 | -0.037885881 | 0 | 0 |
| BRD-K44993696__atenolol__trt_cp | 11 | -0.001311843 | 0.486237226 | -0.034505004 | 0 | 0 |
| BRD-K32273377__sulfadiazine__trt_cp | 10 | -5.70E-04 | 0.493948811 | -0.015168663 | 0 | 0 |
| BRD-K05673000__dicloxacillin__trt_cp | 57 | -3.31E-04 | 0.494128519 | -0.014718152 | 0 | 0 |
| BRD-K22429181__retinol__trt_cp | 85 | 5.84E-05 | 0.50112162 | 0.002811488 | 0 | 0 |
| BRD-A26711594__nicardipine__trt_cp | 234 | 5.09E-05 | 0.501237037 | 0.003100796 | 0 | 0 |
| BRD-K25841245__pravastatin__trt_cp | 9 | 4.50E-04 | 0.503966538 | 0.009942801 | 0 | 0 |
| BRD-K37814297__acepromazine__trt_cp | 49 | 3.13E-04 | 0.505205142 | 0.013047727 | 0 | 0 |
| BRD-K10706131__rivastigmine__trt_cp | 11 | 8.35E-04 | 0.507677657 | 0.01924622 | 0 | 0 |
| BRD-A93424738__dexamethasone__trt_cp | 86 | 3.58E-04 | 0.508505256 | 0.021321129 | 0 | 0 |
| BRD-A43974499__reboxetine__trt_cp | 68 | 4.49E-04 | 0.510883919 | 0.027285324 | 0 | 0 |
| BRD-K79116891__proxymetacaine__trt_cp | 166 | 5.49E-04 | 0.518407336 | 0.046156733 | 0 | 0 |
| BRD-K05926469__lenalidomide__trt_cp | 64 | 0.001418859 | 0.524327924 | 0.061018906 | 0 | 0 |
| BRD-A00546892__biperiden__trt_cp | 82 | 8.85E-04 | 0.526840531 | 0.067330072 | 0 | 0 |
| BRD-A24891640__nimodipine__trt_cp | 46 | 0.001817436 | 0.530395022 | 0.076262881 | 0 | 0 |
| BRD-K24473282__isoprenaline__trt_cp | 11 | 0.002970862 | 0.531948977 | 0.080170006 | 0 | 0 |
| BRD-K72542090__clodronic-acid__trt_cp | 12 | 0.003296216 | 0.532986858 | 0.082780235 | 0 | 0 |
| BRD-K58626373__dexfenfluramine__trt_cp | 12 | 0.00371938 | 0.533617226 | 0.084365863 | 0 | 0 |
| BRD-A37444045__hydralazine__trt_cp | 10 | 0.003975167 | 0.534522178 | 0.086642549 | 0 | 0 |
| BRD-K18194590__mephentermine__trt_cp | 81 | 0.001570071 | 0.534771545 | 0.087269987 | 0 | 0 |
| BRD-A90799790__isradipine__trt_cp | 63 | 0.001503036 | 0.534921778 | 0.087648007 | 0 | 0 |
| BRD-K70511574__sunitinib__trt_cp | 198 | 0.001373878 | 0.535563863 | 0.089263785 | 0 | 0 |
| BRD-K65544384__aminohippuric-acid__trt_cp | 11 | 0.004021673 | 0.536020028 | 0.090411846 | 0 | 0 |
| BRD-K25991148__warfarin__trt_cp | 67 | 0.001677815 | 0.537153764 | 0.093265711 | 0 | 0 |
| BRD-M98649031__dimenhydrinate__trt_cp | 10 | 0.003739802 | 0.537735481 | 0.094730316 | 0 | 0 |
| BRD-K04412738__tramadol__trt_cp | 44 | 0.002559333 | 0.538912246 | 0.097693712 | 0 | 0 |
| BRD-K93923542__exemestane__trt_cp | 12 | 0.004451257 | 0.541970245 | 0.10539862 | 0 | 0 |
| BRD-K37130586__olopatadine__trt_cp | 11 | 0.004581518 | 0.544931038 | 0.112864572 | 0 | 0 |
| BRD-K30685142__levothyroxine__trt_cp | 12 | 0.004505206 | 0.546021045 | 0.115614703 | 0 | 0 |
| BRD-A96255180__ribavirin__trt_cp | 46 | 0.003022186 | 0.550859653 | 0.127833556 | 0 | 0 |
| BRD-A87719232__naproxen__trt_cp | 82 | 0.001932655 | 0.551040531 | 0.128290684 | 0 | 0 |
| BRD-K13994703__propranolol__trt_cp | 11 | 0.005253777 | 0.551354606 | 0.129084499 | 0 | 0 |
| BRD-A13188892__doxazosin__trt_cp | 56 | 0.002709615 | 0.554666882 | 0.13746123 | 0 | 0 |
| BRD-K76018977__cefotaxime__trt_cp | 10 | 0.005863601 | 0.555257219 | 0.138955186 | 0 | 0 |
| BRD-K82143716__flucytosine__trt_cp | 48 | 0.00329511 | 0.557139862 | 0.143721649 | 0 | 0 |
| BRD-K87226815__cycloserine__trt_cp | 93 | 0.002290133 | 0.561034006 | 0.153591313 | 0 | 0 |
| BRD-K41260949__valproic-acid__trt_cp | 427 | 0.001595513 | 0.562805905 | 0.158087068 | 0 | 0 |
| BRD-U88459701__atorvastatin__trt_cp | 207 | 0.002962402 | 0.563117854 | 0.158878891 | 0 | 0 |
| BRD-K93754473__tamoxifen__trt_cp | 893 | 0.002120171 | 0.564135813 | 0.161463479 | 0 | 0 |
| BRD-A73581086__ergometrine__trt_cp | 72 | 0.002557219 | 0.56508963 | 0.163886189 | 0 | 0 |
| BRD-A28095882__zonisamide__trt_cp | 24 | 0.004509461 | 0.56829013 | 0.172022628 | 0 | 0 |
| BRD-K50938786__ropivacaine__trt_cp | 48 | 0.003397986 | 0.569553071 | 0.175236433 | 0 | 0 |
| BRD-K77771411__moxonidine__trt_cp | 111 | 0.00198883 | 0.570383373 | 0.177350286 | 0 | 0 |
| BRD-K86003836__flubendazole__trt_cp | 46 | 0.006206084 | 0.571222119 | 0.179486442 | 0 | 0 |
| BRD-A12560204__nitrendipine__trt_cp | 35 | 0.003938378 | 0.574556887 | 0.187987785 | 0 | 0 |
| BRD-K04210847__tamoxifen__trt_cp | 53 | 0.00523866 | 0.57500936 | 0.189142313 | 0 | 0 |
| BRD-K48617017__dorzolamide__trt_cp | 11 | 0.007280024 | 0.57646891 | 0.192868213 | 0 | 0 |
| BRD-A80638690__floxuridine__trt_cp | 42 | 0.00517132 | 0.58034912 | 0.202786692 | 0 | 0 |
| BRD-K08547377__irinotecan__trt_cp | 114 | 0.006754107 | 0.5830536 | 0.209711562 | 0 | 0 |
| BRD-A58753560__budesonide__trt_cp | 12 | 0.009338527 | 0.587810931 | 0.221917461 | 0 | 0 |
| BRD-K34888156__theobromine__trt_cp | 11 | 0.008804907 | 0.587996017 | 0.222392993 | 0 | 0 |
| BRD-K07265709__razoxane__trt_cp | 66 | 0.004766215 | 0.589362028 | 0.225904183 | 0 | 0 |
| BRD-K99107520__felbamate__trt_cp | 104 | 0.003631466 | 0.589719422 | 0.226823286 | 0 | 0 |
| BRD-K63899271__biotin__trt_cp | 12 | 0.008654556 | 0.589893515 | 0.227271067 | 0 | 0 |
| BRD-K31283835__tofacitinib__trt_cp | 63 | 0.003982768 | 0.590288644 | 0.228287537 | 0 | 0 |
| BRD-K37249724__astemizole__trt_cp | 10 | 0.011032441 | 0.593363957 | 0.23620695 | 0 | 0 |
| BRD-K85767870__adenosine-phosphate__trt_cp | 10 | 0.010322159 | 0.594501523 | 0.239140092 | 0 | 0 |
| BRD-K29113274__ketoconazole__trt_cp | 33 | 0.007098824 | 0.59474553 | 0.239769518 | 0 | 0 |
| BRD-K69260336__pivampicillin__trt_cp | 11 | 0.008796088 | 0.599313273 | 0.251569995 | 0 | 0 |
| BRD-K82381502__acetylcholine__trt_cp | 45 | 0.00554143 | 0.601541193 | 0.257338318 | 0 | 0 |
| BRD-A83892713__rifampicin__trt_cp | 44 | 0.006683906 | 0.604217987 | 0.264280188 | 0 | 0 |
| BRD-K01228321__cisplatin__trt_cp | 108 | 0.00661883 | 0.605823411 | 0.268449712 | 0 | 0 |
| BRD-K90789829__nefazodone__trt_cp | 102 | 0.00405248 | 0.608212973 | 0.274664439 | 0 | 0 |
| BRD-K54283240__cefixime__trt_cp | 12 | 0.010242206 | 0.609355271 | 0.277639043 | 0 | 0 |
| BRD-A08660406__iodixanol__trt_cp | 11 | 0.010950808 | 0.609377023 | 0.277695711 | 0 | 0 |
| BRD-K11742128__triprolidine__trt_cp | 53 | 0.005339673 | 0.612515253 | 0.285880703 | 0 | 0 |
| BRD-K15916496__clotrimazole__trt_cp | 47 | 0.007047371 | 0.613325877 | 0.287998031 | 0 | 0 |
| BRD-K43744935__tamoxifen__trt_cp | 71 | 0.005482478 | 0.613880754 | 0.289448099 | 0 | 0 |
| BRD-K20152659__gamma-homolinolenic-acid__trt_cp | 52 | 0.008347799 | 0.615928684 | 0.29480528 | 0 | 0 |
| BRD-K34415467__trimethobenzamide__trt_cp | 73 | 0.005191472 | 0.61627241 | 0.295705261 | 0 | 0 |
| BRD-K28143534__cyproheptadine__trt_cp | 58 | 0.008417384 | 0.61676836 | 0.297004229 | 0 | 0 |
| BRD-A11170096__pravastatin__trt_cp | 44 | 0.005837804 | 0.619124304 | 0.303181702 | 0 | 0 |
| BRD-A17462676__bambuterol__trt_cp | 9 | 0.014899983 | 0.619439755 | 0.304009714 | 0 | 0 |
| BRD-K54529596__captopril__trt_cp | 99 | 0.004703814 | 0.622358637 | 0.311681319 | 0 | 0 |
| BRD-M61872305__mephentermine__trt_cp | 11 | 0.014645727 | 0.624379484 | 0.317003387 | 0 | 0 |
| BRD-K48578705__methyldopa__trt_cp | 10 | 0.015865439 | 0.624415683 | 0.317098802 | 0 | 0 |
| BRD-K90553655__testosterone__trt_cp | 67 | 0.006196088 | 0.627947887 | 0.326423148 | 0 | 0 |
| BRD-A29322418__canrenoic-acid__trt_cp | 82 | 0.005315219 | 0.630447536 | 0.333038888 | 0 | 0 |
| BRD-A94709349__metaxalone__trt_cp | 62 | 0.007402955 | 0.63772757 | 0.352391253 | 0 | 0 |
| BRD-K98530306__clonidine__trt_cp | 315 | 0.003513503 | 0.638217177 | 0.353697434 | 0 | 0 |
| BRD-K52827117__aprepitant__trt_cp | 12 | 0.014820847 | 0.638692219 | 0.354965333 | 0 | 0 |
| BRD-K51485625__ritonavir__trt_cp | 37 | 0.009111138 | 0.639738187 | 0.357759067 | 0 | 0 |
| BRD-A79903587__tegafur__trt_cp | 37 | 0.009032651 | 0.641726883 | 0.363078513 | 0 | 0 |
| BRD-K13032584__procarbazine__trt_cp | 111 | 0.004848029 | 0.645394539 | 0.372916056 | 0 | 0 |
| BRD-A50311610__meclozine__trt_cp | 122 | 0.004641882 | 0.646069117 | 0.374729344 | 0 | 0 |
| BRD-K81855038__roxatidine__trt_cp | 49 | 0.00761143 | 0.646882947 | 0.376918588 | 0 | 0 |
| BRD-K35586044__yohimbine__trt_cp | 53 | 0.007293454 | 0.651726534 | 0.389985957 | 0 | 0 |
| BRD-K78113049__enoxacin__trt_cp | 11 | 0.015652167 | 0.654728752 | 0.398118965 | 0 | 0 |
| BRD-K70505054__ranitidine__trt_cp | 63 | 0.007899181 | 0.655877414 | 0.401237637 | 0 | 0 |
| BRD-A47598013__citalopram__trt_cp | 147 | 0.00648324 | 0.659928088 | 0.412266876 | 0 | 0 |
| BRD-K56800335__guanabenz__trt_cp | 52 | 0.00835585 | 0.661802987 | 0.417388826 | 0 | 0 |
| BRD-A74269027__isoxsuprine__trt_cp | 82 | 0.006690388 | 0.663011792 | 0.420696913 | 0 | 0 |
| BRD-A39189014__methoxamine__trt_cp | 12 | 0.015263411 | 0.667860386 | 0.434012688 | 0 | 0 |
| BRD-K46937689__phenazone__trt_cp | 127 | 0.005718203 | 0.668389481 | 0.435470376 | 0 | 0 |
| BRD-A73635141__hydrocortisone__trt_cp | 27 | 0.011678808 | 0.669484851 | 0.438491131 | 0 | 0 |
| BRD-K99300445__antazoline__trt_cp | 10 | 0.017047573 | 0.670949938 | 0.442537745 | 0 | 0 |
| BRD-A51382177__fosinopril__trt_cp | 45 | 0.009370919 | 0.671876063 | 0.445099466 | 0 | 0 |
| BRD-A14566392__scopolamine__trt_cp | 72 | 0.008437671 | 0.672493479 | 0.446808903 | 0 | 0 |
| BRD-K10670311__sulfasalazine__trt_cp | 49 | 0.007968755 | 0.67294686 | 0.44806501 | 0 | 0 |
| BRD-A12832192__streptozotocin__trt_cp | 27 | 0.013652229 | 0.673287342 | 0.44900879 | 0 | 0 |
| BRD-K44942604__pentolinium__trt_cp | 10 | 0.017462347 | 0.673497505 | 0.449591539 | 0 | 0 |
| BRD-K37270826__mifepristone__trt_cp | 125 | 0.006376637 | 0.675365398 | 0.454777661 | 0 | 0 |
| BRD-K44442813__pidotimod__trt_cp | 36 | 0.009982931 | 0.676252071 | 0.457243745 | 0 | 0 |
| BRD-M73371355__apomorphine__trt_cp | 10 | 0.019147274 | 0.676807695 | 0.458790512 | 0 | 0 |
| BRD-A49035384__metacycline__trt_cp | 11 | 0.019734364 | 0.67747234 | 0.460642213 | 0 | 0 |
| BRD-A07765530__epinephrine__trt_cp | 56 | 0.008044312 | 0.681326409 | 0.471411113 | 0 | 0 |
| BRD-A42628519__iopanoic-acid__trt_cp | 93 | 0.00807717 | 0.682182481 | 0.473810512 | 0 | 0 |
| BRD-K74741811__norgestrel__trt_cp | 12 | 0.017744104 | 0.683602343 | 0.477796134 | 0 | 0 |
| BRD-K01663662__diphenidol__trt_cp | 74 | 0.008482109 | 0.689667609 | 0.4949084 | 0 | 0 |
| BRD-K34685430__methylergometrine__trt_cp | 62 | 0.010554681 | 0.690697171 | 0.497827461 | 0 | 0 |
| BRD-K77987382__mebendazole__trt_cp | 159 | 0.010591422 | 0.692269817 | 0.502294515 | 0 | 0 |
| BRD-K71289571__zafirlukast__trt_cp | 94 | 0.009099871 | 0.693162382 | 0.50483428 | 0 | 0 |
| BRD-K22901619__norgestimate__trt_cp | 11 | 0.020125633 | 0.693769098 | 0.506562531 | 0 | 0 |
| BRD-K24023109__diltiazem__trt_cp | 164 | 0.006001957 | 0.694736185 | 0.509320444 | 0 | 0 |
| BRD-A99117172__hydroxychloroquine__trt_cp | 33 | 0.014032661 | 0.698022005 | 0.518720034 | 0 | 0 |
| BRD-K05111602__ramipril__trt_cp | 11 | 0.019748567 | 0.701785224 | 0.52954195 | 0 | 0 |
| BRD-A97104540__fenoterol__trt_cp | 95 | 0.008793369 | 0.704112196 | 0.536264722 | 0 | 0 |
| BRD-K04196797__oxcarbazepine__trt_cp | 48 | 0.011817029 | 0.705670483 | 0.540780284 | 0 | 0 |
| BRD-K35240538__methylprednisolone__trt_cp | 46 | 0.012098134 | 0.705690032 | 0.540837003 | 0 | 0 |
| BRD-K85518562__cilastatin__trt_cp | 37 | 0.013141969 | 0.706334924 | 0.542709037 | 0 | 0 |
| BRD-K99260425__etodolac__trt_cp | 66 | 0.008123055 | 0.707610704 | 0.546418069 | 0 | 0 |
| BRD-A47829399__artesunate__trt_cp | 282 | 0.00820872 | 0.709550506 | 0.552072053 | 0 | 0 |
| BRD-K87024524__phenelzine__trt_cp | 91 | 0.008583273 | 0.710206725 | 0.553988743 | 0 | 0 |
| BRD-A64092382__mexiletine__trt_cp | 127 | 0.006540877 | 0.710610157 | 0.5551681 | 0 | 0 |
| BRD-K84937637__sirolimus__trt_cp | 314 | 0.019429226 | 0.713564271 | 0.563827613 | 0 | 0 |
| BRD-K33453211__levocabastine__trt_cp | 70 | 0.009120609 | 0.715106129 | 0.56836413 | 0 | 0 |
| BRD-K51033547__tramadol__trt_cp | 63 | 0.009090925 | 0.718095224 | 0.57719231 | 0 | 0 |
| BRD-K22936972__diphenylpyraline__trt_cp | 12 | 0.022784625 | 0.720082647 | 0.583087042 | 0 | 0 |
| BRD-K30114692__methenamine__trt_cp | 11 | 0.020152553 | 0.721602089 | 0.587607431 | 0 | 0 |
| BRD-K59720958__finasteride__trt_cp | 12 | 0.028305576 | 0.725367298 | 0.598861263 | 0 | 0 |
| BRD-K82624463__prednisone__trt_cp | 10 | 0.02363136 | 0.726766236 | 0.603061873 | 0 | 0 |
| BRD-K42693031__glimepiride__trt_cp | 65 | 0.011407454 | 0.727942883 | 0.606603265 | 0 | 0 |
| BRD-K85119730__tolbutamide__trt_cp | 118 | 0.007421591 | 0.729562784 | 0.611491216 | 0 | 0 |
| BRD-K97752965__nicorandil__trt_cp | 47 | 0.014148601 | 0.729766577 | 0.612107184 | 0 | 0 |
| BRD-K91290917__amodiaquine__trt_cp | 80 | 0.011447622 | 0.730506853 | 0.61434663 | 0 | 0 |
| BRD-K95763993__trapidil__trt_cp | 76 | 0.011592566 | 0.730868365 | 0.615441382 | 0 | 0 |
| BRD-A57133233__chlorcyclizine__trt_cp | 12 | 0.023273309 | 0.731420343 | 0.617114336 | 0 | 0 |
| BRD-K02867583__minaprine__trt_cp | 63 | 0.011129783 | 0.732050005 | 0.619024848 | 0 | 0 |
| BRD-K94830329__ataluren__trt_cp | 45 | 0.011966685 | 0.73751785 | 0.635711775 | 0 | 0 |
| BRD-K70778732__trazodone__trt_cp | 131 | 0.007509036 | 0.740877997 | 0.646054586 | 0 | 0 |
| BRD-A49148672__galantamine__trt_cp | 52 | 0.011787258 | 0.741795803 | 0.648891677 | 0 | 0 |
| BRD-A15297126__fluocinonide__trt_cp | 53 | 0.018354655 | 0.742085191 | 0.649787308 | 0 | 0 |
| BRD-A19633847__perhexiline__trt_cp | 284 | 0.017008064 | 0.742395296 | 0.650747634 | 0 | 0 |
| BRD-K74927006__cinnarizine__trt_cp | 10 | 0.024473977 | 0.742618713 | 0.651439879 | 0 | 0 |
| BRD-A03216249__mepivacaine__trt_cp | 59 | 0.012043343 | 0.743760722 | 0.654983223 | 0 | 0 |
| BRD-A78391468__prednisolone__trt_cp | 83 | 0.01177541 | 0.744495085 | 0.6572661 | 0 | 0 |
| BRD-A18929998__cytarabine__trt_cp | 24 | 0.02556982 | 0.744700214 | 0.657904386 | 0 | 0 |
| BRD-A31801025__formestane__trt_cp | 61 | 0.011719354 | 0.745084914 | 0.659102155 | 0 | 0 |
| BRD-K73541271__etofenamate__trt_cp | 12 | 0.0238413 | 0.747978861 | 0.668143058 | 0 | 0 |
| BRD-A35108200__dexamethasone__trt_cp | 238 | 0.009506615 | 0.752907533 | 0.683667844 | 0 | 0 |
| BRD-K70592963__phenindione__trt_cp | 12 | 0.022735697 | 0.756681421 | 0.695667381 | 0 | 0 |
| BRD-A92177080__betamethasone__trt_cp | 81 | 0.011227732 | 0.759730492 | 0.70543589 | 0 | 0 |
| BRD-K08252256__diclofenac__trt_cp | 156 | 0.008662221 | 0.761081358 | 0.709785303 | 0 | 0 |
| BRD-A58837096__ribostamycin__trt_cp | 12 | 0.030379528 | 0.761135855 | 0.709961049 | 0 | 0 |
| BRD-K35531059__molsidomine__trt_cp | 78 | 0.010954912 | 0.761615382 | 0.711508418 | 0 | 0 |
| BRD-A17828570__fulvestrant__trt_cp | 10 | 0.034956143 | 0.761930621 | 0.712526579 | 0 | 0 |
| BRD-K93880783__stavudine__trt_cp | 90 | 0.010101521 | 0.764687971 | 0.721464041 | 0 | 0 |
| BRD-K67847053__guanabenz__trt_cp | 91 | 0.012612839 | 0.766112214 | 0.726103096 | 0 | 0 |
| BRD-K45158365__valsartan__trt_cp | 98 | 0.01017474 | 0.766517371 | 0.727425632 | 0 | 0 |
| BRD-K37187815__cefuroxime__trt_cp | 12 | 0.031785506 | 0.767264095 | 0.729866469 | 0 | 0 |
| BRD-K08356259__xylometazoline__trt_cp | 10 | 0.031293027 | 0.772120885 | 0.745849671 | 0 | 0 |
| BRD-K39188321__betamethasone__trt_cp | 65 | 0.014890039 | 0.774594496 | 0.754063643 | 0 | 0 |
| BRD-A99939097__carisoprodol__trt_cp | 12 | 0.031771381 | 0.777300642 | 0.763108458 | 0 | 0 |
| BRD-A46186775__hydrocortisone__trt_cp | 53 | 0.016420911 | 0.777747194 | 0.764606978 | 0 | 0 |
| BRD-K86307448__allopurinol__trt_cp | 11 | 0.029960465 | 0.778518215 | 0.767198389 | 0 | 0 |
| BRD-K63675182__triflupromazine__trt_cp | 236 | 0.020609481 | 0.7786457 | 0.767627364 | 0 | 0 |
| BRD-A65282128__cefazolin__trt_cp | 45 | 0.014088475 | 0.779032614 | 0.768930159 | 0 | 0 |
| BRD-K99121711__cinchocaine__trt_cp | 10 | 0.02803917 | 0.779550071 | 0.770674553 | 0 | 0 |
| BRD-A87848830__bimatoprost__trt_cp | 44 | 0.018041764 | 0.779693989 | 0.771160132 | 0 | 0 |
| BRD-A79479878__testosterone__trt_cp | 71 | 0.012833077 | 0.780159809 | 0.77273305 | 0 | 0 |
| BRD-K42098891__protriptyline__trt_cp | 93 | 0.014701537 | 0.78042298 | 0.773622539 | 0 | 0 |
| BRD-A27887842__prednisolone__trt_cp | 80 | 0.013876387 | 0.780719516 | 0.774625527 | 0 | 0 |
| BRD-K75527158__alendronic-acid__trt_cp | 36 | 0.016644004 | 0.781068024 | 0.775805305 | 0 | 0 |
| BRD-A00993607__alprenolol__trt_cp | 94 | 0.012193095 | 0.784015656 | 0.785827271 | 0 | 0 |
| BRD-A39210489__chlortetracycline__trt_cp | 10 | 0.028882093 | 0.788259062 | 0.800395173 | 0 | 0 |
| BRD-A23034328__clindamycin__trt_cp | 10 | 0.02747991 | 0.788712446 | 0.801961709 | 0 | 0 |
| BRD-A98431941__ephedrine__trt_cp | 53 | 0.014550995 | 0.791656242 | 0.81218146 | 0 | 0 |
| BRD-K59632282__quinidine__trt_cp | 63 | 0.015504591 | 0.791983282 | 0.813322054 | 0 | 0 |
| BRD-K30207000__dexamethasone__trt_cp | 10 | 0.037356254 | 0.792275703 | 0.814342805 | 0 | 0 |
| BRD-K56810756__fomepizole__trt_cp | 12 | 0.036028373 | 0.792586471 | 0.81542853 | 0 | 0 |
| BRD-K69116396__sertindole__trt_cp | 12 | 0.04340008 | 0.793581223 | 0.818910369 | 0 | 0 |
| BRD-A60052764__flucloxacillin__trt_cp | 11 | 0.035614052 | 0.793929608 | 0.820132137 | 0 | 0 |
| BRD-K17743125__belinostat__trt_cp | 81 | 0.046504318 | 0.794785138 | 0.823137643 | 0 | 0 |
| BRD-A52997285__tetracycline__trt_cp | 10 | 0.034158455 | 0.795052781 | 0.824079411 | 0 | 0 |
| BRD-K52735702__cefdinir__trt_cp | 49 | 0.015481617 | 0.795629808 | 0.826112311 | 0 | 0 |
| BRD-K03406345__azacitidine__trt_cp | 71 | 0.02753577 | 0.797149072 | 0.83148118 | 0 | 0 |
| BRD-K13027675__fluorometholone__trt_cp | 10 | 0.034467544 | 0.799273918 | 0.839030553 | 0 | 0 |
| BRD-K34469523__levalbuterol__trt_cp | 11 | 0.036725386 | 0.801314542 | 0.846325984 | 0 | 0 |
| BRD-A61793559__metolazone__trt_cp | 93 | 0.011960349 | 0.801600863 | 0.847353214 | 0 | 0 |
| BRD-K47631482__bromhexine__trt_cp | 95 | 0.014759898 | 0.803277361 | 0.853386007 | 0 | 0 |
| BRD-K13078532__hydrochlorothiazide__trt_cp | 10 | 0.039995099 | 0.805795434 | 0.862506 | 0 | 0 |
| BRD-K61993165__niacin__trt_cp | 49 | 0.020489185 | 0.80629744 | 0.864332758 | 0 | 0 |
| BRD-K18618618__cimetidine__trt_cp | 72 | 0.013746118 | 0.807285003 | 0.86793486 | 0 | 0 |
| BRD-A98378129__talniflumate__trt_cp | 71 | 0.014445705 | 0.807293295 | 0.867965152 | 0 | 0 |
| BRD-A97674275__ranolazine__trt_cp | 109 | 0.011420502 | 0.807651873 | 0.869275876 | 0 | 0 |
| BRD-K30649484__mafenide__trt_cp | 79 | 0.016920157 | 0.809318915 | 0.875389208 | 0 | 0 |
| BRD-K99621550__tubocurarine__trt_cp | 82 | 0.015161373 | 0.810853305 | 0.881045138 | 0 | 0 |
| BRD-K53737926__amitriptyline__trt_cp | 210 | 0.016979728 | 0.810876869 | 0.881132217 | 0 | 0 |
| BRD-A84481105__thioridazine__trt_cp | 531 | 0.015208395 | 0.812602888 | 0.887528881 | 0 | 0 |
| BRD-K54094468__remoxipride__trt_cp | 72 | 0.016437684 | 0.81336655 | 0.890370642 | 0 | 0 |
| BRD-K82562631__tolmetin__trt_cp | 91 | 0.011627876 | 0.813504113 | 0.890883313 | 0 | 0 |
| BRD-K52756523__dirithromycin__trt_cp | 12 | 0.035597126 | 0.814364521 | 0.8940952 | 0 | 0 |
| BRD-K02113016__olaparib__trt_poscon | 54 | 0.023827647 | 0.814475969 | 0.894511906 | 0 | 0 |
| BRD-A28746609__paclitaxel__trt_cp | 83 | 0.019334721 | 0.814634894 | 0.895106403 | 0 | 0 |
| BRD-A97437073__rosiglitazone__trt_cp | 346 | 0.012280373 | 0.815352317 | 0.897794036 | 0 | 0 |
| BRD-A18419789__etoposide__trt_cp | 51 | 0.036134728 | 0.818481936 | 0.909595016 | 0 | 0 |
| BRD-A55424491__methotrexate__trt_cp | 73 | 0.022440237 | 0.820814652 | 0.918474133 | 0 | 0 |
| BRD-K52256627__chlorhexidine__trt_cp | 26 | 0.031155719 | 0.823083366 | 0.927179649 | 0 | 0 |
| BRD-A43671941__oxprenolol__trt_cp | 63 | 0.014702261 | 0.825401287 | 0.936147156 | 0 | 0 |
| BRD-K19456237__piracetam__trt_cp | 12 | 0.036638654 | 0.825751114 | 0.937507103 | 0 | 0 |
| BRD-K73589491__nizatidine__trt_cp | 99 | 0.013117441 | 0.826164712 | 0.939117197 | 0 | 0 |
| BRD-K15933101__ropinirole__trt_cp | 89 | 0.016577511 | 0.826302145 | 0.93965275 | 0 | 0 |
| BRD-K50128260__sildenafil__trt_cp | 278 | 0.019140806 | 0.827023385 | 0.942467721 | 0 | 0 |
| BRD-K05510762__granisetron__trt_cp | 10 | 0.041034074 | 0.827534874 | 0.944468576 | 0 | 0 |
| BRD-A60217728__iohexol__trt_cp | 12 | 0.035326022 | 0.833110308 | 0.966529317 | 0 | 0 |
| BRD-K14888893__minoxidil__trt_cp | 225 | 0.01986009 | 0.833370013 | 0.967568382 | 0 | 0 |
| BRD-K27184429__levocetirizine__trt_cp | 37 | 0.022517132 | 0.833768856 | 0.96916617 | 0 | 0 |
| BRD-A02579693__etoposide__trt_cp | 24 | 0.062183797 | 0.834349376 | 0.971496192 | 0 | 0 |
| BRD-K75649340__tioguanine__trt_cp | 10 | 0.051738842 | 0.834775996 | 0.973211879 | 0 | 0 |
| BRD-A49160188__donepezil__trt_cp | 119 | 0.014409036 | 0.835528551 | 0.976245343 | 0 | 0 |
| BRD-K46018455__bezafibrate__trt_cp | 93 | 0.013305037 | 0.836349173 | 0.97956345 | 0 | 0 |
| BRD-A21490874__fluoxymesterone__trt_cp | 26 | 0.022840733 | 0.837159246 | 0.982849514 | 0 | 0 |
| BRD-K77947974__fluspirilene__trt_cp | 276 | 0.018495339 | 0.837455594 | 0.984054305 | 0 | 0 |
| BRD-K86595100__chlordiazepoxide__trt_cp | 47 | 0.018793787 | 0.83764192 | 0.984812539 | 0 | 0 |
| BRD-A07440155__labetalol__trt_cp | 138 | 0.011457293 | 0.838152803 | 0.986894433 | 0 | 0 |
| BRD-K30020243__aliskiren__trt_cp | 83 | 0.014326662 | 0.838474108 | 0.988205972 | 0 | 0 |
| BRD-K14791739__fluticasone__trt_cp | 64 | 0.020718262 | 0.839523483 | 0.992501323 | 0 | 0 |
| BRD-K32107296__temozolomide__trt_cp | 346 | 0.014544865 | 0.841314184 | 0.999873701 | 0 | 0 |
| BRD-K17113870__lovastatin__trt_cp | 12 | 0.04860185 | 0.844932822 | 1.014940156 | 0 | 0 |
| BRD-A75479906__rimantadine__trt_cp | 45 | 0.018697931 | 0.84515137 | 1.015857481 | 0 | 0 |
| BRD-K59935627__ceftazidime__trt_cp | 11 | 0.045075808 | 0.848039106 | 1.02805961 | 0 | 0 |
| BRD-K14204120__ranitidine__trt_cp | 63 | 0.018383316 | 0.850159411 | 1.037117335 | 0 | 0 |
| BRD-K11439261__dydrogesterone__trt_cp | 12 | 0.039724012 | 0.850355873 | 1.037960911 | 0 | 0 |
| BRD-K49481516__galantamine__trt_cp | 49 | 0.020545825 | 0.851220794 | 1.041683564 | 0 | 0 |
| BRD-U57440914__lansoprazole__trt_cp | 23 | 0.035678518 | 0.851740821 | 1.043928746 | 0 | 0 |
| BRD-K52989797__clomipramine__trt_cp | 117 | 0.015327145 | 0.852540684 | 1.047392407 | 0 | 0 |
| BRD-K56343971__vemurafenib__trt_cp | 413 | 0.022677373 | 0.853389909 | 1.051083631 | 0 | 0 |
| BRD-K76205745__losartan__trt_cp | 243 | 0.013400658 | 0.85621516 | 1.063468196 | 0 | 0 |
| BRD-K09471561__levofloxacin__trt_cp | 37 | 0.02506849 | 0.856670759 | 1.065480638 | 0 | 0 |
| BRD-A47494775__dipivefrine__trt_cp | 65 | 0.019959297 | 0.857287602 | 1.068212214 | 0 | 0 |
| BRD-A21858158__praziquantel__trt_cp | 91 | 0.022600751 | 0.859740519 | 1.079154271 | 0 | 0 |
| BRD-A51820102__econazole__trt_cp | 63 | 0.023460889 | 0.859802418 | 1.079432064 | 0 | 0 |
| BRD-A69951442__dexamethasone__trt_cp | 172 | 0.0210887 | 0.860742254 | 1.083660191 | 0 | 0 |
| BRD-K97564742__mepyramine__trt_cp | 72 | 0.016371184 | 0.861351068 | 1.086409493 | 0 | 0 |
| BRD-K67966701__lisinopril__trt_cp | 10 | 0.042342214 | 0.864693862 | 1.101653645 | 0 | 0 |
| BRD-A49765801__fludroxycortide__trt_cp | 63 | 0.022880382 | 0.864753186 | 1.101926498 | 0 | 0 |
| BRD-A25143711__hydrocortisone__trt_cp | 63 | 0.021328398 | 0.864924347 | 1.102714183 | 0 | 0 |
| BRD-K91328526__clomifene__trt_cp | 10 | 0.053327173 | 0.866991253 | 1.112280666 | 0 | 0 |
| BRD-K53987533__fluphenazine__trt_cp | 63 | 0.026204828 | 0.867807786 | 1.116088092 | 0 | 0 |
| BRD-K26801045__pipamperone__trt_cp | 80 | 0.022456629 | 0.86822396 | 1.118034918 | 0 | 0 |
| BRD-K74913225__brinzolamide__trt_cp | 72 | 0.016943854 | 0.868392665 | 1.118825312 | 0 | 0 |
| BRD-A59198242__viloxazine__trt_cp | 11 | 0.03933849 | 0.871089543 | 1.131556553 | 0 | 0 |
| BRD-A56470328__fludrocortisone__trt_cp | 10 | 0.050300614 | 0.871716874 | 1.134544422 | 0 | 0 |
| BRD-K47244735__progesterone__trt_cp | 12 | 0.042649822 | 0.872442631 | 1.138013755 | 0 | 0 |
| BRD-A13133631__fluorometholone__trt_cp | 52 | 0.030107436 | 0.872797511 | 1.139715187 | 0 | 0 |
| BRD-K79254416__decitabine__trt_cp | 109 | 0.018257942 | 0.873643372 | 1.143783932 | 0 | 0 |
| BRD-A38161572__atovaquone__trt_cp | 12 | 0.044702501 | 0.877873553 | 1.164422349 | 0 | 0 |
| BRD-K51677086__erythromycin__trt_cp | 84 | 0.024215912 | 0.878799684 | 1.169007444 | 0 | 0 |
| BRD-K48470486__cefmetazole__trt_cp | 12 | 0.040513753 | 0.878964244 | 1.169824724 | 0 | 0 |
| BRD-K44276885__acarbose__trt_cp | 81 | 0.020194267 | 0.878989097 | 1.169948224 | 0 | 0 |
| BRD-K55127134__fluphenazine__trt_cp | 311 | 0.016823941 | 0.879823138 | 1.174103119 | 0 | 0 |
| BRD-K06388322__pramipexole__trt_cp | 84 | 0.020245292 | 0.880185254 | 1.175913381 | 0 | 0 |
| BRD-K28183345__proguanil__trt_cp | 74 | 0.02004662 | 0.880777656 | 1.178883201 | 0 | 0 |
| BRD-K81774264__flumethasone__trt_cp | 11 | 0.050452304 | 0.882311661 | 1.186622191 | 0 | 0 |
| BRD-K39621635__artemether__trt_cp | 34 | 0.027890587 | 0.88241461 | 1.187144114 | 0 | 0 |
| BRD-K82941592__rosuvastatin__trt_cp | 83 | 0.024781708 | 0.882530128 | 1.187730143 | 0 | 0 |
| BRD-K95391106__prednicarbate__trt_cp | 11 | 0.047234891 | 0.88326282 | 1.191456662 | 0 | 0 |
| BRD-K72029282__probucol__trt_cp | 76 | 0.020154999 | 0.884064461 | 1.195552909 | 0 | 0 |
| BRD-K23204545__busulfan__trt_cp | 81 | 0.022483172 | 0.884570695 | 1.198150041 | 0 | 0 |
| BRD-K44779798__miglitol__trt_cp | 46 | 0.026950124 | 0.886228069 | 1.206709958 | 0 | 0 |
| BRD-A78303415__hyoscyamine__trt_cp | 53 | 0.022670948 | 0.887494956 | 1.213313229 | 0 | 0 |
| BRD-K08681769__ibandronic-acid__trt_cp | 12 | 0.048852702 | 0.88759241 | 1.213823369 | 0 | 0 |
| BRD-K10961822__latanoprost__trt_cp | 37 | 0.033091901 | 0.887756122 | 1.214681067 | 0 | 0 |
| BRD-K56851771__enzalutamide__trt_cp | 189 | 0.017129857 | 0.889377966 | 1.223226742 | 0 | 0 |
| BRD-K79092138__nitrofural__trt_cp | 52 | 0.031402768 | 0.889477522 | 1.22375423 | 0 | 0 |
| BRD-A65671304__candesartan__trt_cp | 83 | 0.019031637 | 0.890819796 | 1.230899623 | 0 | 0 |
| BRD-K60507178__podophyllotoxin__trt_cp | 12 | 0.05846395 | 0.891175277 | 1.232802533 | 0 | 0 |
| BRD-A92537424__danazol__trt_cp | 85 | 0.022893803 | 0.89129487 | 1.233443723 | 0 | 0 |
| BRD-K02607075__tubocurarine__trt_cp | 66 | 0.024526168 | 0.891805149 | 1.236185275 | 0 | 0 |
| BRD-A07000685__hydrocortisone__trt_cp | 52 | 0.02984346 | 0.892060914 | 1.237562913 | 0 | 0 |
| BRD-K41410256__balsalazide__trt_cp | 46 | 0.029669286 | 0.89297698 | 1.242516543 | 0 | 0 |
| BRD-K86940322__perindopril__trt_cp | 11 | 0.046810853 | 0.894460509 | 1.250604023 | 0 | 0 |
| BRD-K56231354__triprolidine__trt_cp | 52 | 0.023018386 | 0.895315337 | 1.255301514 | 0 | 0 |
| BRD-A13946108__sulindac__trt_cp | 68 | 0.019474524 | 0.896020881 | 1.259199621 | 0 | 0 |
| BRD-K35708212__ouabain__trt_cp | 62 | 0.05934864 | 0.89638318 | 1.26120877 | 0 | 0 |
| BRD-A31836915__cyanocobalamin__trt_cp | 12 | 0.050727708 | 0.89657008 | 1.262247226 | 0 | 0 |
| BRD-A78322124__dobutamine__trt_cp | 74 | 0.021505726 | 0.899011392 | 1.275938626 | 0 | 0 |
| BRD-K28029915__dolasetron__trt_cp | 59 | 0.021624123 | 0.899680991 | 1.279735943 | 0 | 0 |
| BRD-K93332168__isocarboxazid__trt_cp | 77 | 0.020418067 | 0.900389086 | 1.283771756 | 0 | 0 |
| BRD-K15014948__tranexamic-acid__trt_cp | 10 | 0.054623876 | 0.900480361 | 1.284293507 | 0 | 0 |
| BRD-K02130563__panobinostat__trt_cp | 680 | 0.059182324 | 0.900552279 | 1.284704853 | 0 | 0 |
| BRD-A59303141__quinethazone__trt_cp | 64 | 0.021145739 | 0.900888719 | 1.286632064 | 0 | 0 |
| BRD-K90027355__spironolactone__trt_cp | 113 | 0.018444676 | 0.901033262 | 1.287461515 | 0 | 0 |
| BRD-A53077924__tianeptine__trt_cp | 53 | 0.029026352 | 0.901382776 | 1.289470841 | 0 | 0 |
| BRD-A73368467__fexofenadine__trt_cp | 94 | 0.018373373 | 0.9018741 | 1.292304254 | 0 | 0 |
| BRD-K81091703__chlorprothixene__trt_cp | 8 | 0.061964174 | 0.902209517 | 1.294244544 | 0 | 0 |
| BRD-K88172511__naltrexone__trt_cp | 104 | 0.017360623 | 0.902505776 | 1.295962372 | 0 | 0 |
| BRD-K95785345__thiamine__trt_cp | 12 | 0.049267697 | 0.903144326 | 1.299678016 | 0 | 0 |
| BRD-K72903603__zidovudine__trt_cp | 54 | 0.021728045 | 0.904071407 | 1.305104738 | 0 | 0 |
| BRD-K92310201__mometasone__trt_cp | 12 | 0.04864278 | 0.90464638 | 1.308489774 | 0 | 0 |
| BRD-A93236127__digitoxin__trt_cp | 74 | 0.07453045 | 0.905303251 | 1.312375417 | 0 | 0 |
| BRD-K54759182__dosulepin__trt_cp | 68 | 0.021251894 | 0.907294146 | 1.32427507 | 0 | 0 |
| BRD-K99257182__quinidine__trt_cp | 10 | 0.052208959 | 0.907314122 | 1.324395417 | 0 | 0 |
| BRD-A02367930__ethinyl-estradiol__trt_cp | 52 | 0.028437495 | 0.908155561 | 1.329482372 | 0 | 0 |
| BRD-K57174586__sertraline__trt_cp | 11 | 0.050329864 | 0.908839512 | 1.333642717 | 0 | 0 |
| BRD-K28494619__sulfamethoxazole__trt_cp | 10 | 0.062802426 | 0.909565047 | 1.338081394 | 0 | 0 |
| BRD-K48099440__prostaglandin-e1__trt_cp | 11 | 0.051740568 | 0.910130505 | 1.341559118 | 0 | 0 |
| BRD-K37798499__etoposide__trt_cp | 35 | 0.04881521 | 0.910374101 | 1.343062312 | 0 | 0 |
| BRD-K67100011__pivmecillinam__trt_cp | 84 | 0.023128567 | 0.910484986 | 1.343747574 | 0 | 0 |
| BRD-K26657438__imiquimod__trt_cp | 191 | 0.015120788 | 0.910932721 | 1.346520986 | 0 | 0 |
| BRD-K22134346__simvastatin__trt_cp | 310 | 0.019723488 | 0.911487253 | 1.349970351 | 0 | 0 |
| BRD-A79237180__ascorbic-acid__trt_cp | 45 | 0.032302039 | 0.913038337 | 1.3597049 | 0 | 0 |
| BRD-K05658747__raltegravir__trt_cp | 61 | 0.026030259 | 0.913595231 | 1.363231581 | 0 | 0 |
| BRD-A22032524__amlodipine__trt_cp | 93 | 0.03168126 | 0.913907819 | 1.365218572 | 0 | 0 |
| BRD-K27721098__clopidogrel__trt_cp | 41 | 0.05472238 | 0.915024155 | 1.372359054 | 0 | 0 |
| BRD-K33127281__adapalene__trt_cp | 22 | 0.04741377 | 0.91522379 | 1.37364339 | 0 | 0 |
| BRD-K96471533__nitazoxanide__trt_cp | 37 | 0.039166628 | 0.916473443 | 1.381734904 | 0 | 0 |
| BRD-K48970916__danazol__trt_cp | 114 | 0.023171929 | 0.916879853 | 1.384386001 | 0 | 0 |
| BRD-K82036761__sertraline__trt_cp | 119 | 0.034093099 | 0.91723482 | 1.38670951 | 0 | 0 |
| BRD-K55191674__benzylpenicillin__trt_cp | 78 | 0.029514003 | 0.917881972 | 1.39096495 | 0 | 0 |
| BRD-K36616567__doxepin__trt_cp | 90 | 0.021654691 | 0.918026209 | 1.391916842 | 0 | 0 |
| BRD-K93433262__alfacalcidol__trt_cp | 45 | 0.031577575 | 0.918441328 | 1.394663474 | 0 | 0 |
| BRD-A79067928__etoposide__trt_cp | 22 | 0.062330369 | 0.918677968 | 1.396233922 | 0 | 0 |
| BRD-A75172220__hydrocortisone__trt_cp | 62 | 0.024931298 | 0.918794479 | 1.397008408 | 0 | 0 |
| BRD-K52431530__josamycin__trt_cp | 10 | 0.064124861 | 0.91916225 | 1.399458613 | 0 | 0 |
| BRD-A69512159__carbidopa__trt_cp | 101 | 0.026209058 | 0.919597706 | 1.402370666 | 0 | 0 |
| BRD-M34926192__guanadrel__trt_cp | 12 | 0.049287949 | 0.919774375 | 1.403555516 | 0 | 0 |
| BRD-A94494639__chlorpromazine__trt_cp | 24 | 0.035805347 | 0.920907363 | 1.411201276 | 0 | 0 |
| BRD-A68930007__ouabain__trt_cp | 71 | 0.073465906 | 0.922016754 | 1.418768596 | 0 | 0 |
| BRD-K32821942__azathioprine__trt_cp | 139 | 0.023041447 | 0.922794214 | 1.424120572 | 0 | 0 |
| BRD-K93460210__lamotrigine__trt_cp | 102 | 0.022033191 | 0.922997688 | 1.425528031 | 0 | 0 |
| BRD-A04352665__maraviroc__trt_cp | 173 | 0.022124811 | 0.923561321 | 1.429441567 | 0 | 0 |
| BRD-K50398167__meclofenamic-acid__trt_cp | 117 | 0.020390916 | 0.924141587 | 1.433493605 | 0 | 0 |
| BRD-K63343048__orlistat__trt_cp | 37 | 0.041505596 | 0.92425189 | 1.434266525 | 0 | 0 |
| BRD-A20126139__medrysone__trt_cp | 64 | 0.026420471 | 0.924415011 | 1.435411126 | 0 | 0 |
| BRD-K51350053__toremifene__trt_cp | 131 | 0.024643301 | 0.924629838 | 1.436921413 | 0 | 0 |
| BRD-A07893380__vigabatrin__trt_cp | 12 | 0.05360859 | 0.924761745 | 1.437850386 | 0 | 0 |
| BRD-K51313569__palbociclib__trt_cp | 245 | 0.038720275 | 0.924834861 | 1.438365846 | 0 | 0 |
| BRD-K10481964__mestranol__trt_cp | 11 | 0.05328281 | 0.925352304 | 1.442024751 | 0 | 0 |
| BRD-K73978287__hydrocortisone__trt_cp | 45 | 0.028932955 | 0.925497878 | 1.443057607 | 0 | 0 |
| BRD-K30697463__desoximetasone__trt_cp | 46 | 0.029498406 | 0.925600995 | 1.443790166 | 0 | 0 |
| BRD-K88365400__viomycin__trt_cp | 11 | 0.060112062 | 0.925759406 | 1.444917045 | 0 | 0 |
| BRD-A56359832__zileuton__trt_cp | 214 | 0.016940625 | 0.928563787 | 1.465177759 | 0 | 0 |
| BRD-A49225603__alimemazine__trt_cp | 51 | 0.037513963 | 0.929477147 | 1.471908064 | 0 | 0 |
| BRD-A01643550__prednisolone__trt_cp | 80 | 0.026285011 | 0.930281736 | 1.477892599 | 0 | 0 |
| BRD-K43860855__iobenguane__trt_cp | 75 | 0.02696065 | 0.932534102 | 1.494933512 | 0 | 0 |
| BRD-K32977963__eugenol__trt_cp | 53 | 0.03233227 | 0.932753209 | 1.496614548 | 0 | 0 |
| BRD-K38775274__dexamethasone__trt_cp | 133 | 0.030416207 | 0.933487428 | 1.502278709 | 0 | 0 |
| BRD-M09350392__debrisoquine__trt_cp | 10 | 0.070567225 | 0.934147743 | 1.507414225 | 0 | 0 |
| BRD-K30563334__rifabutin__trt_cp | 37 | 0.036049908 | 0.935589292 | 1.518765963 | 0 | 0 |
| BRD-K61327230__pralidoxime__trt_cp | 11 | 0.058276206 | 0.936202783 | 1.52365693 | 0 | 0 |
| BRD-K17084514__diethylstilbestrol__trt_cp | 12 | 0.073314834 | 0.936910707 | 1.529346427 | 0 | 0 |
| BRD-K57943804__equilin__trt_cp | 10 | 0.057131304 | 0.937130356 | 1.531121818 | 0 | 0 |
| BRD-K63550407__erythromycin__trt_cp | 96 | 0.024130602 | 0.938946533 | 1.545990206 | 0 | 0 |
| BRD-K70158168__saquinavir__trt_cp | 11 | 0.066529993 | 0.940512136 | 1.559087265 | 0 | 0 |
| BRD-A63445921__nicardipine__trt_cp | 86 | 0.029262531 | 0.941293001 | 1.565720785 | 0 | 0 |
| BRD-K46652470__nizatidine__trt_cp | 60 | 0.037489917 | 0.941379797 | 1.566462393 | 0 | 0 |
| BRD-A26334849__propafenone__trt_cp | 164 | 0.022800276 | 0.942111334 | 1.572747343 | 0 | 0 |
| BRD-K83597974__pargyline__trt_cp | 75 | 0.025095974 | 0.942643651 | 1.577360072 | 0 | 0 |
| BRD-K12343256__trametinib__trt_cp | 133 | 0.047901185 | 0.943025166 | 1.580686796 | 0 | 0 |
| BRD-M64432851__sunitinib__trt_cp | 66 | 0.057909938 | 0.94302644 | 1.580697936 | 0 | 0 |
| BRD-K46156294__tyloxapol__trt_cp | 11 | 0.071169807 | 0.94304968 | 1.580901158 | 0 | 0 |
| BRD-K32480324__reboxetine__trt_cp | 12 | 0.060141287 | 0.944294762 | 1.591885317 | 0 | 0 |
| BRD-K45995181__auranofin__trt_cp | 3 | 0.139222394 | 0.944597349 | 1.594583962 | 0 | 0 |
| BRD-K16508793__diazepam__trt_cp | 70 | 0.026977846 | 0.944693747 | 1.595446134 | 0 | 0 |
| BRD-K77695569__tiabendazole__trt_cp | 119 | 0.019773611 | 0.946096191 | 1.608125857 | 0 | 0 |
| BRD-K55070890__thiothixene__trt_cp | 43 | 0.062888463 | 0.946187748 | 1.608962675 | 0 | 0 |
| BRD-K78712176__carbidopa__trt_cp | 12 | 0.07083678 | 0.946259151 | 1.609616069 | 0 | 0 |
| BRD-K64994968__progesterone__trt_cp | 122 | 0.023366962 | 0.947276004 | 1.618996616 | 0 | 0 |
| BRD-K81601344__rifapentine__trt_cp | 12 | 0.085378609 | 0.947752352 | 1.623440388 | 0 | 0 |
| BRD-A69960130__bromocriptine__trt_cp | 66 | 0.035194624 | 0.947777884 | 1.623679483 | 0 | 0 |
| BRD-K88682005__chlorothiazide__trt_cp | 10 | 0.060473691 | 0.948176376 | 1.627423201 | 0 | 0 |
| BRD-K76133116__benzydamine__trt_cp | 71 | 0.030865741 | 0.949571992 | 1.640717761 | 0 | 0 |
| BRD-K01507359__rifampicin__trt_cp | 133 | 0.022539232 | 0.949610212 | 1.641085942 | 0 | 0 |
| BRD-A04756508__norgestimate__trt_cp | 81 | 0.033510678 | 0.950143869 | 1.646250181 | 0 | 0 |
| BRD-K88538023__oxiconazole__trt_cp | 12 | 0.073562112 | 0.950909417 | 1.653735996 | 0 | 0 |
| BRD-A64297288__amlodipine__trt_cp | 34 | 0.043126093 | 0.951228667 | 1.656885302 | 0 | 0 |
| BRD-K70504303__prednisolone__trt_cp | 10 | 0.063842423 | 0.951349549 | 1.658082071 | 0 | 0 |
| BRD-K97810537__beclometasone__trt_cp | 110 | 0.023780516 | 0.951447819 | 1.659056727 | 0 | 0 |
| BRD-K21815137__cefotiam__trt_cp | 12 | 0.068173479 | 0.951613425 | 1.660702803 | 0 | 0 |
| BRD-K22947005__dexbrompheniramine__trt_cp | 37 | 0.043862448 | 0.951882803 | 1.663389993 | 0 | 0 |
| BRD-K75466013__benzocaine__trt_cp | 12 | 0.065938278 | 0.952212472 | 1.666695059 | 0 | 0 |
| BRD-K60237333__niacin__trt_cp | 35 | 0.061821333 | 0.952324075 | 1.667818054 | 0 | 0 |
| BRD-K34730807__levodopa__trt_cp | 10 | 0.075621394 | 0.952765763 | 1.672283284 | 0 | 0 |
| BRD-K77554836__triamcinolone__trt_cp | 64 | 0.0305937 | 0.952878286 | 1.673426181 | 0 | 0 |
| BRD-K46137903__prednicarbate__trt_cp | 77 | 0.031538936 | 0.952964477 | 1.674303099 | 0 | 0 |
| BRD-A35588707__teniposide__trt_cp | 347 | 0.058685661 | 0.953109324 | 1.675779695 | 0 | 0 |
| BRD-K88701661__dimercaptosuccinic-acid__trt_cp | 36 | 0.0336467 | 0.953116994 | 1.675857994 | 0 | 0 |
| BRD-K42125900__docetaxel__trt_cp | 64 | 0.039211885 | 0.953745931 | 1.682313123 | 0 | 0 |
| BRD-A59174698__ritodrine__trt_cp | 60 | 0.039335243 | 0.953948843 | 1.684410752 | 0 | 0 |
| BRD-K74112339__acetohydroxamic-acid__trt_cp | 33 | 0.035312361 | 0.955004478 | 1.695444953 | 0 | 0 |
| BRD-K31627533__rimexolone__trt_cp | 65 | 0.029058784 | 0.955009286 | 1.695495684 | 0 | 0 |
| BRD-K00824317__fludroxycortide__trt_cp | 62 | 0.033721277 | 0.955095022 | 1.696401043 | 0 | 0 |
| BRD-A18917088__estradiol__trt_cp | 82 | 0.036247977 | 0.955242639 | 1.697963124 | 0 | 0 |
| BRD-A30205217__ethotoin__trt_cp | 65 | 0.030006386 | 0.955620328 | 1.70197879 | 0 | 0 |
| BRD-K22031190__diflunisal__trt_cp | 131 | 0.027072497 | 0.956034787 | 1.706417223 | 0 | 0 |
| BRD-K00312224__PPT__trt_cp | 63 | 0.042265031 | 0.956209852 | 1.708302127 | 0 | 0 |
| BRD-K43457670__acetazolamide__trt_cp | 10 | 0.07359266 | 0.956465322 | 1.711063695 | 0 | 0 |
| BRD-K86434416__selegiline__trt_cp | 137 | 0.021029028 | 0.956740694 | 1.714055074 | 0 | 0 |
| BRD-A08027849__deoxycholic-acid__trt_cp | 23 | 0.065348093 | 0.957059972 | 1.717542724 | 0 | 0 |
| BRD-K92977333__entacapone__trt_cp | 11 | 0.073468874 | 0.957955387 | 1.727436913 | 0 | 0 |
| BRD-K34411947__streptozotocin__trt_cp | 11 | 0.080519984 | 0.958223608 | 1.730433888 | 0 | 0 |
| BRD-A28856712__tetryzoline__trt_cp | 65 | 0.029773966 | 0.958423206 | 1.732674225 | 0 | 0 |
| BRD-K78692225__leflunomide__trt_cp | 81 | 0.044276365 | 0.959286826 | 1.742469357 | 0 | 0 |
| BRD-A09722536__cyclophosphamide__trt_cp | 128 | 0.027392851 | 0.95994898 | 1.750094321 | 0 | 0 |
| BRD-K49384696__lincomycin__trt_cp | 10 | 0.062373562 | 0.960856387 | 1.760711613 | 0 | 0 |
| BRD-K92073408__norethisterone__trt_cp | 158 | 0.027767014 | 0.96171383 | 1.77093002 | 0 | 0 |
| BRD-A37052580__physostigmine__trt_cp | 35 | 0.048982555 | 0.961788193 | 1.771824981 | 0 | 0 |
| BRD-K06980535__promazine__trt_cp | 262 | 0.034298414 | 0.962187701 | 1.776657562 | 0 | 0 |
| BRD-A63043573__cabergoline__trt_cp | 67 | 0.030119602 | 0.962454925 | 1.779913308 | 0 | 0 |
| BRD-K10995081__perphenazine__trt_cp | 137 | 0.035727942 | 0.962775891 | 1.783848924 | 0 | 0 |
| BRD-K66198023__losartan__trt_cp | 37 | 0.045479825 | 0.963295833 | 1.790283584 | 0 | 0 |
| BRD-A29734509__disopyramide__trt_cp | 138 | 0.02068284 | 0.9644714 | 1.805111728 | 0 | 0 |
| BRD-K88043978__amikacin__trt_cp | 10 | 0.081109024 | 0.964535464 | 1.805931298 | 0 | 0 |
| BRD-A09533288__verapamil__trt_cp | 303 | 0.015809064 | 0.964989035 | 1.811768788 | 0 | 0 |
| BRD-K10974103__diloxanide__trt_cp | 94 | 0.02683955 | 0.965560069 | 1.819206959 | 0 | 0 |
| BRD-K81528515__nilotinib__trt_cp | 359 | 0.024531313 | 0.96573456 | 1.82150005 | 0 | 0 |
| BRD-K67637637__olopatadine__trt_cp | 48 | 0.045337225 | 0.966083555 | 1.826115339 | 0 | 0 |
| BRD-K01188359__vinblastine__trt_cp | 35 | 0.107382948 | 0.966531557 | 1.83209757 | 0 | 0 |
| BRD-M10279501__gentamicin__trt_cp | 10 | 0.085648556 | 0.96674487 | 1.834969158 | 0 | 0 |
| BRD-A68888262__azelastine__trt_cp | 49 | 0.043311994 | 0.967417315 | 1.844121943 | 0 | 0 |
| BRD-A62421304__mepenzolate__trt_cp | 12 | 0.060941579 | 0.967726619 | 1.848384345 | 0 | 0 |
| BRD-A34309505__zopiclone__trt_cp | 12 | 0.076623814 | 0.967871913 | 1.850398229 | 0 | 0 |
| BRD-K99792991__hexachlorophene__trt_cp | 23 | 0.076567565 | 0.968039121 | 1.852725193 | 0 | 0 |
| BRD-K53790871__triamcinolone__trt_cp | 43 | 0.040246405 | 0.968395602 | 1.857719942 | 0 | 0 |
| BRD-A16478930__amcinonide__trt_cp | 42 | 0.039243229 | 0.968723457 | 1.862354868 | 0 | 0 |
| BRD-K46556543__canrenoic-acid__trt_cp | 63 | 0.053290839 | 0.969282005 | 1.870344532 | 0 | 0 |
| BRD-K76775527__nimesulide__trt_cp | 76 | 0.032404619 | 0.969445143 | 1.872700798 | 0 | 0 |
| BRD-K86284654__alfacalcidol__trt_cp | 12 | 0.093739257 | 0.969595705 | 1.874884679 | 0 | 0 |
| BRD-K09416995__lovastatin__trt_cp | 181 | 0.035114936 | 0.969739924 | 1.876984983 | 0 | 0 |
| BRD-K69608737__tacrolimus__trt_cp | 82 | 0.039256281 | 0.969834015 | 1.878359739 | 0 | 0 |
| BRD-A10715913__sulpiride__trt_cp | 49 | 0.06367079 | 0.96985183 | 1.878620427 | 0 | 0 |
| BRD-A58048407__nimodipine__trt_cp | 289 | 0.021152881 | 0.970192992 | 1.883637573 | 0 | 0 |
| BRD-K97158071__droperidol__trt_cp | 120 | 0.02550783 | 0.971634235 | 1.90537356 | 0 | 0 |
| BRD-K29950728__clomifene__trt_cp | 129 | 0.038912405 | 0.971665378 | 1.905853285 | 0 | 0 |
| BRD-A34462049__tigecycline__trt_cp | 385 | 0.037244997 | 0.972882245 | 1.924950834 | 0 | 0 |
| BRD-K06319591__ceforanide__trt_cp | 12 | 0.084321169 | 0.973347093 | 1.932435185 | 0 | 0 |
| BRD-K63750851__mycophenolic-acid__trt_cp | 31 | 0.093445881 | 0.97362186 | 1.936910543 | 0 | 0 |
| BRD-K08619574__thioproperazine__trt_cp | 72 | 0.039360527 | 0.973664717 | 1.93761209 | 0 | 0 |
| BRD-K13927029__retinol__trt_cp | 85 | 0.050248543 | 0.973981331 | 1.942824731 | 0 | 0 |
| BRD-K21401044__danazol__trt_cp | 10 | 0.077564049 | 0.974673869 | 1.954414123 | 0 | 0 |
| BRD-A62326914__paroxetine__trt_cp | 45 | 0.047366669 | 0.974777551 | 1.956171983 | 0 | 0 |
| BRD-A81233518__glycopyrrolate__trt_cp | 94 | 0.027297842 | 0.97493066 | 1.958778949 | 0 | 0 |
| BRD-A82371568__clofarabine__trt_cp | 69 | 0.06073142 | 0.975636099 | 1.970965684 | 0 | 0 |
| BRD-A82238138__budesonide__trt_cp | 52 | 0.040060827 | 0.975822972 | 1.974243554 | 0 | 0 |
| BRD-K42870217__imipenem__trt_cp | 12 | 0.093769805 | 0.975829686 | 1.974361723 | 0 | 0 |
| BRD-K23478508__digoxin__trt_cp | 60 | 0.105482166 | 0.976237052 | 1.981583319 | 0 | 0 |
| BRD-K80396088__gliquidone__trt_cp | 77 | 0.032587771 | 0.976426054 | 1.984969252 | 0 | 0 |
| BRD-K08206212__entecavir__trt_cp | 63 | 0.040086509 | 0.977002605 | 1.995441123 | 0 | 0 |
| BRD-A75144621__digoxin__trt_cp | 39 | 0.10981613 | 0.977639198 | 2.007263589 | 0 | 0 |
| BRD-K10843433__phenylbutazone__trt_cp | 74 | 0.031827275 | 0.977876219 | 2.011737999 | 0 | 0 |
| BRD-K18574842__nafcillin__trt_cp | 149 | 0.030772202 | 0.97794132 | 2.01297404 | 0 | 0 |
| BRD-K44495371__reserpine__trt_cp | 11 | 0.084075476 | 0.978069221 | 2.015411427 | 0 | 0 |
| BRD-A48570745__ivermectin__trt_cp | 58 | 0.060615984 | 0.978985734 | 2.033237527 | 0 | 0 |
| BRD-K33396764__alpha-linolenic-acid__trt_cp | 94 | 0.032963494 | 0.978998325 | 2.033486948 | 0 | 0 |
| BRD-A29485665__bicalutamide__trt_cp | 125 | 0.028410787 | 0.979071891 | 2.034946876 | 0 | 0 |
| BRD-K51207550__ethionamide__trt_cp | 12 | 0.071735013 | 0.979106501 | 2.035635221 | 0 | 0 |
| BRD-A79768653__sirolimus__trt_cp | 681 | 0.06361093 | 0.979733247 | 2.04827046 | 0 | 0 |
| BRD-M18219129__guanethidine__trt_cp | 10 | 0.094467953 | 0.979891807 | 2.051519466 | 0 | 0 |
| BRD-K32247306__primidone__trt_cp | 137 | 0.024519698 | 0.98002584 | 2.054282878 | 0 | 0 |
| BRD-M61246020__fenoprofen__trt_cp | 12 | 0.08374586 | 0.980039311 | 2.054561491 | 0 | 0 |
| BRD-K63504947__semaxanib__trt_cp | 64 | 0.048935848 | 0.980101443 | 2.055848578 | 0 | 0 |
| BRD-K16444452__ibudilast__trt_cp | 82 | 0.032159107 | 0.980581265 | 2.065904792 | 0 | 0 |
| BRD-K40645748__mefloquine__trt_cp | 45 | 0.057481339 | 0.981412234 | 2.083831124 | 0 | 0 |
| BRD-K08327065__kanamycin__trt_cp | 10 | 0.09864295 | 0.981521854 | 2.08624659 | 0 | 0 |
| BRD-K41406082__sulfapyridine__trt_cp | 12 | 0.093705476 | 0.981718597 | 2.090612592 | 0 | 0 |
| BRD-A39415247__norethisterone__trt_cp | 66 | 0.040167984 | 0.981766504 | 2.09168177 | 0 | 0 |
| BRD-K50133271__tolfenamic-acid__trt_cp | 10 | 0.085694914 | 0.981929657 | 2.095341039 | 0 | 0 |
| BRD-K47639036__flavoxate__trt_cp | 76 | 0.03342809 | 0.982093786 | 2.099050739 | 0 | 0 |
| BRD-K98763141__niflumic-acid__trt_cp | 55 | 0.031643229 | 0.982226643 | 2.102074914 | 0 | 0 |
| BRD-K89997465__chlorpromazine__trt_cp | 239 | 0.034222077 | 0.982428692 | 2.106711259 | 0 | 0 |
| BRD-K86858124__paclitaxel__trt_cp | 10 | 0.097243932 | 0.982490317 | 2.1081344 | 0 | 0 |
| BRD-K14705039__sulfathiazole__trt_cp | 10 | 0.082400417 | 0.982634163 | 2.111473032 | 0 | 0 |
| BRD-K71879491__tretinoin__trt_cp | 461 | 0.032145192 | 0.982653334 | 2.111919759 | 0 | 0 |
| BRD-A69927616__isradipine__trt_cp | 60 | 0.043031392 | 0.982888633 | 2.117437507 | 0 | 0 |
| BRD-K44094313__mifepristone__trt_cp | 8 | 0.108345119 | 0.982939508 | 2.118639049 | 0 | 0 |
| BRD-K43764301__dexketoprofen__trt_cp | 64 | 0.042183846 | 0.983017693 | 2.120491554 | 0 | 0 |
| BRD-K60762818__desipramine__trt_cp | 139 | 0.035026421 | 0.983047796 | 2.121206749 | 0 | 0 |
| BRD-K74615293__meropenem__trt_cp | 11 | 0.092154469 | 0.983448471 | 2.130831109 | 0 | 0 |
| BRD-K24053527__dextromethorphan__trt_cp | 85 | 0.033879009 | 0.983500647 | 2.132099046 | 0 | 0 |
| BRD-K29582115__ziprasidone__trt_cp | 65 | 0.044818915 | 0.983645193 | 2.135629643 | 0 | 0 |
| BRD-A41833852__naloxone__trt_cp | 61 | 0.035298723 | 0.983680667 | 2.13650019 | 0 | 0 |
| BRD-A94756469__digoxin__trt_cp | 77 | 0.111711161 | 0.984057557 | 2.14585079 | 0 | 0 |
| BRD-A14395271__mesoridazine__trt_cp | 281 | 0.027464459 | 0.984068108 | 2.146115258 | 0 | 0 |
| BRD-A74980173__gatifloxacin__trt_cp | 73 | 0.048703146 | 0.984174074 | 2.148779887 | 0 | 0 |
| BRD-K71103788__duloxetine__trt_cp | 167 | 0.031554807 | 0.984268852 | 2.151176195 | 0 | 0 |
| BRD-K81925854__clocortolone__trt_cp | 10 | 0.089556431 | 0.984319049 | 2.152450344 | 0 | 0 |
| BRD-A37630846__daunorubicin__trt_cp | 21 | 0.13325004 | 0.984347446 | 2.15317271 | 0 | 0 |
| BRD-A75726477__clenbuterol__trt_cp | 9 | 0.091391847 | 0.984489964 | 2.156815139 | 0 | 0 |
| BRD-K26739552__fenspiride__trt_cp | 10 | 0.087121808 | 0.984787874 | 2.164522765 | 0 | 0 |
| BRD-K99369265__sirolimus__trt_cp | 65 | 0.060035299 | 0.984901823 | 2.167505188 | 0 | 0 |
| BRD-K11196887__norfloxacin__trt_cp | 10 | 0.09480359 | 0.984949993 | 2.168771785 | 0 | 0 |
| BRD-K59163477__cisapride__trt_cp | 63 | 0.036714249 | 0.985275419 | 2.177421122 | 0 | 0 |
| BRD-K87156652__carbimazole__trt_cp | 12 | 0.101333735 | 0.985278778 | 2.177511238 | 0 | 0 |
| BRD-K99451608__lopinavir__trt_cp | 67 | 0.040626816 | 0.985918098 | 2.194997124 | 0 | 0 |
| BRD-K13514097__everolimus__trt_cp | 58 | 0.111662248 | 0.985999291 | 2.197266521 | 0 | 0 |
| BRD-K50590187__capsaicin__trt_cp | 190 | 0.042182733 | 0.986154892 | 2.201647536 | 0 | 0 |
| BRD-K88969189__ethinylestradiol__trt_cp | 11 | 0.09206916 | 0.986239098 | 2.204036131 | 0 | 0 |
| BRD-A44827100__erythromycin__trt_cp | 89 | 0.038449091 | 0.986552293 | 2.213032361 | 0 | 0 |
| BRD-K68867920__quetiapine__trt_cp | 119 | 0.037767855 | 0.986688229 | 2.21699341 | 0 | 0 |
| BRD-K14312455__tazobactam__trt_cp | 12 | 0.081299119 | 0.986717012 | 2.217836584 | 0 | 0 |
| BRD-K06926592__tretinoin__trt_cp | 109 | 0.050117273 | 0.986787573 | 2.219910359 | 0 | 0 |
| BRD-A75919782__leucovorin__trt_cp | 11 | 0.103248325 | 0.986920204 | 2.223834339 | 0 | 0 |
| BRD-A71390734__idarubicin__trt_cp | 283 | 0.07467572 | 0.986936893 | 2.224330536 | 0 | 0 |
| BRD-K69726342__atorvastatin__trt_cp | 35 | 0.060696044 | 0.986986282 | 2.225802171 | 0 | 0 |
| BRD-K71125014__sulfadimethoxine__trt_cp | 10 | 0.121181216 | 0.987017128 | 2.226723714 | 0 | 0 |
| BRD-A16934955__nalbuphine__trt_cp | 36 | 0.048459378 | 0.987240998 | 2.233469418 | 0 | 0 |
| BRD-K38197229__bumetanide__trt_cp | 160 | 0.033308742 | 0.98740124 | 2.238361038 | 0 | 0 |
| BRD-A85648045__vinblastine__trt_cp | 26 | 0.101548665 | 0.987806708 | 2.250983465 | 0 | 0 |
| BRD-A65051990__acenocoumarol__trt_cp | 12 | 0.08527752 | 0.987818654 | 2.251360855 | 0 | 0 |
| BRD-K92760278__riboflavin__trt_cp | 84 | 0.040235051 | 0.987938016 | 2.255149148 | 0 | 0 |
| BRD-K89626439__sirolimus__trt_cp | 67 | 0.067831312 | 0.988169288 | 2.262582742 | 0 | 0 |
| BRD-A70155556__lovastatin__trt_cp | 174 | 0.068041616 | 0.988382702 | 2.269555007 | 0 | 0 |
| BRD-K55106630__dienestrol__trt_cp | 10 | 0.112942726 | 0.988414603 | 2.270606747 | 0 | 0 |
| BRD-K04185004__oxybuprocaine__trt_cp | 81 | 0.03543124 | 0.988492045 | 2.273170467 | 0 | 0 |
| BRD-K62200014__anagrelide__trt_cp | 120 | 0.03157401 | 0.988702645 | 2.280218874 | 0 | 0 |
| BRD-A51182606__chloramphenicol__trt_cp | 44 | 0.065189629 | 0.98873343 | 2.281258761 | 0 | 0 |
| BRD-A51964809__halofantrine__trt_cp | 11 | 0.097302701 | 0.988777325 | 2.282745727 | 0 | 0 |
| BRD-K45033733__famciclovir__trt_cp | 128 | 0.02698606 | 0.988839393 | 2.284857007 | 0 | 0 |
| BRD-A99314711__paromomycin__trt_cp | 11 | 0.091167152 | 0.988920355 | 2.287626363 | 0 | 0 |
| BRD-K76304753__phenazopyridine__trt_cp | 77 | 0.04960151 | 0.988945687 | 2.28849647 | 0 | 0 |
| BRD-K46503544__etoposide__trt_cp | 25 | 0.094655489 | 0.989137463 | 2.295140478 | 0 | 0 |
| BRD-K99063460__didanosine__trt_cp | 47 | 0.04666536 | 0.989232538 | 2.298472272 | 0 | 0 |
| BRD-K56429665__calcipotriol__trt_cp | 90 | 0.048366377 | 0.989311694 | 2.301265795 | 0 | 0 |
| BRD-K44273375__tolnaftate__trt_cp | 10 | 0.123907876 | 0.989418887 | 2.305077628 | 0 | 0 |
| BRD-K52459643__prostaglandin-e1__trt_cp | 68 | 0.039968457 | 0.989483649 | 2.307396927 | 0 | 0 |
| BRD-K47036706__fluocinonide__trt_cp | 12 | 0.092792096 | 0.989784791 | 2.318347963 | 0 | 0 |
| BRD-K00673382__famotidine__trt_cp | 45 | 0.053670298 | 0.989897909 | 2.322534349 | 0 | 0 |
| BRD-A34299591__budesonide__trt_cp | 97 | 0.040378416 | 0.989903325 | 2.322735784 | 0 | 0 |
| BRD-K04389336__labetalol__trt_cp | 45 | 0.045675808 | 0.990065459 | 2.328810957 | 0 | 0 |
| BRD-K25394294__oxaprozin__trt_cp | 167 | 0.026700627 | 0.990118896 | 2.330832243 | 0 | 0 |
| BRD-K72222507__quinapril__trt_cp | 69 | 0.033544939 | 0.990181548 | 2.333214245 | 0 | 0 |
| BRD-K58442346__dopamine__trt_cp | 24 | 0.073391587 | 0.990353498 | 2.339820631 | 0 | 0 |
| BRD-K81709173__halcinonide__trt_cp | 67 | 0.055476155 | 0.990433235 | 2.342919134 | 0 | 0 |
| BRD-K97309399__thiothixene__trt_cp | 61 | 0.069123705 | 0.990442436 | 2.34327812 | 0 | 0 |
| BRD-K27316855__calcitriol__trt_cp | 327 | 0.037770755 | 0.990588241 | 2.349007679 | 0 | 0 |
| BRD-A62525898__prednisone__trt_cp | 79 | 0.03773356 | 0.990890805 | 2.361149373 | 0 | 0 |
| BRD-K29627430__clavulanic-acid__trt_cp | 11 | 0.087706809 | 0.990964728 | 2.364169467 | 0 | 0 |
| BRD-K32398298__alprazolam__trt_cp | 37 | 0.071760699 | 0.991021159 | 2.366489524 | 0 | 0 |
| BRD-K61250553__loperamide__trt_cp | 304 | 0.051499777 | 0.991113857 | 2.370328529 | 0 | 0 |
| BRD-K89055274__alverine__trt_cp | 72 | 0.032242423 | 0.991255188 | 2.376249658 | 0 | 0 |
| BRD-K28936863__ketotifen__trt_cp | 178 | 0.02736286 | 0.991270641 | 2.37690218 | 0 | 0 |
| BRD-A50287119__sirolimus__trt_cp | 23 | 0.101129388 | 0.991303383 | 2.378288017 | 0 | 0 |
| BRD-K28912512__nicotinamide__trt_cp | 81 | 0.051382761 | 0.991573188 | 2.389885526 | 0 | 0 |
| BRD-K74793820__vincristine__trt_cp | 27 | 0.093505273 | 0.991639186 | 2.392772007 | 0 | 0 |
| BRD-A36471396__biperiden__trt_cp | 61 | 0.0597883 | 0.991667608 | 2.394021235 | 0 | 0 |
| BRD-K31542390__mycophenolic-acid__trt_cp | 114 | 0.074233028 | 0.991675194 | 2.394355283 | 0 | 0 |
| BRD-K54472332__elvitegravir__trt_cp | 49 | 0.073487372 | 0.99169071 | 2.395039388 | 0 | 0 |
| BRD-K63630713__etacrynic-acid__trt_cp | 106 | 0.04795747 | 0.991697273 | 2.395329087 | 0 | 0 |
| BRD-K56515112__medrysone__trt_cp | 112 | 0.036565834 | 0.991945742 | 2.406447564 | 0 | 0 |
| BRD-K55044200__amoxicillin__trt_cp | 55 | 0.063278252 | 0.992238245 | 2.419929632 | 0 | 0 |
| BRD-M41783010__acamprosate__trt_cp | 49 | 0.057800116 | 0.99226488 | 2.421179414 | 0 | 0 |
| BRD-K47679368__bromfenac__trt_cp | 44 | 0.05163384 | 0.992364354 | 2.425880634 | 0 | 0 |
| BRD-A58767537__afatinib__trt_cp | 131 | 0.061043705 | 0.9925612 | 2.435344948 | 0 | 0 |
| BRD-K71799949__carbamazepine__trt_cp | 331 | 0.035690974 | 0.992602807 | 2.437373573 | 0 | 0 |
| BRD-A92651262__nalbuphine__trt_cp | 65 | 0.043125056 | 0.992631383 | 2.438772694 | 0 | 0 |
| BRD-K39716612__fluvastatin__trt_cp | 11 | 0.108271703 | 0.992801465 | 2.447200543 | 0 | 0 |
| BRD-K81029756__diltiazem__trt_cp | 72 | 0.045476145 | 0.993021837 | 2.458385557 | 0 | 0 |
| BRD-K12184470__flunarizine__trt_cp | 80 | 0.045520279 | 0.993329004 | 2.47450778 | 0 | 0 |
| BRD-A35782587__verteporfin__trt_cp | 11 | 0.12940981 | 0.993440599 | 2.480527854 | 0 | 0 |
| BRD-K27218697__rimexolone__trt_cp | 11 | 0.088781755 | 0.993449645 | 2.481019808 | 0 | 0 |
| BRD-K30618791__docetaxel__trt_cp | 11 | 0.111392111 | 0.993473195 | 2.482303351 | 0 | 0 |
| BRD-K90733503__cefalexin__trt_cp | 44 | 0.045200151 | 0.993487098 | 2.483063015 | 0 | 0 |
| BRD-K41731458__triclosan__trt_cp | 92 | 0.052599392 | 0.993498475 | 2.48368577 | 0 | 0 |
| BRD-K63641886__cefuroxime__trt_cp | 45 | 0.061151399 | 0.993999098 | 2.512091268 | 0 | 0 |
| BRD-A11263757__azacitidine__trt_cp | 9 | 0.141032928 | 0.994012931 | 2.512905565 | 0 | 0 |
| BRD-A69815203__cyclosporin-a__trt_cp | 85 | 0.072066185 | 0.994126464 | 2.519652542 | 0 | 0 |
| BRD-K51465424__ritodrine__trt_cp | 53 | 0.046856736 | 0.994330151 | 2.532052624 | 0 | 0 |
| BRD-K15567136__papaverine__trt_cp | 78 | 0.073378922 | 0.994358039 | 2.533780996 | 0 | 0 |
| BRD-K34776109__glimepiride__trt_cp | 111 | 0.036446245 | 0.994568421 | 2.547070178 | 0 | 0 |
| BRD-A01346607__flumetasone__trt_cp | 62 | 0.057302623 | 0.994729811 | 2.557578438 | 0 | 0 |
| BRD-A38749782__fludrocortisone__trt_cp | 52 | 0.06854651 | 0.994740325 | 2.558272936 | 0 | 0 |
| BRD-A25067867__benzatropine__trt_cp | 45 | 0.057512789 | 0.994921829 | 2.570460466 | 0 | 0 |
| BRD-K28849549__mesalazine__trt_cp | 47 | 0.048361135 | 0.994941326 | 2.571792607 | 0 | 0 |
| BRD-K46212057__voriconazole__trt_cp | 67 | 0.048695567 | 0.995043599 | 2.578856263 | 0 | 0 |
| BRD-K11129031__gemfibrozil__trt_cp | 176 | 0.031441434 | 0.995201974 | 2.590055269 | 0 | 0 |
| BRD-A31204924__mitotane__trt_cp | 93 | 0.043180564 | 0.995213399 | 2.590875853 | 0 | 0 |
| BRD-A55913614__primaquine__trt_cp | 93 | 0.046015023 | 0.995224199 | 2.591653142 | 0 | 0 |
| BRD-M08470251__pyrvinium__trt_cp | 8 | 0.175418526 | 0.995237354 | 2.592602037 | 0 | 0 |
| BRD-A75850590__lomefloxacin__trt_cp | 10 | 0.118470587 | 0.995238462 | 2.592682033 | 0 | 0 |
| BRD-A19195498__trimipramine__trt_cp | 94 | 0.050720151 | 0.995266885 | 2.594740708 | 0 | 0 |
| BRD-K37289225__clozapine__trt_cp | 135 | 0.044469148 | 0.995279822 | 2.595681394 | 0 | 0 |
| BRD-A72130500__demeclocycline__trt_cp | 12 | 0.108552105 | 0.995356083 | 2.601273623 | 0 | 0 |
| BRD-K02637541__celecoxib__trt_cp | 156 | 0.034589811 | 0.995384698 | 2.603393096 | 0 | 0 |
| BRD-A77722753__hydralazine__trt_cp | 63 | 0.041877205 | 0.995478653 | 2.610435809 | 0 | 0 |
| BRD-K18855837__varenicline__trt_cp | 61 | 0.053558915 | 0.995547338 | 2.615667496 | 0 | 0 |
| BRD-A65818372__ioversol__trt_cp | 11 | 0.100256669 | 0.995641742 | 2.622977006 | 0 | 0 |
| BRD-K79199004__stanozolol__trt_cp | 12 | 0.092443899 | 0.99565923 | 2.624346551 | 0 | 0 |
| BRD-K28026280__simvastatin__trt_cp | 11 | 0.118573552 | 0.995665694 | 2.624854016 | 0 | 0 |
| BRD-K33240821__plerixafor__trt_cp | 23 | 0.082267424 | 0.995699543 | 2.627522545 | 0 | 0 |
| BRD-K39330046__hyoscyamine__trt_cp | 10 | 0.100194623 | 0.995746214 | 2.631232831 | 0 | 0 |
| BRD-K51418664__mitomycin-c__trt_cp | 43 | 0.061655536 | 0.995783641 | 2.634234695 | 0 | 0 |
| BRD-A20243730__danazol__trt_cp | 67 | 0.051587096 | 0.995792338 | 2.63493565 | 0 | 0 |
| BRD-A44448661__pentobarbital__trt_cp | 62 | 0.071191562 | 0.995982554 | 2.650600032 | 0 | 0 |
| BRD-A20794617__vancomycin__trt_cp | 12 | 0.100063138 | 0.99603518 | 2.655051118 | 0 | 0 |
| BRD-K35960502__niclosamide__trt_cp | 435 | 0.084489844 | 0.996184372 | 2.667963428 | 0 | 0 |
| BRD-A33084557__tioconazole__trt_cp | 10 | 0.112585543 | 0.996289251 | 2.677314325 | 0 | 0 |
| BRD-K32793530__estriol__trt_cp | 11 | 0.110116935 | 0.996438657 | 2.691053058 | 0 | 0 |
| BRD-A22844106__tenoxicam__trt_cp | 85 | 0.040290667 | 0.996484185 | 2.69534254 | 0 | 0 |
| BRD-M30523314__vinorelbine__trt_cp | 66 | 0.07042276 | 0.996526844 | 2.699407243 | 0 | 0 |
| BRD-K58662656__cefepime__trt_cp | 11 | 0.120843471 | 0.996687071 | 2.715085915 | 0 | 0 |
| BRD-K37991163__paroxetine__trt_cp | 127 | 0.068703567 | 0.996693903 | 2.715769471 | 0 | 0 |
| BRD-M94527406__amantadine__trt_cp | 11 | 0.126740638 | 0.996733516 | 2.719758157 | 0 | 0 |
| BRD-K49577446__flunisolide__trt_cp | 111 | 0.049697536 | 0.996769495 | 2.723418792 | 0 | 0 |
| BRD-K12219985__glipizide__trt_cp | 118 | 0.042627344 | 0.996814761 | 2.728076731 | 0 | 0 |
| BRD-K75037734__carbachol__trt_cp | 11 | 0.117867503 | 0.996900057 | 2.737018212 | 0 | 0 |
| BRD-A46747628__ouabain__trt_cp | 65 | 0.149989593 | 0.99699221 | 2.746930939 | 0 | 0 |
| BRD-U61360806__suramin__trt_cp | 23 | 0.086448277 | 0.997003508 | 2.748164999 | 0 | 0 |
| BRD-K23714869__triamcinolone__trt_cp | 10 | 0.116716837 | 0.99704308 | 2.75252077 | 0 | 0 |
| BRD-K09963420__saquinavir__trt_cp | 107 | 0.038510069 | 0.99706279 | 2.754709828 | 0 | 0 |
| BRD-K78485176__olmesartan__trt_cp | 92 | 0.05698763 | 0.997070872 | 2.755611331 | 0 | 0 |
| BRD-A82396632__miconazole__trt_cp | 10 | 0.135486302 | 0.997418793 | 2.796720703 | 0 | 0 |
| BRD-K21548250__moracizine__trt_cp | 64 | 0.050730091 | 0.997463207 | 2.802324202 | 0 | 0 |
| BRD-K70330367__amantadine__trt_cp | 63 | 0.053502338 | 0.997529229 | 2.810820066 | 0 | 0 |
| BRD-K61443650__thiomersal__trt_cp | 8 | 0.183062527 | 0.997572389 | 2.816485542 | 0 | 0 |
| BRD-A41020680__minocycline__trt_cp | 10 | 0.115976127 | 0.997603745 | 2.820659132 | 0 | 0 |
| BRD-K63265447__docetaxel__trt_cp | 37 | 0.084852937 | 0.997619299 | 2.822747772 | 0 | 0 |
| BRD-K47832606__floxuridine__trt_cp | 75 | 0.072609669 | 0.997633735 | 2.824697293 | 0 | 0 |
| BRD-K37694030__doxepin__trt_cp | 123 | 0.040350068 | 0.997683117 | 2.831448716 | 0 | 0 |
| BRD-A73741725__exemestane__trt_cp | 83 | 0.062308384 | 0.99780408 | 2.848553961 | 0 | 0 |
| BRD-K16336526__capsaicin__trt_cp | 37 | 0.071976649 | 0.997862404 | 2.857108824 | 0 | 0 |
| BRD-K59753975__vindesine__trt_cp | 34 | 0.089732026 | 0.997874914 | 2.858971356 | 0 | 0 |
| BRD-K52172416__anastrozole__trt_cp | 60 | 0.04608353 | 0.998019503 | 2.88125158 | 0 | 0 |
| BRD-K30600766__amphotericin-b__trt_cp | 11 | 0.125105197 | 0.998076718 | 2.890477981 | 0 | 0 |
| BRD-A72988804__tiaprofenic-acid__trt_cp | 65 | 0.057443778 | 0.998121872 | 2.897937343 | 0 | 0 |
| BRD-K19706299__MRE-269__trt_cp | 99 | 0.11973849 | 0.998263445 | 2.922430615 | 0 | 0 |
| BRD-K67783091__haloperidol__trt_cp | 111 | 0.042569782 | 0.998328965 | 2.934387207 | 0 | 0 |
| BRD-A55962179__omeprazole__trt_cp | 122 | 0.036791379 | 0.998391149 | 2.946136614 | 0 | 0 |
| BRD-K18135438__chenodeoxycholic-acid__trt_cp | 89 | 0.05259513 | 0.998392395 | 2.946376326 | 0 | 0 |
| BRD-A81772229__simvastatin__trt_cp | 53 | 0.092805189 | 0.998444632 | 2.956577881 | 0 | 0 |
| BRD-K40476324__digoxin__trt_cp | 12 | 0.183905834 | 0.998531796 | 2.97431777 | 0 | 0 |
| BRD-A22684332__procaterol__trt_cp | 90 | 0.054982284 | 0.998578736 | 2.984273209 | 0 | 0 |
| BRD-K71013094__neomycin__trt_cp | 10 | 0.12923754 | 0.998619533 | 2.993172743 | 0 | 0 |
| BRD-K32814891__reboxetine__trt_cp | 23 | 0.114660689 | 0.998668936 | 3.004277139 | 0 | 0 |
| BRD-K18674849__flunisolide__trt_cp | 12 | 0.119662763 | 0.998682814 | 3.007464189 | 0 | 0 |
| BRD-K31484631__rifabutin__trt_cp | 66 | 0.079727745 | 0.998706863 | 3.013060409 | 0 | 0 |
| BRD-M79902621__terbutaline__trt_cp | 9 | 0.128355554 | 0.998797408 | 3.035021731 | 0 | 0 |
| BRD-K66175015__afatinib__trt_cp | 59 | 0.114926774 | 0.998959896 | 3.078535435 | 0 | 0 |
| BRD-K10860596__granisetron__trt_cp | 48 | 0.080045456 | 0.99904832 | 3.104911374 | 0 | 0 |
| BRD-K61341215__vecuronium__trt_cp | 65 | 0.061989869 | 0.999060997 | 3.108875777 | 0 | 0 |
| BRD-K17561142__amiodarone__trt_cp | 309 | 0.056264152 | 0.999072919 | 3.112649307 | 0 | 0 |
| BRD-K59873006__digitoxin__trt_cp | 9 | 0.194074861 | 0.999082226 | 3.115626043 | 0 | 0 |
| BRD-A92439610__triamcinolone__trt_cp | 53 | 0.072655699 | 0.999099365 | 3.121181378 | 0 | 0 |
| BRD-K52020312__metronidazole__trt_cp | 36 | 0.079862962 | 0.99910333 | 3.122480505 | 0 | 0 |
| BRD-U97083655__teicoplanin__trt_cp | 65 | 0.07034222 | 0.999103516 | 3.122541463 | 0 | 0 |
| BRD-K90543092__levonorgestrel__trt_cp | 44 | 0.073266997 | 0.999132088 | 3.132063249 | 0 | 0 |
| BRD-K35559145__levomepromazine__trt_cp | 77 | 0.053494299 | 0.999137959 | 3.134055431 | 0 | 0 |
| BRD-A67479912__progesterone__trt_cp | 53 | 0.056813062 | 0.999138568 | 3.134262774 | 0 | 0 |
| BRD-K89375097__pirenzepine__trt_cp | 72 | 0.052268175 | 0.999164001 | 3.143045702 | 0 | 0 |
| BRD-K49668410__clarithromycin__trt_cp | 48 | 0.065882776 | 0.999175188 | 3.146986869 | 0 | 0 |
| BRD-A48237631__mitomycin-c__trt_cp | 76 | 0.161306763 | 0.999196307 | 3.154563258 | 0 | 0 |
| BRD-K14681867__somatostatin__trt_cp | 67 | 0.07439067 | 0.999220444 | 3.163449882 | 0 | 0 |
| BRD-A09349126__norethindrone__trt_cp | 53 | 0.067515026 | 0.99924588 | 3.173093387 | 0 | 0 |
| BRD-K03222093__cyclosporin-a__trt_cp | 12 | 0.160977573 | 0.999272776 | 3.183622066 | 0 | 0 |
| BRD-K70327167__suramin__trt_cp | 23 | 0.125642908 | 0.999278137 | 3.18576344 | 0 | 0 |
| BRD-K54556307__ethambutol__trt_cp | 10 | 0.13629706 | 0.999290339 | 3.190692513 | 0 | 0 |
| BRD-K30097969__pitavastatin__trt_cp | 46 | 0.142284521 | 0.999352206 | 3.216955433 | 0 | 0 |
| BRD-K49075727__nintedanib__trt_cp | 128 | 0.073529176 | 0.999356784 | 3.218989324 | 0 | 0 |
| BRD-K10916986__vinorelbine__trt_cp | 67 | 0.097654702 | 0.999361983 | 3.221315869 | 0 | 0 |
| BRD-A60414806__vincristine__trt_cp | 61 | 0.09579398 | 0.99939919 | 3.238495114 | 0 | 0 |
| BRD-K47869605__podophyllotoxin__trt_cp | 144 | 0.098614692 | 0.999409009 | 3.243192642 | 0 | 0 |
| BRD-K71059170__cefixime__trt_cp | 60 | 0.097238997 | 0.999412107 | 3.244689918 | 0 | 0 |
| BRD-A79768653__sirolimus__trt_poscon | 161 | 0.150424568 | 0.999422676 | 3.249852829 | 0 | 0 |
| BRD-A01320529__salmeterol__trt_cp | 120 | 0.060320261 | 0.999423431 | 3.25022473 | 0 | 0 |
| BRD-K19796430__erismodegib__trt_cp | 155 | 0.10283187 | 0.999424059 | 3.250534887 | 0 | 0 |
| BRD-K72259270__dyclonine__trt_cp | 10 | 0.131392891 | 0.999439938 | 3.258476 | 0 | 0 |
| BRD-A45889380__mepacrine__trt_cp | 324 | 0.095342088 | 0.999451881 | 3.264587221 | 0 | 0 |
| BRD-K66296774__fluvastatin__trt_cp | 107 | 0.076534405 | 0.999461555 | 3.269627749 | 0 | 0 |
| BRD-K11640013__sulfadimidine__trt_cp | 12 | 0.118206325 | 0.999472541 | 3.275455447 | 0 | 0 |
| BRD-A14208071__oxyphenonium__trt_cp | 46 | 0.076451114 | 0.999486998 | 3.283297138 | 0 | 0 |
| BRD-K82216340__medroxyprogesterone__trt_cp | 88 | 0.0840543 | 0.999492137 | 3.286134132 | 0 | 0 |
| BRD-A53576514__orphenadrine__trt_cp | 75 | 0.057746636 | 0.999492228 | 3.28618444 | 0 | 0 |
| BRD-A70649075__sulconazole__trt_cp | 74 | 0.082669232 | 0.999501624 | 3.291442088 | 0 | 0 |
| BRD-A31521121__methocarbamol__trt_cp | 76 | 0.055462986 | 0.999514549 | 3.298825747 | 0 | 0 |
| BRD-A03323675__ritonavir__trt_cp | 45 | 0.072384884 | 0.999569246 | 3.332237353 | 0 | 0 |
| BRD-A09073503__chlorpromazine__trt_cp | 24 | 0.112093174 | 0.999585623 | 3.343008221 | 0 | 0 |
| BRD-K87116849__cefalotin__trt_cp | 12 | 0.142787416 | 0.999610569 | 3.360199688 | 0 | 0 |
| BRD-A49447682__desoximetasone__trt_cp | 81 | 0.061510773 | 0.999624769 | 3.370447612 | 0 | 0 |
| BRD-K83153774__mirtazapine__trt_cp | 44 | 0.070914809 | 0.999626485 | 3.371710579 | 0 | 0 |
| BRD-K44133266__sulbactam__trt_cp | 8 | 0.184537898 | 0.999631621 | 3.375522033 | 0 | 0 |
| BRD-K15891719__tenofovir__trt_cp | 45 | 0.135109695 | 0.999641131 | 3.382711991 | 0 | 0 |
| BRD-K86882815__cabergoline__trt_cp | 71 | 0.081354671 | 0.999647748 | 3.387820529 | 0 | 0 |
| BRD-K49810818__sorafenib__trt_cp | 241 | 0.063728861 | 0.999648873 | 3.388697844 | 0 | 0 |
| BRD-A33973768__scopolamine__trt_cp | 10 | 0.135600972 | 0.999651389 | 3.390669562 | 0 | 0 |
| BRD-K89210380__biotin__trt_cp | 71 | 0.061107529 | 0.99965924 | 3.396907709 | 0 | 0 |
| BRD-K08845546__tacrolimus__trt_cp | 171 | 0.076309988 | 0.999671468 | 3.406895032 | 0 | 0 |
| BRD-A16746338__podophyllotoxin__trt_cp | 26 | 0.144933899 | 0.999673429 | 3.408528792 | 0 | 0 |
| BRD-K13238168__haloprogin__trt_cp | 12 | 0.167485083 | 0.999679084 | 3.413292033 | 0 | 0 |
| BRD-K39987650__bisacodyl__trt_cp | 93 | 0.095202316 | 0.999687158 | 3.420228788 | 0 | 0 |
| BRD-A42383464__actinomycin-d__trt_cp | 18 | 0.298600233 | 0.999702424 | 3.433814474 | 0 | 0 |
| BRD-K30240666__clemastine__trt_cp | 95 | 0.074659646 | 0.999706017 | 3.437105457 | 0 | 0 |
| BRD-K48300629__zonisamide__trt_cp | 87 | 0.056201419 | 0.999709926 | 3.440729685 | 0 | 0 |
| BRD-A40639672__ketorolac__trt_cp | 128 | 0.050064481 | 0.999725623 | 3.455754635 | 0 | 0 |
| BRD-K22662435__ganciclovir__trt_cp | 56 | 0.067863324 | 0.999729907 | 3.459994471 | 0 | 0 |
| BRD-A22740840__oxytetracycline__trt_cp | 10 | 0.144536877 | 0.999731012 | 3.461098277 | 0 | 0 |
| BRD-A23801136__rifabutin__trt_cp | 11 | 0.140245418 | 0.999747048 | 3.477610909 | 0 | 0 |
| BRD-A62025033__temsirolimus__trt_cp | 173 | 0.144713039 | 0.999755734 | 3.486966613 | 0 | 0 |
| BRD-A96107863__nisoldipine__trt_cp | 35 | 0.094926998 | 0.999759185 | 3.490770808 | 0 | 0 |
| BRD-A31159102__fluoxetine__trt_cp | 133 | 0.066301789 | 0.999768536 | 3.501338174 | 0 | 0 |
| BRD-K65261396__tacrolimus__trt_cp | 62 | 0.0762325 | 0.999775993 | 3.510054911 | 0 | 0 |
| BRD-A26384407__chlortalidone__trt_cp | 142 | 0.038579681 | 0.999787089 | 3.523540754 | 0 | 0 |
| BRD-K92723993__imatinib__trt_cp | 332 | 0.045625578 | 0.999797412 | 3.536689656 | 0 | 0 |
| BRD-K17674993__diflorasone__trt_cp | 64 | 0.101947585 | 0.999798163 | 3.537669549 | 0 | 0 |
| BRD-K64052750__gefitinib__trt_cp | 387 | 0.062178543 | 0.999812488 | 3.557063561 | 0 | 0 |
| BRD-A16332958__modafinil__trt_cp | 110 | 0.052009119 | 0.999813382 | 3.558318411 | 0 | 0 |
| BRD-K91263825__nortriptyline__trt_cp | 74 | 0.093449976 | 0.999813823 | 3.558940315 | 0 | 0 |
| BRD-K60640630__mometasone__trt_cp | 68 | 0.075616208 | 0.999824229 | 3.574020295 | 0 | 0 |
| BRD-A65767837__hydrocortisone__trt_cp | 41 | 0.130671093 | 0.999837963 | 3.595255687 | 0 | 0 |
| BRD-K79124250__ioxaglic-acid__trt_cp | 72 | 0.069944631 | 0.999841389 | 3.600814908 | 0 | 0 |
| BRD-A79465854__auranofin__trt_cp | 235 | 0.107088607 | 0.999847688 | 3.611335294 | 0 | 0 |
| BRD-K54006094__formoterol__trt_cp | 36 | 0.088944029 | 0.999853352 | 3.621150601 | 0 | 0 |
| BRD-K57631554__aminolevulinic-acid__trt_cp | 44 | 0.079477458 | 0.999853978 | 3.622257572 | 0 | 0 |
| BRD-K27853494__phenylpropanolamine__trt_cp | 7 | 0.225018854 | 0.999854613 | 3.623383658 | 0 | 0 |
| BRD-A55594068__vinblastine__trt_cp | 85 | 0.105930765 | 0.999856852 | 3.627394874 | 0 | 0 |
| BRD-A71157293__fursultiamine__trt_cp | 53 | 0.065991227 | 0.999864448 | 3.641451852 | 0 | 0 |
| BRD-K86204871__terconazole__trt_cp | 77 | 0.063082579 | 0.999867931 | 3.648148018 | 0 | 0 |
| BRD-A88254928__salbutamol__trt_cp | 47 | 0.087116181 | 0.999874247 | 3.660721868 | 0 | 0 |
| BRD-K39397585__piperacillin__trt_cp | 12 | 0.133498753 | 0.999876807 | 3.665987932 | 0 | 0 |
| BRD-K76525302__sulindac__trt_cp | 21 | 0.145203645 | 0.999882854 | 3.678846518 | 0 | 0 |
| BRD-M45038741__metoprolol__trt_cp | 12 | 0.145467479 | 0.999897695 | 3.713256112 | 0 | 0 |
| BRD-K32164935__tolazamide__trt_cp | 377 | 0.039400756 | 0.999899808 | 3.718532553 | 0 | 0 |
| BRD-K76723084__isotretinoin__trt_cp | 134 | 0.077454819 | 0.999908573 | 3.741598993 | 0 | 0 |
| BRD-K15010214__emedastine__trt_cp | 12 | 0.138060885 | 0.999910796 | 3.747778006 | 0 | 0 |
| BRD-A48720949__testosterone__trt_cp | 63 | 0.079016688 | 0.999912749 | 3.753328603 | 0 | 0 |
| BRD-K66615216__moxifloxacin__trt_cp | 113 | 0.0549519 | 0.999916375 | 3.763949034 | 0 | 0 |
| BRD-M72681210__eserine__trt_cp | 12 | 0.167580642 | 0.999918009 | 3.768879862 | 0 | 0 |
| BRD-K59058766__chlorprothixene__trt_cp | 83 | 0.098840392 | 0.999923106 | 3.784873036 | 0 | 0 |
| BRD-A63667919__methylergometrine__trt_cp | 66 | 0.076923277 | 0.99992441 | 3.789122998 | 0 | 0 |
| BRD-K49328571__dasatinib__trt_cp | 243 | 0.126186392 | 0.999928342 | 3.802375191 | 0 | 0 |
| BRD-K86887724__dofetilide__trt_cp | 37 | 0.092594661 | 0.999933002 | 3.818996079 | 0 | 0 |
| BRD-K72676686__fluvoxamine__trt_cp | 75 | 0.080501268 | 0.999933759 | 3.821796731 | 0 | 0 |
| BRD-A65449987__flunisolide__trt_cp | 63 | 0.080389492 | 0.999934226 | 3.823541848 | 0 | 0 |
| BRD-A56518012__epirubicin__trt_cp | 14 | 0.299640279 | 0.999934556 | 3.824782021 | 0 | 0 |
| BRD-A97730597__hexylcaine__trt_cp | 74 | 0.093161389 | 0.999937294 | 3.835297007 | 0 | 0 |
| BRD-A50928468__norgestrel__trt_cp | 66 | 0.089651397 | 0.999946123 | 3.872433075 | 0 | 0 |
| BRD-K55055802__BRD-K55055802__trt_cp | 37 | 0.090703324 | 0.999951166 | 3.89631277 | 0 | 0 |
| BRD-A84481105__thioridazine__trt_poscon | 151 | 0.139950634 | 0.999951779 | 3.899375759 | 0 | 0 |
| BRD-U94846492__quinine__trt_cp | 149 | 0.071112294 | 0.999952733 | 3.904207977 | 0 | 0 |
| BRD-K13533483__cyclosporin-a__trt_cp | 71 | 0.10097369 | 0.999958874 | 3.937740235 | 0 | 0 |
| BRD-K98490050__amsacrine__trt_cp | 76 | 0.17412248 | 0.999960305 | 3.946236705 | 0 | 0 |
| BRD-A16311756__profenamine__trt_cp | 63 | 0.077999212 | 0.999960444 | 3.947075576 | 0 | 0 |
| BRD-K99964838__bosutinib__trt_cp | 83 | 0.122765269 | 0.999961462 | 3.953317493 | 0 | 0 |
| BRD-K92093830__doxorubicin__trt_cp | 190 | 0.166902786 | 0.999963305 | 3.96501902 | 0 | 0 |
| BRD-K89732114__trifluoperazine__trt_cp | 486 | 0.065338918 | 0.999963939 | 3.969176739 | 0 | 0 |
| BRD-K17306061__aprepitant__trt_cp | 63 | 0.067117898 | 0.999964285 | 3.97147136 | 0 | 0 |
| BRD-K25433859__maprotiline__trt_cp | 103 | 0.091480197 | 0.999968024 | 3.997732712 | 0 | 0 |
| BRD-K32744045__disulfiram__trt_cp | 46 | 0.104598863 | 0.999975034 | 4.055943333 | 0 | 0 |
| CMAP-C110765__KCl__trt_cp | 544 | 0.122114815 | 0.999978525 | 4.091011187 | 0 | 0 |
| BRD-A51410489__yohimbine__trt_cp | 53 | 0.096810642 | 0.999979042 | 4.096652766 | 0 | 0 |
| BRD-A23770159__sirolimus__trt_cp | 172 | 0.105574747 | 0.999980374 | 4.111834981 | 0 | 0 |
| BRD-K02113016__olaparib__trt_cp | 444 | 0.058706297 | 0.9999816 | 4.126700929 | 0 | 0 |
| BRD-K01292756__pimozide__trt_cp | 266 | 0.09215613 | 0.999981835 | 4.129664649 | 0 | 0 |
| BRD-K60460488__nelfinavir__trt_cp | 65 | 0.116923862 | 0.999982134 | 4.133474288 | 0 | 0 |
| BRD-A16998493__etodolac__trt_cp | 33 | 0.191757476 | 0.999984269 | 4.162623108 | 0 | 0 |
| BRD-K53857191__risperidone__trt_cp | 143 | 0.051503993 | 0.999984803 | 4.170496316 | 0 | 0 |
| BRD-A52530684__doxorubicin__trt_cp | 200 | 0.218882823 | 0.999986384 | 4.195450882 | 0 | 0 |
| BRD-A86044036__flurbiprofen__trt_cp | 91 | 0.065761611 | 0.99998948 | 4.253565012 | 0 | 0 |
| BRD-A38030642__cyclosporin-a__trt_cp | 235 | 0.128535618 | 0.999991077 | 4.290263073 | 0 | 0 |
| BRD-K47780086__penciclovir__trt_cp | 49 | 0.083611099 | 0.999991621 | 4.304226293 | 0 | 0 |
| BRD-A76528577__vincristine__trt_cp | 83 | 0.131556342 | 0.999992117 | 4.317705886 | 0 | 0 |
| BRD-K49759007__phenazone__trt_cp | 34 | 0.124391526 | 0.999993211 | 4.350588622 | 0 | 0 |
| BRD-A68009927__daunorubicin__trt_cp | 190 | 0.207168655 | 0.99999351 | 4.360456422 | 0 | 0 |
| BRD-K41170226__deoxycholic-acid__trt_cp | 113 | 0.113130803 | 0.999993998 | 4.377532721 | 0 | 0 |
| BRD-K21283037__riluzole__trt_cp | 155 | 0.062164159 | 0.999994055 | 4.379592301 | 0 | 0 |
| BRD-K78659596__MLN-2238__trt_cp | 187 | 0.213883004 | 0.999994068 | 4.380083388 | 0 | 0 |
| BRD-K07103434__pyrvinium__trt_cp | 17 | 0.248695236 | 0.999994175 | 4.38403268 | 0 | 0 |
| BRD-K17075857__chloroxine__trt_cp | 95 | 0.099296329 | 0.999994564 | 4.399056573 | 0 | 0 |
| BRD-A90311807__cilastatin__trt_cp | 69 | 0.074461008 | 0.999994612 | 4.400997386 | 0 | 0 |
| BRD-A07875874__cilnidipine__trt_cp | 82 | 0.097158299 | 0.999994743 | 4.406308211 | 0 | 0 |
| BRD-K00603606__ticlopidine__trt_cp | 116 | 0.066500135 | 0.999995381 | 4.434295008 | 0 | 0 |
| BRD-K91966436__daunorubicin__trt_cp | 9 | 0.327560452 | 0.999995639 | 4.446671659 | 0 | 0 |
| BRD-A41519720__ezetimibe__trt_cp | 82 | 0.116741647 | 0.999995693 | 4.449347806 | 0 | 0 |
| BRD-K88510285__bortezomib__trt_cp | 184 | 0.22465975 | 0.999995942 | 4.462083077 | 0 | 0 |
| BRD-K43389675__daunorubicin__trt_cp | 115 | 0.275042636 | 0.999995996 | 4.464943735 | 0 | 0 |
| BRD-K52075040__cerulenin__trt_cp | 210 | 0.146840974 | 0.999996471 | 4.491959153 | 0 | 0 |
| BRD-K51671335__sulpiride__trt_cp | 36 | 0.116528704 | 0.999996492 | 4.493193109 | 0 | 0 |
| BRD-K47029922__tranylcypromine__trt_cp | 24 | 0.171276238 | 0.999996674 | 4.504554233 | 0 | 0 |
| BRD-K35483542__alitretinoin__trt_cp | 155 | 0.087093237 | 0.99999711 | 4.534296663 | 0 | 0 |
| BRD-A84174873__baclofen__trt_cp | 10 | 0.19549978 | 0.999997355 | 4.552975708 | 0 | 0 |
| BRD-K13183738__pentamidine__trt_cp | 48 | 0.159441276 | 0.999997438 | 4.55961775 | 0 | 0 |
| BRD-A64290322__cyclosporin-a__trt_cp | 63 | 0.161896561 | 0.999998687 | 4.698036769 | 0 | 0 |
| BRD-K35189033__levonorgestrel__trt_cp | 102 | 0.072536126 | 0.999998687 | 4.698104963 | 0 | 0 |
| BRD-K69650333__idarubicin__trt_cp | 29 | 0.320444132 | 0.999998786 | 4.714147511 | 0 | 0 |
| BRD-K36627727__tamibarotene__trt_cp | 90 | 0.105223798 | 0.999998986 | 4.75066427 | 0 | 0 |
| BRD-A22783572__vinblastine__trt_cp | 69 | 0.149385168 | 0.99999903 | 4.759662421 | 0 | 0 |
| BRD-K70487031__flupentixol__trt_cp | 64 | 0.11538914 | 0.999999146 | 4.785202371 | 0 | 0 |
| BRD-K44227013__ponatinib__trt_cp | 132 | 0.115482114 | 0.999999222 | 4.803852922 | 0 | 0 |
| BRD-A27489425__rolitetracycline__trt_cp | 49 | 0.14263996 | 0.99999924 | 4.808499706 | 0 | 0 |
| BRD-K78126613__menadione__trt_cp | 404 | 0.091201089 | 0.99999927 | 4.816530797 | 0 | 0 |
| BRD-K51073125__chlorpromazine__trt_cp | 12 | 0.29291114 | 0.999999282 | 4.820047461 | 0 | 0 |
| BRD-K60866521__idelalisib__trt_cp | 15 | 0.208641963 | 0.999999298 | 4.824494439 | 0 | 0 |
| BRD-K78010432__furosemide__trt_cp | 10 | 0.194897634 | 0.99999938 | 4.849287997 | 0 | 0 |
| BRD-K52237148__cefazolin__trt_cp | 11 | 0.201858506 | 0.999999513 | 4.89669696 | 0 | 0 |
| BRD-K32318651__acyclovir__trt_cp | 76 | 0.123473782 | 0.999999649 | 4.960579844 | 0 | 0 |
| BRD-K84036904__testosterone__trt_cp | 37 | 0.160824182 | 0.999999695 | 4.988146713 | 0 | 0 |
| BRD-K24652731__ivermectin__trt_cp | 10 | 0.329239991 | 0.999999742 | 5.020283635 | 0 | 0 |
| BRD-K13296708__rimonabant__trt_cp | 24 | 0.192377952 | 0.999999772 | 5.044375622 | 0 | 0 |
| BRD-A73909368__dactinomycin__trt_cp | 46 | 0.373546045 | 0.999999799 | 5.068250729 | 0 | 0 |
| BRD-A97739905__ketoprofen__trt_cp | 71 | 0.088784917 | 0.99999984 | 5.110889935 | 0 | 0 |
| BRD-A37541666__naloxone__trt_cp | 52 | 0.100059106 | 0.999999865 | 5.143131868 | 0 | 0 |
| BRD-K77175907__calcifediol__trt_cp | 88 | 0.1255334 | 0.999999892 | 5.185681564 | 0 | 0 |
| BRD-K72238567__fludarabine__trt_cp | 47 | 0.403475389 | 0.999999903 | 5.20491526 | 0 | 0 |
| BRD-A89585551__mefloquine__trt_cp | 10 | 0.227078921 | 0.999999907 | 5.212800292 | 0 | 0 |
| BRD-A42167015__carteolol__trt_cp | 75 | 0.083098976 | 0.999999912 | 5.223916033 | 0 | 0 |
| BRD-K70401845__erlotinib__trt_cp | 214 | 0.09899264 | 0.999999918 | 5.236934084 | 0 | 0 |
| BRD-A31374339__fludarabine__trt_cp | 65 | 0.144525307 | 0.999999934 | 5.277378901 | 0 | 0 |
| BRD-K70578146__dactinomycin__trt_cp | 38 | 0.34148083 | 0.999999939 | 5.291900067 | 0 | 0 |
| BRD-A67799922__phenoxybenzamine__trt_cp | 150 | 0.07575119 | 0.999999977 | 5.466667278 | 0 | 0 |
| BRD-A60571864__budesonide__trt_cp | 85 | 0.088135166 | 0.99999998 | 5.494186738 | 0 | 0 |
| BRD-K21680192__mitoxantrone__trt_cp | 343 | 0.272737261 | 0.999999983 | 5.523309506 | 0 | 0 |
| BRD-K62012036__acitretin__trt_cp | 89 | 0.102744068 | 0.999999987 | 5.571749657 | 0 | 0 |
| BRD-K17210248__daunorubicin__trt_cp | 52 | 0.37696345 | 0.99999999 | 5.617068339 | 0 | 0 |
| BRD-K04548931__pidorubicine__trt_cp | 85 | 0.363498226 | 0.999999992 | 5.653468535 | 0 | 0 |
| BRD-K55696337__topotecan__trt_cp | 31 | 0.33161457 | 0.999999993 | 5.683281074 | 0 | 0 |
| BRD-A84327315__calcitriol__trt_cp | 66 | 0.156047966 | 0.999999994 | 5.689594868 | 0 | 0 |
| BRD-A76941896__doxorubicin__trt_cp | 54 | 0.402260181 | 0.999999995 | 5.723868426 | 0 | 0 |
| BRD-K88625236__nonoxynol-9__trt_cp | 63 | 0.184394044 | 0.999999995 | 5.744022867 | 0 | 0 |
| BRD-A59943784__fluticasone__trt_cp | 72 | 0.120744781 | 0.999999997 | 5.796287413 | 0 | 0 |
| BRD-A44780397__mifepristone__trt_cp | 67 | 0.13605863 | 0.999999998 | 5.852474727 | 0 | 0 |
| BRD-K19352500__prochlorperazine__trt_cp | 117 | 0.102222023 | 0.999999998 | 5.865743061 | 0 | 0 |
| BRD-K07237224__moclobemide__trt_cp | 47 | 0.136046116 | 0.999999998 | 5.923681378 | 0 | 0 |
| BRD-A35869383__ibrutinib__trt_cp | 135 | 0.141981975 | 0.999999999 | 5.932039186 | 0 | 0 |
| BRD-K64634304__tretinoin__trt_cp | 197 | 0.117099145 | 0.999999999 | 5.981808583 | 0 | 0 |
| BRD-A59985574__topotecan__trt_cp | 51 | 0.390991921 | 1 | 6.239328665 | 0 | 0 |
| BRD-K70799801__quinidine__trt_cp | 65 | 0.125342601 | 1 | 6.429517482 | 0 | 0 |
| BRD-A64479082__quinidine__trt_cp | 67 | 0.111709391 | 1 | 6.622055202 | 0 | 0 |
